# Supplementary material for: Pragmatic accuracy of an in-house loop-mediated isothermal amplification (LAMP) for diagnosis of pulmonary tuberculosis in a Thai community hospital
Source: PLoS One. 2020 Jul 23;15(7):e0236496. doi: 10.1371/journal.pone.0236496 (PMC7377475; doi:10.1371/journal.pone.0236496)
Supplement: S1 Table — (DOCX) [file pone.0236496.s001.docx]

**Table S1. Review on diagnostic accuracy of in-house LAMP assays for diagnosis of pulmonary tuberculosis**

| **Study** | **Country** | **Setting** | **Primer target or techniques** | **Sensitivity in S+C+**  **(95% CI)** | **Sensitivity in S-C+**  **(95% CI)** | **Sensitivity in C+**  **(95% CI)** | **Specificity**  **in S-C-**  **(95% CI)** |
| --- | --- | --- | --- | --- | --- | --- | --- |
| Aryan  (2013)[1] | Iran | University Hospital | IS6110 | 94.0  (85.4-98.4) | 60.0  (26.2-87.8) | 89.6  (80.5-95.4) | 89.0  (71.0-98.0) |
| Boehme (2007)[2] | Peru, Bangladesh, Tanzania | TB centers | gyrB | 97.7  (95.0-100.0) | 48.8  (34.0-64.0) | 88.2  (83.0-92.0) | 99.0  (98.0-100.0) |
| Dolker  (2012)[3] | India | TB hospital | IS6110 |  |  | 97.0  (94.0-99.0) | 60.0  (47.0-72.0) |
| Fujisaki (2004)[4] | Japan | University Hospital | 16S rRNA gene |  |  | 100.0  (48.0-100.0) | 100.0  (48.0-100.0) |
| George  (2011)[5] | India | College Hospital | 16s rRNA gene | 96.7  (83.0-99.0) | 22.2  (6.0-55.0) | 79.5  (64.0-89.0) | 100.0  (88.0-100.0) |
| Habeenzu  (2017)[6] | Zambia | University Hospital | In-house with PURE kit | 96.9  (89.3-99.6) | 39.1  (19.7-61.5) | 81.4  (71.6-89.0) | 96.5  (91.3-99.0) |
| Hong  (2012)[7] | China | TB hospital | esat6, mtp40 |  |  | 92.0  (64.0-100.0) | 85.0  (66.0-96.0) |
| Kaewphinit (2013)[8] | Thailand | TB laboratory | IS6110 |  |  | 99.0  (94.0-100.0) | 100.0  (63.0-100.0) |
| Kohan  (2011)[9] | Iran | TB center | IS6110 |  |  | 100.0  (94.0-100.0) | 96.0  (88.0-99.0) |
| Lee J  (2009) [10] | Taiwan | University Hospital | 16S rRNA gene |  |  | 94.1  (81.0-98.0) | 94.0  (88.0-97.0) |
| Li  (2014) [11] | China | TB center | IS6110 |  |  | 97.0  (94.0-98.0) | 92.0  (89.0-93.0) |
| Miller  (2013)[12] | Zambia | Chest clinic | NA |  |  | 69.0  (56.0-79.0) | 90.0  (80.0-96.0) |
| Moon  (2015)[13] | Korea | University Hospital | hspX |  |  | 91.0  (77.0-98.0) | 95.0  (92.0-97.0) |
| Nimesh (2014)[14] | India | Hospital | sdaA |  |  | 100.0  (81.0-100.0) | 97.0  (94.0-99.0) |
| Pandey (2008)[15] | Nepal | TB center | 16S rRNA gene | 100.0  (96.0-100.0) | 100.0  (61.0-100.0) | 100.0  (96.0-100.0) | 94.2  (90.0-98.0) |
| Phetsuksiri (2020)[16] | Thailand | National TB laboratory | 16s rRNA gene | 99.0  (94.8-100.0) | 72.7  (49.8-89.3) | 94.4  (88.9-97.7) | 94.3  (87.2-98.1) |
| Poudel  (2009)[17] | Nepal | National TB laboratory | 16S rRNA gene |  |  | 97.0  (92.0-99.0) | 94.1  (88.0-97.0) |
| Rafati  (2015)[18] | Iran | NA | 16s rRNA gene |  |  | 90.0  (55.0-100.0) | 95.0  (83.0-99.0) |
| Sethi  (2013)[19] | India | Chest clinic | 16s rRNA gene | 98.4  (91.7-99.7) | 76.9  (46.2-94.7) | 95.0  (87.0-99.0) | 48.0  (28.0-69.0) |
| Toonkomdang  (This study) | Thailand | Community Hospital | 16s rRNA gene | 90.9  (78.3-97.5) | 16.7  (0.4-64.1) | 82.0  (68.6-91.4) | 94.7  (85.4,98.9) |

**References**

1. Aryan E, Makvandi M, Farajzadeh A, Huygen K, Alvandi A-H, Gouya M-M, et al. Clinical value of IS6110-based loop-mediated isothermal amplification for detection of Mycobacterium tuberculosis complex in respiratory specimens. J Infect. 2013;66: 487–493. doi:10.1016/j.jinf.2013.02.005

2. Boehme CC, Nabeta P, Henostroza G, Raqib R, Rahim Z, Gerhardt M, et al. Operational Feasibility of Using Loop-Mediated Isothermal Amplification for Diagnosis of Pulmonary Tuberculosis in Microscopy Centers of Developing Countries. J Clin Microbiol. 2007;45: 1936–1940. doi:10.1128/JCM.02352-06

3. Dolker S, Verma R, Prakash C, Shamal A. Application of loop mediated isothermal amplification (LAMP) assay as an alternative diagnostic test for rapid tuberculosis diagnosis in limited resource setting. Indian J Anim Sci. 2012;82.

4. Fujisaki R. Development of rapid molecular diagnostic method for tuberculosis based on loop-mediated isothermal amplification. Teikyo Med J. 2004;27: 297–305.

5. George G, Mony P, Kenneth J. Comparison of the Efficacies of Loop-Mediated Isothermal Amplification, Fluorescence Smear Microscopy and Culture for the Diagnosis of Tuberculosis. PLoS ONE. 2011;6. doi:10.1371/journal.pone.0021007

6. Habeenzu C, Nakajima C, Solo E, Bwalya P, Kajino K, Miller M, et al. Evaluation of in-house loop-mediated isothermal amplification for tuberculosis diagnosis compared with Xpert MTB/RIF. J Infect Dev Ctries. 2017;11: 440–444. doi:10.3855/jidc.7730

7. Hong M, Zha L, Fu W, Zou M, Li W, Xu D. A modified visual loop-mediated isothermal amplification method for diagnosis and differentiation of main pathogens from Mycobacterium tuberculosis complex. World J Microbiol Biotechnol. 2012;28: 523–531. doi:10.1007/s11274-011-0843-y

8. Kaewphinit T, Santiwatanakul S, Chansiri K. Loop-mediated Isothermal Amplification (LAMP) technique for the detection of Mycobacterium tuberculosis complex. J Med Health Sci. 2013;20: 13–20.

9. Kohan L, Shahhosseiny MH, Razavi MR, Parivar K, Moslemi E, Werngren J. Evaluation of loop mediated isothermal amplification for diagnosis of *Mycobacterium tuberculosis* complex in clinical samples. Afr J Biotechnol. 2011;10: 5096–5101. doi:10.4314/ajb.v10i26.

10. Lee M-F, Chen Y-H, Peng C-F. Evaluation of reverse transcription loop-mediated isothermal amplification in conjunction with ELISA-hybridization assay for molecular detection of Mycobacterium tuberculosis. J Microbiol Methods. 2009;76: 174–180. doi:10.1016/j.mimet.2008.10.005

11. Li Y, Shi L, Pan A, Cao W, Chen X, Meng H, et al. Evaluation of real-time loop-mediated isothermal amplification (RealAmp) for rapid detection of Mycobacterium tuberculosis from sputum samples. J Microbiol Methods. 2014;104: 55–58. doi:10.1016/j.mimet.2014.06.011

12. Miller M, Habeenzu C, Solo E, Bwalya P, Katemangwe P, Kasakwa K, et al. Latest results of ongoing evaluation of the loop-mediated isothermal amplification (LAMP) assay for the diagnosis of tuberculosis in University Teaching Hospital (UTH), Zambia: P. 1.8. 012 (B). Trop Med Int Health. 2013;18.

13. Moon SH, Kim EJ, Tomono J, Miyamoto S, Mitarai S, Kim DW, et al. Detection of Mycobacterium tuberculosis complex in sputum specimens using a loop-mediated isothermal amplification assay in Korea. J Med Microbiol. 2015;64: 1335–1340. doi:10.1099/jmm.0.000164

14. Nimesh M, Joon D, Varma-Basil M, Saluja D. Development and Clinical Evaluation of sdaA Loop-Mediated Isothermal Amplification Assay for Detection of Mycobacterium tuberculosis with an Approach To Prevent Carryover Contamination. J Clin Microbiol. 2014;52: 2662–2664. doi:10.1128/JCM.00907-14

15. Pandey BD, Poudel A, Yoda T, Tamaru A, Oda N, Fukushima Y, et al. Development of an in-house loop-mediated isothermal amplification (LAMP) assay for detection of Mycobacterium tuberculosis and evaluation in sputum samples of Nepalese patients. J Med Microbiol. 2008;57: 439–443. doi:10.1099/jmm.0.47499-0

16. Phetsuksiri B, Rudeeaneksin J, Srisungngam S, Bunchoo S, Klayut W, Nakajima C, et al. Comparison of Loop-Mediated Isothermal Amplification, Microscopy, Culture, and PCR for Diagnosis of Pulmonary Tuberculosis. Jpn J Infect Dis. 2020;advpub. doi:10.7883/yoken.JJID.2019.335

17. Poudel A, Pandey BD, Lekhak B, Rijal B, Sapkota BR, Suzuki Y. Clinical profiling and use of loop-mediated isothermal amplification assay for rapid detection of Mycobacterium tuberculosis from sputum. Kathmandu Univ Med J KUMJ. 2009;7: 109–114. doi:10.3126/kumj.v7i2.2701

18. Rafati A, Gill P. Microfluidic method for rapid turbidimetric detection of the DNA of Mycobacterium tuberculosis using loop-mediated isothermal amplification in capillary tubes. Microchim Acta. 2015;182: 523–530. doi:10.1007/s00604-014-1354-y

19. Sethi SK, Singh S, Dhatwalia SK, Yadav R, Mewara A, Singh M, et al. Evaluation of In‐House Loop‐Mediated Isothermal Amplification (LAMP) Assay for Rapid Diagnosis of M. tuberculosis in Pulmonary Specimens. J Clin Lab Anal. 2013;27: 272–276. doi:10.1002/jcla.21596
