# Supplementary material for: Pragmatic accuracy of an in-house loop-mediated isothermal amplification (LAMP) for diagnosis of pulmonary tuberculosis in a Thai community hospital
Source: PLoS One. 2020 Jul 23;15(7):e0236496. doi: 10.1371/journal.pone.0236496 (PMC7377475; doi:10.1371/journal.pone.0236496)
Supplement: Attachment — Submitted filename: PONE-D-20-00432_R1_review comments.pdf [file pone.0236496.s004.pdf]

PLOS ONE

Pragmatic accuracy of in-house loop-mediated isothermal amplification (LAMP) for diagnosis of pulmonary tuberculosis in a Thai community hospital

--Manuscript Draft--

**Manuscript Number:**	PONE-D-20-00432R1
**Article Type:**	Research Article
**Full Title:**	Pragmatic accuracy of in-house loop-mediated isothermal amplification (LAMP) for diagnosis of pulmonary tuberculosis in a Thai community hospital
**Short Title:**	Diagnostic accuracy of in-house LAMP for pulmonary TB
**Corresponding Author:**	Phichayut Phinyo<br>Chiang Mai University<br>Chiang Mai, Chiang Mai THAILAND
**Keywords:**	pulmonary tuberculosis; LAMP; Diagnosis; sensitivity; Specificity
**Abstract:**	Background: To improve the quality of diagnosing pulmonary tuberculosis (TB), WHO recommends the use of rapid molecular testing as an alternative to conventional microscopic methods. Loop-mediated isothermal amplification assay (LAMP test) is a practical and cost-effective nucleic amplification technique. We evaluated the pragmatic accuracy of the in-house LAMP assay for the diagnosis of TB in a remote health care setting where an advanced rapid molecular test is not available.<br>Methods: A prospective diagnostic accuracy study was conducted. Patients with clinical symptoms suggestive of TB were consecutively enrolled from April to August 2016. Sputum samples were collected from each patient and were sent for microscopic examination (both acid-fast stain and fluorescence stain), in-house LAMP test, and TB culture.<br>Results: One hundred and seven patients with TB symptoms were used in the final analysis. This included 50 (46.7%) culture-positive TB patients and 57 (53.3%) culture-negative patients. The overall sensitivity of the in-house LAMP based on culture positivity was 88.8% (95%CI 81.2,94.1). The sensitivity was 90.9% (95%CI 78.3,97.5) for smear-positive, culture-positive patients, and was 16.7% (95%CI 0.4,64.1) for smear-negative, culture-positive patients. The overall sensitivity and accuracy of the in-house LAMP test compared to smear microscopy methods were not significantly different (p=0.375 and p=1.000, respectively). The specificity of the in-house LAMP based on non-TB patients (smear-negative, culture-negative) was 94.7% (95%CI 85.4,98.9).<br>Conclusions: The diagnostic accuracy of the in-house LAMP test in a community hospital was comparable to other previous reports in terms of specificity. The sensitivity of the in-house assay could be improved with better sputum processing and DNA extraction method.
**Order of Authors:**	Sarawut Toonkomdang
	Phichayut Phinyo
	Benjawan Phetsuksiri
	Jayanton Patumanond
	Janisara Rudeeaneksin
	Wiphat Klayut
**Response to Reviewers:**	Responses to Reviewers' comments<br>Pragmatic accuracy of loop-mediated isothermal amplification (LAMP)<br>for diagnosis of pulmonary tuberculosis in a Thai community hospital<br><br>Reviewer #1:<br>1.The study aims to evaluate usefulness of a LAMP method in a practical setting in Thailand. The LAMP method is now available as an only commercial kit TB-LAMP assay (Loopamp™MTBC Detection Kit, Eiken Chemical Company Ltd., Japan) as endorsed by WHO in 2016. It seems that the method used in this study is a unique

system at least partially. So, it is important to state explicitly that the target to be evaluated was an in-house LAMP and not one commercially available LAMP recommended by WHO.
oThe LAMP test in our study was a non-commercial, in-house LAMP.
oWe re-wrote the manuscript and emphasized that the test used was in-house LAMP.
2.In evaluating the sensitivity of the method, the authors used culture negative (clinically defined) cases, as well as bacteriologically confirmed cases, as a gold standard of the cases of TB. It may be difficult to admit the clinical diagnosis as a diagnostic basis for such a study as this, apart from clinical practice. Vice versa, the definition of the gold (conventional) standard for specificity (non-cases) should be reconsidered. The following paper may be of use in revising the paper; Kaku et al: Accuracy of LAMP-TB Method for Diagnosing Tuberculosis in Haiti. Jpn. J. Infect. Dis., 69, 488–492, 2016.
oWe modified the inclusion criteria for analysis as suggested by both reviewers.
oAs the analysis was done in a per-patient fashion, patients with smear-positive and culture-negative results would be excluded, as these patients were considered as probable TB cases. Therefore, the evaluation of sensitivity would include patients with both smear positive and smear negative with positive culture results. In contrast, the evaluation of specificity would include only patients with smear-negative and culture-negative results.

Reviewer #2:
1.Abstract/Background: “proven diagnostic performance” – this is both vague and too specific at the same time, “most of the results were validated” – the results aren’t validated, the assay is validated
oWe rewrote the abstract and introduction part as suggested.
2.The language surrounding people with possible TB needs to be updated throughout the paper - avoid the use of terms like "TB suspects" that increase the stigma surrounding this disease.
http://www.stoptb.org/assets/documents/resources/publications/acsm/LanguageGuide_ForWeb20131110.pdf
oWe rewrote the abstract and introduction part as suggested.
3.The paper states repeatedly that there is little work published from resource-challenged settings, but this claim is not supported. Even the references given cite studies in such decentralized settings. Maybe it just hasn’t been done in Thailand? A better summary of the literature needs to be included. How does this compare to other studies? How is the TB LAMP test performed in this study compare to the TB LAMP tests in other published literature? A better focus on properly relating the current study to the body of work in the literature rather than trying to claim it is quite novel would actually strengthen the paper. There is merit in replication or demonstrating an important diagnostic in a new geographical area.
oWe rewrote the abstract and introduction part as suggested.
4.In-house vs commercialized kit is mentioned but not explained. And the position of this paper (what LAMP testing approach is used) is not properly placed in the context of what other papers are using and the potential impact on sensitivity/specificity.
oWe rewrote the abstract and introduction part as suggested.
5.The sensitivity/specificity of LAMP in other papers, settings, etc needs to be stated with numbers and not just alluded to. A proper, specific summary of the literature is lacking.
oWe rewrote the abstract and introduction part as suggested.
6.“In 2016, WHO suggested the use of LAMP assay for the diagnosis of pulmonary tuberculosis” – this is not quite right, WHO recommendations are very specific and it is important to get that right. From the abstract of the citation provided: “WHO recommends that TB-LAMP can be used as a replacement for microscopy for the diagnosis of pulmonary TB in adults with signs and symptoms of TB”. This needs to be stated correctly. Also, given the paper has mentioned in-house vs commercialized kits, it needs to be clarified that the WHO guidance refers only to the Eiken LAMP kit.
oWe rewrote the abstract and introduction part as suggested.
7.“LAMP assay has a low cost per test, does not required advanced technological facilities, and can be routinely practiced in general hospital laboratories [3].” Reference 3 doesn’t support this statement – it doesn’t say anywhere that the LAMP assay has a low cost per test. It says “Costs can be kept to a minimum if testing is limited to specimens from the most high-risk patients based on proper clinical assessments and national testing algorithms based on public health policies.” There are other

publications on the cost of the LAMP assay for TB diagnosis. The authors might explain better the infrastructure/training needed for LAMP based on this reference and others.
oWe rewrote the abstract and introduction part as suggested.
oWe changed the references to the statement as follow: Sohn H. Cost, affordability, and cost-effectiveness of TB-LAMP assay. In: Report to WHO Guideline Development Group Meeting on TB-LAMP Assay. Edn. Geneva: World Health Organization; 2016 and Shete PB, Farr K, Strnad L, Gray CM, Cattamanchi A. Diagnostic accuracy of TB-LAMP for pulmonary tuberculosis: a systematic review and meta-analysis. BMC Infect Dis. 2019;19(1):268. Published 2019 Mar 19. doi:10.1186/s12879-019-3881-y
8.Reference 5 doesn't appear to really relate to the sentences it comes after. Reference 3 would make a lot more sense as it is a detailed overview of TB diagnostics including many molecular diagnostics.
oWe rewrote the abstract and introduction part as suggested.

Setting
1.The paper needs to do more to state what sets this setting apart from (or ties it to) other studies. See the methods section describing setting in reference 22 for how attributes of the specific site can be expressed in the context of the needs of LAMP.
oWe elaborated the character of our setting as suggested:
oLevel of health system: rural
oDistance to reference laboratory: 0 km
oMedian LAMP test workload: 6 (4-10)
oElectricity and backup power: infrequent power outages, power generator (350 Kw) and UPS (2.7 Kw)
oBiosafety cabinet infrastructure: BSC class II
oLaboratory staff: 4 lab technicians, 1 lab assistant
2.Study Design: This is not a cross-sectional design; it is a prospective design. The plan was to prospectively enroll 120 patients.
oWe changed the type of design to prospective diagnostic accuracy study as suggested.
oWe would like to make a constructive argument on this point, as the diagnostic accuracy research is actually cross-sectional study in design. The cross-sectional design is only the type of membership condition, single component of study base, and cross-sectional design can therefore be collected prospectively or retrospectively. We would like to ask you to kindly refer to this reference: Assessment of the accuracy of diagnostic tests: the cross-sectional study by Knottnerus JA, 2003.
Link: https://www.ncbi.nlm.nih.gov/pubmed/14615003
3."New patients who were clinically suspected of 109 pulmonary TB (coughing for more than two weeks with or without hemoptysis), aged more than 18 years old were consecutively invited into the study regardless of nation status." Suggest re-writing to something more like: 'Adults more than 18yrs of age with symptoms indicative of pulmonary TB (coughing…) and no history of TB were consecutively enrolled regardless of national status.' If patients were 'invited' but not enrolled, we need numbers on how many declined.
oWe re-wrote the sentence as suggested: Adult patients aged more than 15 years old with symptoms indicative of pulmonary TB (coughing for more than two weeks with or without hemoptysis) and no history of TB were consecutively enrolled regardless of national status.
4."Samples with contaminated culture results or samples from patients who were previously documented as TB cases were excluded." Were the patients excluded or the samples?
oPatients with previously documented TB cases were excluded.
oPatients with two contaminated or missing culture results were excluded.
Methods
1.A map of which samples were used for what tests would be quite helpful. Highlight if any of the reference tests (smear, LJ culture, MGIT culture) were performed on the same sputum as LAMP.
oConventional macroscopy, LAMP test, and culture were conducted as routinely done.
oAll patients were given three sealed containers for the collection of morning sputum specimens. Of all containers sent to the laboratory, only the one with seemingly adequate sputum, containing both mucoid or mucopurulent characters with a sample volume more than 3 ml, was used for the whole investigation procedures as routinely done. Specimens were sent for smear microscopy with conventional acid-fast bacilli

Powered by Editorial Manager® and ProduXion Manager® from Aries Systems Corporation

(AFB) staining with Ziehl-Neelsen technique and fluorescence acid-fast staining with Auramine O solution.
2.Make it clear somewhere that smear-negative refers to AFB smear-negative.
oWe added detail on the smear-negative status as suggested.
oAccording to WHO definitions, any patient with at least two AFB smears of scanty grade or one or more smears of 1+ or more was defined as smear-positive case. Smear-negative case was conversely defined.
3.Study size estimation
This has no purpose here – the study is done. Sample size estimation is for study planning purposes, for securing funding and making sure the plan has statistical validity.
oThe study size estimation part was removed as suggested.
4.Statistical analysis. The first four sentences are unnecessary.
oThe first four sentences were removed as suggested.
5.The authors need to state what method was used to obtain the 95% CI for the sens/spec/PPV/NPV/LR+. It is clear from my testing that the Clopper Pearson binomial exact test was used, the authors should include the reference (usually found in the software documentation).
oThe 95% confidence intervals were calculated using the Clopper Pearson binomial exact method.
oWe added this statement in the statistical section and added the citation as suggested.
6.Kappa statistics are for inter-reader reliability, not for comparison of correlations between tests. It includes the concept that agreement may happen by chance when two people are guessing. However, it is not appropriate for comparison of diagnostic results because there isn't guessing – the samples should not agree by chance but because they are or are not TB and the sensitivities of tests objectively vary. Spearman's correlation can be used, but I think what you actually want is McNemar's test. The desire is to compare the diagnostic performance (i.e. accuracy) between tests – McNemar's test will do that. Alternatively, Spearman's correlation can look at the [objective] agreement between tests.
oSpearman's rank correlation was inserted into the manuscript to represent the objective agreement between tests as suggested.
oThe agreement of LAMP test with smear microscopy methods was analyzed with Kappa's statistics and Spearman's rank correlation.
oWe still presented the value of Kappa's statisti[illegible]s many of the previous studies on LAMP assay and other diagnostic tests had don[illegible]-3].

Results
1.Table 1 is dedicated to showing the patient clinical characteristics by culture status. The p-values shown test whether these characteristics differ significantly dependent on culture status. It is expected that gender, nationality, and age should not differ. Whereas it is also expected that chest x-rays and sputum quality would differ. The baseline demographic data between culture188 positive and negative patients were comparable except for the presence of cavitary lesions on 189 chest radiographs and the character of collected sputum (Table 1). Age, nationality, and gender are demographic data. Chest x-ray and sputum quality are clinical characteristics.
oWe reanalyzed all the data after exclusion of patients with probable TB (LAMP test positive and AFB smear positive patients with negative culture).
oAll the baseline demographic and clinical characteristics data were reanalyzed and presented in Table 1.
oThe statements in the results section were re-written as suggested.
2.Table 2 – re-check the NPV for parallel testing
oWe reanalyzed all the data after exclusion of patients with probable TB (LAMP test positive and AFB smear positive patients with negative culture).
oAll the data on Table 2 were checked for any error as suggested.
3.There are a lot of LAMP-positive and AFB smear-positive patients with negative culture. Especially given that the tests are done on different sputum samples, these should be considered patients with probable TB and not used in assessing sensitivity and specificity.
oWe reanalyzed all the data after exclusion of patients with probable TB (LAMP test positive and AFB smear positive patients with negative culture).
oThe final study size for analysis of LAMP test diagnostic accuracy was therefore 107 patients. (8 patients were excluded, 6 patients with both LAMP test and AFB smear-

Powered by Editorial Manager® and ProduXion Manager® from Aries Systems Corporation

positive and culture negative, 1 patient with AFB positive and culture negative, and 1 patient with fluorescence stain positive and culture negative)
4.There are too few smear-negative, culture-positive patients to assess sensitivity. Specificity should not be stratified by smear status, only sensitivity. For the reason above (that smear-positive, culture-negative patients shouldn't be included in estimations of sensitivity/specificity of LAMP), what the paper is calling 'smear-negative specificity' should in fact be reported as the actual specificity of LAMP.
oWe exclude smear-positive, culture negative patients from the analysis as suggested.
oWe reported the actual specificity of LAMP test without stratification.
oWe acknowledged that our there are too few smear negative, culture positive patients to assess sensitivity in the discussion part.
5.Table 2 – the p-values shown have no real meaning! If you want to compare accuracy of tests, you cannot do a p-value over the final accuracy measures among a bunch of tests. You need to compare tests 1 against another by using 2x2 grids and McNemar's test. So, if you want to compare the accuracy of LAMP to the accuracy of AFB stain, you use the grid in Table 3 and McNemar's test:
oThe comparison of diagnostic indices between LAMP test and AFB, fluorescence stain was re-analyzed using McNemar's exact probability test as suggested. We presented the result of the pairwise tests separately and reformatted Table 2.
oPairwise testing was not performed to compare the specificity between the LAMP test and the smear microscopy methods as the specificity of the latter was affected by incorporation bias and would not be comparable to the in-house LAMP.
oTable 3 was also reformatted.
oSpearman's rank correlation was used as suggested.

Discussion
1."This study had demonstrated the pragmatic performance of the LAMP test, which was comparable to that of the conventional smear microscopy and the fluorescence microscopy." Not true, the performance of LAMP as evaluated in this study was below that of smear microscopy.
oWe rewrote the discussion part as suggested.
o"This study had demonstrated the pragmatic diagnostic performance of the in-house LAMP assay in a remote hospital of a high TB burden country. It was revealed that the overall sensitivity of the in-house LAMP in our study was lower than the numbers reported in the majority of the previous in-house LAMP studies. Nonetheless, the specificity was comparable to other figures reported in literature. In comparison to microscopy methods, the AFB and fluorescence stain, the in-house LAMP was found to be inferior in terms of overall sensitivity (82.0% vs. 88.0%, p=0.375) and accuracy (88.8% vs. 94.4%, p=1.000); however, the comparative statistical test revealed non-significant results. Based on the result of our study, we suggest that the in-house LAMP should not be a substitute to conventional smear methods, but should be done in parallel, which would result in a higher sensitivity with fewer false-negative TB cases."
2."Although the sensitivity and specificity of the LAMP test were lower than that of the acid-fast stain and the fluorescence stain, the comparative statistical test revealed non-significant results" This is still true when McNemar's test is performed, but the right statistical tests need to be used in the paper. Furthermore, a non-significant result doesn't mean no difference, it means the difference is likely smaller than the power of the study to detect.
oWe rewrote the discussion part as suggested.
oWe reanalyzed our data using McNemar's exact probability test as suggested.
3.Put PPV/NPV in the context of the local prevalence of disease! State from the literature or reliable source what the prevalence of TB is in the hospital's area of Thailand. I would suggest giving the readers an example: Given that prevalence and a group of 1000 patients, state how many would be true positives, false positive, true negatives, and false negatives. You can therefore assess what burden the different accuracies will place on the hospital. I.e. if the specificity is quite low and the sensitivity is higher, is that better? If the sensitivity is high and the specificity is lower, is that better? Relate this to the LR+.
oWe would like to make a constructive argument to this question as follow: The prevalence of culture-positive TB in this study was 46.7%. As this was a "consecutive recruitment of patients with sign and symptoms suggestive of pulmonary TB" or "patients with higher pre-test probability that the general prevalence" or the "person

that the in-house LAMP test was intended to be used", the calculation of positive predictive values could be directly calculated and reported from the study data as in the other study [1]. Moreover, both the in-house LAMP assay and acid-fast stain were not intended to be used as screening tests in the general population. For this reason, we did not include this part in our manuscript; however, we provide the answer to the question in this response paper.

oThe latest Maesot's population figures from the Health Data Center (HDC), the ministry of public health, Thailand, was 115,108 in 2019. The prevalence of pulmonary tuberculosis was 351 per 100,000 or 35 per 10,000.

TB caseNon-TB caseTotal

LAMP positive29528557PPV 29/557=5.2%

LAMP negative69,4379,443NPV 9437/9443=94.9%

Total359,96510,000Prevalence=0.0035

4. "In the clinical context of TB diagnosis, both the LAMP test and the smear microscopy are considered as a diagnostic test which would normally be done in TB suspects with high pre-test probability [14]" – this is not what the reference says.

oThe reference states "The TB LAMP assay is usually applied for TB-suspected patients and is rarely used for screening purpose. To rule-in the TB diagnosis, specificity is more important than sensitivity."

oWhat we're trying to imply from this statement was that the LAMP test was developed to be applied for patients who were suspicious of having TB with "higher pre-test probability than average person". As the LAMP test was not for screening purpose, specificity is more important and should be more focused than sensitivity.

oAfter we re-analyzed the data with the exclusion of probable TB cases, our specificity increased to comparable level with previous studies. The parallel and serial testing was omitted from our analysis as the test accuracy of combination of the in-house LAMP with other smear microscopy methods would be seriously affected by incorporation bias (smear-positive, culture-negative patients were all excluded.

5. "Therefore, a serial test relying on both the result from the LAMP test and the acid-fast stain would be more appropriate for use as a rule-in test as it carried higher specificity and positive likelihood ratio than other methods." Authors should define 'rule-in' test and what is generally expected of such a test. Should note the increased cost of such an approach.

oAfter we re-analyzed the data with the exclusion of probable TB cases, our specificity increased to comparable level with previous studies. The parallel and serial testing was omitted from our analysis as the test accuracy of combination of the in-house LAMP with other smear microscopy methods would be seriously affected by incorporation bias (smear-positive, culture-negative patients were all excluded.

6.The effect of a gold standard which is not itself perfect should be discussed. Also the variability between sputum samples should be discussed.

oThe use of routine TB culture as a reference standard might be inadequate, as some TB patients could be classified as not having TB [6]. Different culture media and techniques could be used in composite to achieve different performance characteristics[4]. With a higher quality reference standard, the sensitivity of the in-house LAMP should be increased when a portion of three remaining false-positive cases was re-classified as true-positive cases.

oThis study had a higher proportion of salivary sputum than mucous sputum. This could affect the diagnostic performance of both the index and the reference test[5]. The percentage of culture-positive TB cases was lower in salivary samples than in mucous samples (35.8% vs. 65.0%, p=0.005). Both the quality and quantity of sputum specimens were associated with positivity of smear, molecular testing methods (Xpert MTB/RIF and PCR), and TB culture [6,7]. Thus, it was possible that some patients with pulmonary TB might be classified as smear-negative, LAMP-negative, or even culture-negative cases. Interestingly, it was revealed from our data that the proportion of smear-positive, LAMP-positive results was also significantly lower in salivary sputum than in mucous sputum (31.3% vs 57.5%, p=0.009 and 29.9% vs. 60.0%, p=0.003, respectively). Therefore, the sensitivity and accuracy of all tests, including LAMP, might be underestimated. Previous studies reported that by improving the sputum quality, TB diagnostic yield increased[8,9]. Therefore, high-quality sputum collection must be encouraged both in practice and studies.

7.A better look at the differences between this study and others with better test performance needs to be done.

oIn this study, the sensitivity of the in-house LAMP test was 82.0% (95%CI 68.6-91.4) in culture-positive TB patients, respectively. In the past, several studies had reported a

Powered by Editorial Manager® and ProduXion Manager® from Aries Systems Corporation

	higher sensitivity of the in-house LAMP test, which ranges from 90.0 to 100.0%. Most of these studies were either University hospital, TB-specialized centers or hospitals, or national TB-specialized laboratory, which were generally equipped with highly-trained personnel and adequate infrastructural supports. The overall sensitivity of our in-house LAMP was consistent with two previous studies from India and Zambia, which was 79.5% (95%CI 64.0-89.0) and 81.4% (95%CI 71.6-89.0), respectively. Although both studies were performed in University hospitals, the LAMP procedures were modified to suit local conditions, and sputum processing and DNA extraction was done with commercial kits. The higher sensitivity of the acid-fast stain and the fluorescence stain in our study could be explained by the high prevalence of TB, the absence of HIV patient or a smaller number of patients with paucibacillary sputum, and the availability of skilled technicians<br>8."Currently, the WHO only supported the use of two rapid molecular tests for the diagnosis of 294 pulmonary tuberculosis, which were Xpert MTB/RIF and the LAMP test" – as the concept of LAMP test from a kit and other LAMP tests has been raised, and the variability of accuracy depending, it needs to be clear that the WHO recommendation is only for the Eiken LAMP test kit!<br>oWe edited the statement as follow: "Currently, the WHO only supported the use of two rapid molecular tests for the diagnosis of pulmonary tuberculosis, which were Xpert MTB/RIF and the commercialized TB-LAMP assay".<br><br>References<br>1. George G, Mony P, Kenneth J. Comparison of the Efficacies of Loop-Mediated Isothermal Amplification, Fluorescence Smear Microscopy and Culture for the Diagnosis of Tuberculosis. PLoS ONE. 2011;6. doi:10.1371/journal.pone.0021007<br>2. Phetsuksiri B, Rudeeaneksin J, Srisungngam S, Bunchoo S, Klayut W, Nakajima C, et al. Comparison of Loop-Mediated Isothermal Amplification, Microscopy, Culture, and PCR for Diagnosis of Pulmonary Tuberculosis. Jpn J Infect Dis. 2020;advpub. doi:10.7883/yoken.JJID.2019.335<br>3. Wang Z, Sun H, Ren Z, Xue B, Lu J, Zhang H. Feasibility and Performance of Loop-Mediated Isothermal Amplification Assay in the Diagnosis of Pulmonary Tuberculosis in Decentralized Settings in Eastern China. BioMed Res Int. 2019;2019. doi:10.1155/2019/6845756<br>4. Cudahy P, Shenoi S. Diagnostics for pulmonary tuberculosis. Postgrad Med J. 2016;92: 187–193. doi:10.1136/postgradmedj-2015-133278<br>5. Shi J, Dong W, Ma Y, Liang Q, Shang Y, Wang F, et al. GeneXpert MTB/RIF Outperforms Mycobacterial Culture in Detecting Mycobacterium tuberculosis from Salivary Sputum. In: BioMed Research International [Internet]. 2018 [cited 12 Dec 2019]. Available: https://www.hindawi.com/journals/bmri/2018/1514381/<br>6. Ho J, Marks GB, Fox GJ. The impact of sputum quality on tuberculosis diagnosis: a systematic review. Int J Tuberc Lung Dis Off J Int Union Tuberc Lung Dis. 2015;19: 537–544. doi:10.5588/ijtld.14.0798<br>7. Yoon SH, Lee NK, Yim JJ. Impact of sputum gross appearance and volume on smear positivity of pulmonary tuberculosis: a prospective cohort study. BMC Infect Dis. 2012;12: 172. doi:10.1186/1471-2334-12-172<br>8. Hirooka T, Higuchi T, Tanaka N, Ogura T. [The value of proper sputum collection instruction in detection of acid-fast bacillus]. Kekkaku. 2004;79: 33–37.<br>9. Sicsú AN, Salem JI, Fujimoto LBM, Gonzales RIC, Cardoso M do S de L, Palha PF. Educational intervention for collecting sputum for tuberculosis: a quasi-experimental study 1. Rev Lat Am Enfermagem. 2016;24. doi:10.1590/1518-8345.0363.2703
**Additional Information:**	
**Question**	**Response**
**Financial Disclosure**<br><br>Enter a financial disclosure statement that describes the sources of funding for the work included in this submission. Review the submission guidelines for detailed requirements. View published research articles from *PLOS ONE* for specific	The author(s) received no specific funding for this work.

Powered by Editorial Manager® and ProduXion Manager® from Aries Systems Corporation

examples.<br><br>This statement is required for submission and **will appear in the published article** if the submission is accepted. Please make sure it is accurate.<br><br>**Unfunded studies**<br>Enter: *The author(s) received no specific funding for this work.*<br><br>**Funded studies**<br>Enter a statement with the following details:<br>• Initials of the authors who received each award<br>• Grant numbers awarded to each author<br>• The full name of each funder<br>• URL of each funder website<br>• Did the sponsors or funders play any role in the study design, data collection and analysis, decision to publish, or preparation of the manuscript?<br>• **NO** - Include this sentence at the end of your statement: *The funders had no role in study design, data collection and analysis, decision to publish, or preparation of the manuscript.*<br>• **YES** - Specify the role(s) played.<br><br>* typeset	
**Competing Interests**<br><br>Use the instructions below to enter a competing interest statement for this submission. On behalf of all authors, disclose any competing interests that could be perceived to bias this work—acknowledging all financial support and any other relevant financial or non-financial competing interests.<br><br>This statement **will appear in the published article** if the submission is accepted. Please make sure it is accurate. View published research articles from *PLOS ONE* for specific examples.	The authors have declared that no competing interests exist.

**NO authors have competing interests**<br><br>Enter: *The authors have declared that no competing interests exist.*<br><br>**Authors with competing interests**<br><br>Enter competing interest details beginning with this statement:<br><br>*I have read the journal's policy and the authors of this manuscript have the following competing interests: [insert competing interests here]*<br><br>* typeset	
**Ethics Statement**<br><br>Enter an ethics statement for this submission. This statement is required if the study involved:<br><br>• Human participants<br>• Human specimens or tissue<br>• Vertebrate animals or cephalopods<br>• Vertebrate embryos or tissues<br>• Field research<br><br>Write "N/A" if the submission does not require an ethics statement.<br><br>General guidance is provided below. Consult the submission guidelines for detailed instructions. **Make sure that all information entered here is included in the Methods section of the manuscript.**	This study was approved by the Research Ethics Committee of Maesot General Hospital, The Ministry of Public Health (serial number 37/2015) and The Human Research Ethics Committee of Thammasat University, Faculty of Medicine (COA number 081/2016). The clinical samples used in this study were collected from all patients as routinely done. Informed consent was obtained from all patients prior to inclusion.

Powered by Editorial Manager® and ProduXion Manager® from Aries Systems Corporation

**Format for specific study types**<br><br>**Human Subject Research (involving human participants and/or tissue)**<br>• Give the name of the institutional review board or ethics committee that approved the study<br>• Include the approval number and/or a statement indicating approval of this research<br>• Indicate the form of consent obtained (written/oral) or the reason that consent was not obtained (e.g. the data were analyzed anonymously)<br><br>**Animal Research (involving vertebrate animals, embryos or tissues)**<br>• Provide the name of the Institutional Animal Care and Use Committee (IACUC) or other relevant ethics board that reviewed the study protocol, and indicate whether they approved this research or granted a formal waiver of ethical approval<br>• Include an approval number if one was obtained<br>• If the study involved *non-human primates*, add *additional details* about animal welfare and steps taken to ameliorate suffering<br>• If anesthesia, euthanasia, or any kind of animal sacrifice is part of the study, include briefly which substances and/or methods were applied<br><br>**Field Research**<br><br>Include the following details if this study involves the collection of plant, animal, or other materials from a natural setting:<br>• Field permit number<br>• Name of the institution or relevant body that granted permission	
**Data Availability**<br><br>Authors are required to make all data underlying the findings described fully available, without restriction, and from the time of publication. PLOS allows rare exceptions to address legal and ethical concerns. See the PLOS Data Policy and FAQ for detailed information.	Yes - all data are fully available without restriction

A Data Availability Statement describing where the data can be found is required at submission. Your answers to this question constitute the Data Availability Statement and **will be published in the article**, if accepted.<br><br>**Important:** Stating 'data available on request from the author' is not sufficient. If your data are only available upon request, select 'No' for the first question and explain your exceptional situation in the text box.<br><br>Do the authors confirm that all data underlying the findings described in their manuscript are fully available without restriction?	
**Describe where the data may be found in full sentences. If you are copying our sample text, replace any instances of XXX with the appropriate details.**<br><br>• If the data are **held or will be held in a public repository**, include URLs, accession numbers or DOIs. If this information will only be available after acceptance, indicate this by ticking the box below. For example: *All XXX files are available from the XXX database (accession number(s) XXX, XXX.*).<br>• If the data are all contained **within the manuscript and/or Supporting Information files**, enter the following: *All relevant data are within the manuscript and its Supporting Information files.*<br>• If neither of these applies but you are able to provide **details of access elsewhere**, with or without limitations, please do so. For example:<br><br>*Data cannot be shared publicly because of [XXX]. Data are available from the XXX Institutional Data Access / Ethics Committee (contact via XXX) for researchers who meet the criteria for access to confidential data.*<br><br>*The data underlying the results presented in the study are available from (include the name of the third party*	All relevant data are within the manuscript and its Supporting Information files.

and contact information or URL).<br>• This text is appropriate if the data are owned by a third party and authors do not have permission to share the data.<br><br>* typeset	
Additional data availability information:	

Manuscript Click here to access/download;Manuscript;Manuscript.docx

Pragmatic accuracy of in-house loop-mediated isothermal amplification (LAMP) for diagnosis of pulmonary tuberculosis in a Thai community hospital

Short title: Diagnostic accuracy of in-house LAMP for pulmonary TB

Sarawut Toonkomdang[1], Phichayut Phinyo[2,3*], Benjawan Phetsuksiri[4], Jayanton Patumanond[3], Janisara Rudeeaneksin[4¶], Wiphat Klayut[4¶]

[1] Department of Medical Technology, Maesot General Hospital, Tak, Thailand.

[2]Department of Family Medicine, Faculty of Medicine, Chiang Mai University, Chiang Mai, Thailand.

[3] Center for Clinical Epidemiology and Clinical Statistics, Faculty of Medicine, Chiang Mai University, Chiang Mai, Thailand.

[4] National Institute of Health, Department of Medical Sciences, Ministry of Public Health, Nonthaburi, Thailand.

*Corresponding author:

E-mail: phichayutphinyo@gmail.com (PP)

[¶]These authors contributed equally to this work.

Abstract

Background: To improve the quality of diagnosing pulmonary tuberculosis (TB), WHO recommends the use of rapid molecular testing as an alternative to conventional microscopic methods. Loop-mediated isothermal amplification assay (LAMP test) is a practical and cost-effective nucleic amplification technique. We evaluated the pragmatic accuracy of the in-house LAMP assay for the diagnosis of TB in a remote health care setting where an advanced rapid molecular test is not available.

Methods: A prospective diagnostic accuracy study was conducted. Patients with clinical symptoms suggestive of TB were consecutively enrolled from April to August 2016. Sputum samples were collected from each patient and were sent for microscopic examination (both acid-fast stain and fluorescence stain), in-house LAMP test, and TB culture.

Results: One hundred and seven patients with TB symptoms were used in the final analysis. This included 50 (46.7%) culture-positive TB patients and 57 (53.3%) culture-negative patients. The overall sensitivity of the in-house LAMP based on culture positivity was 88.8% (95%CI 81.2,94.1). The sensitivity was 90.9% (95%CI 78.3,97.5) for smear-positive, culture-positive patients, and was 16.7% (95%CI 0.4,64.1) for smear-negative, culture-positive patients. The overall sensitivity and accuracy of the in-house LAMP test compared to smear microscopy methods were not significantly different (p=0.375 and p=1.000, respectively). The specificity of the in-house LAMP based on non-TB patients (smear-negative, culture-negative) was 94.7% (95%CI 85.4,98.9).

Conclusions: The diagnostic accuracy of the in-house LAMP test in a community hospital was comparable to other previous reports in terms of specificity. The sensitivity of the in-house assay could be improved with better sputum processing and DNA extraction method.

Keywords: Pulmonary Tuberculosis, in-house LAMP, Diagnosis, Sensitivity, Specificity

Introduction

Tuberculosis (TB), an airborne communicable disease, has long been considered a significant threat to global public health. According to The World Health Organization (WHO), 10 million people were newly infected with TB in 2018. Although the incidence and prevalence of TB vary greatly across the globe, 87% of total cases resided within 30 countries with high TB burden, including Thailand, where the incidence rate was 153 cases per 100,000 population in 2018[illegible]. Early diagnosis and timely treatment is an essential component of The End TB Strategy endorsed by the WHO, aiming to end the global TB epidemic by the year 2035 [2]. However, TB is still underdiagnosed and undertreated, especially in resource-limitin[illegible]untries due to the lack of highly sensitive and specific diagnostic tools which are usually expensive and require adequate infrastructure [1,3]. Novel diagnostic methods with enough simplicity and cost-effectiveness [illegible] therefore necessary to improve accurate identification of TB patients in these particular settings [3,4].

Molecular testing methods such as polymerase chain reaction (PCR) or Xpert MTB/RIF have been widely acknowledged as alternative tools [illegible] the diagnosis of TB patients [3,5]. These nucleic amplification techniques were known for yielding rapid and accurate TB diagnosis, which would overcome the limitations of classical methods, insensitivity for smear microscopy, and lengthy incubation period for TB culture. However, several obstacles remain for the application of these molecular tests as point-of-care testing in community settings because of their complexity in executio[illegible]nd substantial requirements for financial and personnel resources [3,6]. Loop-mediated isothermal amplification (LAMP) assay is another recently developed nucleic acid amplification technique. Unlike PCR, where the amplification of DNA fragment occurs in temperature-dependent steps, the reaction of LAMP assay functions in isothermal or constant temperature conditions [7,8]. In 2016, WHO

sugge[illegible] the use of commercial TB-LAMP assay (Eiken Chemical Co., Tokyo, Japan) as a replacement for smear microscopy for the diagnosis of TB in patients with symptoms suggestive of TB [9]. TB-LAMP assay has a low cost per test, does not required advanced technological facilities, and can be routinely practiced in general hospital laboratories [6,10].

As financial resources are usually limited in countries with high TB prevalence, setting up an infrastructure to support the commercial TB-LAMP could still be unattainable. A more affordable in-house LAMP was developed in 2008 [11]. The main advantage of the in-house assay was that it could be impl[illegible]ed on the readily-available infrastructure of any laboratory, even in the decentralized [illegible] However, it did require extra-training and skill of technicians to process the clinical specimens. In the past decades, several clinical studies and meta-analyses had evaluated the diagnostic accuracy of the in-house LAMP [illegible] for the diagnosis of pulmonary TB [12–14] (S1 Table). From the latest meta-analysis, the overall sensitivity and specificity of the in-house LAMP [illegible]s 93.0% (95%CI 88.9-95.7) and 91.8% (95%CI 86.4-95.1), respectively [14]. One recent study in Thailand reported the sensitivity and the specificity of the in-house LAMP at 94.4% (95%CI 88.9-97.7) and 94.3% (95%CI 87.2-98.1), respectively [15]. However, the reported accuracy could be overestimated if being assessed in qualified laboratories with highly skilled technicians and sufficient resources where molecular tests usually are available [14]. Therefore, this study aimed to evaluate the pragmatic accuracy of the in-house LAMP assay for the diagnosis of pulmonary TB in a peripheral community hospital of a developing country with a high TB burden.

Materials and Methods

Ethics Statement

This study was approved by the Research Ethics Committee of Maesot General Hospital, The Ministry of Public Health (serial number 37/2015) and The Human Research Ethics Committee of Thammasat University, Faculty of Medicine (COA number 081/2016). The clinical samples used in this study were collected from all patients as routinely done. Informed consent was obtained from all patients prior to inclusion.

Setting

The study was settled in Maesot General Hospital, a large-sized community hospital with 365 in-patient beds. The hospital is located in Maesot district in Tak (province), which shares the border with Myanmar and provides standard health care to both Thai and non-Thai patients (Burmese immigrants and ethnic minorities). According to the Health Data Center, the ministry of public health, Thailand, the incidence rate of pulmonary TB in Maesot was 351 per 100,000 in 2019. The level of health care system of the hospital is considered rural. Maesot hospital has its own reference laboratory with biosafety cabinet infrastructure, BSC class II. There are four lab technicians and one lab assistant within each working shift. Power generator (350 kW) and UPS (2.7 kW) were available in case of power outages, which was infrequent. Median LAMP test workload per day was 6 (range 4-10).

Study Design

This prospective diagnostic accuracy research was conducted from April to August 2016. Adult patients aged more than 15 years old with symptoms indicative of pulmonary TB

(coughing for more than two weeks with or without hemoptysis) and no history of TB were consecutively enrolled regardless of nationality status. Patients with previously documented TB history or patients with two contaminated or missing cultures were excluded from the study.

Methods

All patients were given three sealed containers for the collection of morning sputum specimens. Of all containers sent to the laboratory, only the one [illegible]th seemingly adequate sputum containing both mucoid or mucopurulent characters with a sample volume of more than 3 ml, was used for the whole investigation procedures as routinely done. Specimens were sent for smear microscopy with conventional acid-fast bacilli (AFB) staining with Ziehl-Neelsen technique and fluorescence acid-fast staining with Auramine O solution. Smear-positive case was defined according to WHO definitions as the presence of at least two smears of scanty grade or one or more smears of 1+ or more. A smear negative case or AFB smear-negative was conversely defined

Sputum decontamination and culture examination

For the sputum decontamination process, the collected samples and 2% N-Acetyl-L-cysteine (NALC) NaOH were poured into a 50 ml sterile centrifuge tube in an equal proportion and were subsequently mixed by vortexing for 30 seconds and left at room temperature (20-25 °C) for 15 minutes. Then, the test tubes were filled with phosphate buffer saline (pH 6.8) until the volume reached the level of 50 ml. The samples were put in a high-speed refrigerated centrifuge at 3,000 g for 20 minutes. Next, the supernatants were poured off, leaving the tube

with decontaminated sputum samples. Finally, a drop (1 ml) of phosphate buffer saline (pH 6.8) was used for resuspension of the specimen.

For TB culture, the reference test, we performed both conventional culture method on L-J (Lowenstein-Jensen) medium and BBL MGIT 960 (mycobacterial growth indicator tube) culture method. The culture media were inoculated with processed sputum specimens and incubated at 35 to 37℃ and monitored weekly for growth until 8 weeks. The sputum samples were considered as “culture-positive” if growth was detected in either of L-J or MGIT culture, regardless of the smear status. If growth was not detected in neither of the culture methods and both microscopy results were negative, the samples were considered as “culture-negative” or “non-TB patients”. Patients with smear-positive and culture-negative, which were generally considered as probable TB, were excluded from the analysis. Both smear microscopy and culture methods were performed according to the standard protocols [16].

In-house LAMP test

The LAMP test consists of three steps as follows: DNA extraction, isothermal amplification, and visual interpretation with fluorescence. The National Institute of Health of Thailand had developed the TB Fast Amp technique (a modified LAMP procedure) to suite local practice since 2009. The procedures were described as follow. Flexi Gene® DNA Kit (Qiagen co., USA) and Protenase K Kit (Qiagen co., USA) were used for DNA extraction [17,18]. Four primers (MTB primers, MAV primers, MIN primers, and Muniv primers) were used for the recognition of six distinct regions on the 16S ribosomal RNA gene of M. *tuberculosis*. Each single LAMP reaction includes 12 µl of TB-Fast AMP mixture (FastAMP master mix includes 2 µl 10Xbuffer, 4 µl 2mM dNTPs, 3.2 µl 5M betaine, 1.2 µl 100 mM $MgSO_4$, 1.6 µl primer mixture), 1 µl *Bst* DNA polymerase enzyme, 1 µl fluorescent detection reagent and 6

µl of extracted DNA samples. Amplification of reaction mixture was performed in the heating blocks at 65°C for 60 minutes, then examined directly by visual observation. The LAMP assay was considered “positive” if the color of the reaction mixture changed from orange to green or fluorescence was directly observed with the naked eyes. The test was considered “negative” if the color of the mixture remained unchanged. For quality control, positive control (test tube with M. *tuberculosis* genetic materials) and negative control (test tube without M. *tuberculosis* genetic materials) were included in all runs.

Statistical Analysis

We used Fisher’s exact probability test for comparison of differences in independent proportions and Student’s t-test for two independent means. The sensitivity, specificity, positive predictive values (PPV), negative predictive values (NPV), and positive and negative likelihood ratios were calculated and reported with its 95% confidence interval. The 95% confidence interval were estimated using the Clopper Pearson binomial exact method. The comparison of sensitivity, specificity, and overall test accuracy between the LAMP test and smear microscopy methods was performed with McNemar’s exact probability test. Pairwise testing to compare the specificity between the LAMP test and the smear microscopy methods was not performed as the specificity of the latter was affected by incorporation bias and would not be comparable to the in-house LAMP. The agreement of the LAMP test with smear microscopy methods was analyzed with Kappa’s statistics and Spearman’s rank correlation. P-values of less than 0.05 were considered statistically significant. All statistical analyses were done using Stata version 16 (StataCorp, Texas).

Results

A total of 120 patients to be evaluated for TB were consecutively included from April to August 2016. Three patients with two contaminated cultures, two patients who subsequently were detected as previously documented TB cases, and eight patients who had smear-positive and culture-positive results were excluded from the analysis; only 107 patients remained in the study (Fig. 1). Most of the included patients were male (60% vs. 40%) with a mean age of 47 years old. Fifty (46.7%) were culture-positive TB patients and 57 (53.3%) were culture-negative patients. The baseline demographic data between culture-positive and culture-negative patients were comparable. For clinical characteristics, the presence of cavitary lesions on chest radiographs and the character of collected sputum was found to be significantly different (Table 1). Culture-positive TB patients had higher proportion of cavitary lesions (14.0% vs. 1.8%, p=0.024) and mucous sputum specimen (52.0% vs 24.6%, p=0.005) than patients with negative TB culture.

Table 1. Demographic and clinical characteristics of the patients by TB culture status

Characteristics	TB Culture Positive (S+ or S-, C+)	TB Culture Negative (S-, C-)	P-Value
	n=50 (46.7%)	n=57 (53.3%)	
Gender			
Male	30 (60.0)	36 (63.2)	0.842
Female	20 (40.0)	21 (36.8)	
Nationality			
Thai	28 (56.0)	21 (36.8)	0.054
Non-Thai	22 (44.0)	36 (63.2)	
Age (year, mean±SD)	48.7±17.4	45.8±18.7	0.408
Chest radiographs			
Without cavitary lesions	43 (86.0)	56 (98.2)	0.024
With cavitary lesions	7 (14.0)	1 (1.8)	
Character of sputum			
Salivary	24(48.0)	43 (75.4)	0.005
Mucous	26 (52.0)	14 (24.6)	

Abbreviations: TB, tuberculosis; C, culture (+ positive or – negative); S, smear microscopy (+ positive or – negative); SD, standard deviation.

Fig. 1. Study flow diagram of patient enrollment and results of index and reference test based on culture result

The overall sensitivity of the LAMP test was 82.0% (95%CI 68.6-91.4), whereas the sensitivity in smear-positive, culture-positive patients and smear-negative, culture-positive was 90.9% (95%CI 78.3-97.5) and 16.7% (95%CI 0.4-64.1), respectively. The overall sensitivity of both the AFB and the fluorescence stain was slightly higher than that of the LAMP test; however, the differences were non-significant (Table 2). The specificity, positive predictive value, and negative predictive value of LAMP test was 94.7% (95%CI 85.4-98.9), 93.2% (95%CI 81.3-98.6), and 85.7% (95%CI 74.6-93.3), respectively. The positive and negative likelihood ratios of the LAMP test was 15.6 (95%CI 4.47-82.12) and 0.19 (95%CI 0.08-0.44), respectively. Even though the accuracy measures for the diagnosis of TB cases were shown to vary across different test methods (LAMP test, AFB stain, and fluorescence stain), the differences were without statistical significance (Table 2).

LAMP test results were highly correlated with those of AFB and fluorescence stain (Spearman’s rho 0.85, $p<0.001$) in the diagnosis of culture-positive TB cases (Table 3). The in-house LAMP also showed substantial to almost perfect agreement with both microscopy methods in the diagnosis of culture-positive cases (Kappa 0.85, 95%CI 0.74,0.95) (Table 3).

Table 2. Diagnostic accuracy of the in-house LAMP test, AFB stain, and Fluorescence stain.

Method	Sensitivity% (95% CI), no. corrects			Specificity% (95%CI), no. corrects	Accu[illegible]% (95%CI), no. corrects (n=107)	PPV% (95%CI)	NPV (95%CI)	LR+ (95%CI)	LR- (95%CI)
	S+, C+ (n=44)	S-, C+ (n=6)	Any S, C+ (n=50)	S-, C- (n=57)					
LAMP	90.9 (78.3,97.5), N=40	16.7 (0.4,64.1), n=1	82.0 (68.6,91.4), n=41	94.7 (85.4,98.9), n=54	88.8 (81.2,94.1), n=95	93.2 (81.3,98.6)	85.7 (74.6,93.3)	15.6 (4.5,82.1)	0.2 (0.1,0.4)
AFB stain	-	-	88.0 (75.7,95.5), n=44	100.0 (93.7,100.0), n=57	94.4 (88.2,97.9), n=101	100.0 (93.7,100.0)	90.5 (80.4,96.4)	-	-
Fluorescence stain	-	-	88.0 (75.7,95.5), n=44	100.0 (93.7,100.0), n=57	94.4 (88.2,97.9), n=101	100.0 (93.7,100.0)	90.5 (80.4,96.4)	-	-
LAMP test vs. AFB stain			P=0.375*	P=0.250*	P=1.000*				
LAMP test vs. Fluorescence stain			P=0.375*	P=0.250*	P=1.000*				

*P-values from McNemar's Exact probability test

Abbreviations: AFB, acid fast bacilli; C, culture (+ positive or – negative); CI, confidence interval; LAMP, loop-mediated isothermal amplification; LR+, positive likelihood ratio; LR-, negative likelihood ratio; no. correct, number correctly identified; NPV, negative predictive value; PPV, positive predictive value; S, smear microscopy (+ positive or – negative).

Table 3. Diagnostic agreement and correlation between the in-house LAMP test and AFB stain-fluorescence stain.

LAMP Test	AFB Stain & Fluorescence stain		
	Positive	Negative	Total
Positive	40	4	44
Negative	4	59	63
Total	44	63	107
Agreement (%)	92.5%		
Kappa (95%CI, p-value)	0.85 (0.74, 0.95, p<0.001)		
Spearman's rho (p-value)	0.85 (p<0.001)		

Abbreviations: LAMP, loop-mediated isothermal amplification; CI, confidence interval.

Discussion

This study had demonstrated the pragmatic diagnostic performance of the in-house LAMP assay in a remote hospital of a high TB burden country. It was revealed that the overall sensitivity of the in-house LAMP in our study was lower than the numbers reported in the majority of the previous in-house LAMP studies. Nonetheless, the specificity was comparable to other figures reported in the literature. In comparison to microscopy methods, the AFB and fluorescence stain, the in-house LAMP was found to be inferior in terms of overall sensitivity (82.0% vs. 88.0%, p=0.375) and accuracy (88.8% vs. 94.4%, p=1.000); however, the comparative statistical test revealed no[illegible]gnificant results. Based on the result of our study, we suggest that the in-house LAMP should not be a substitute to conventional smear methods, but should be done in parallel, which would result in a higher sensitivity with fewer false-negative TB cases.

In this study, the sensitivity of the in-house LAMP test was 82.0% (95%CI 68.6-91.4) in culture-positive TB patients, respectively. In the past, several studies had reported a higher sensitivity of the in-house LAMP test, which ranges from 90.0 to 100.0% [11,15,19–24]. Most of these studies were either University hospitals, TB-specialized centers or hospitals, or national TB-specialized laboratories, which were generally equipped with highly-trained personnel and adequate infrastructural supports. The overall sensitivity of our in-house LAMP was consistent with two previous studies from India and Zambia, which was 79.5% (95%CI 64.0-89.0) and 81.4% (95%CI 71.6-89.0), respectively [12,25]. Although both studies were performed in University hospitals, the LAMP procedures were modified to suit local conditions, and sputum processing and DNA extraction was done with commercial kits. The higher sensitivity of the acid-fast stain and the fluorescence stain in our study could be explained by the high prevalence of TB, the absence of HIV patients or less number of

patients with paucibacillary sputum, and the availability of skilled technicians [12,26–28]. Besides, specimen decontamination with concentrated NaOH decreases the amount of viable genetic materials for amplification, which could reduce the sensitivity of both the LAMP test and TB cultures. A lower concentration of NaOH (1-1.5%) or NaOH free methods during sample decontamination may be suggested [12,29]. The sensitivity of the LAMP test in smear-negative specimens could not be accurately estimated in this study as there were too few smear-negative, culture-positive patients.

The overall specificity of the LAMP test was 94.7% (95%CI 85.4-98.9) for non-TB patients, respectiv[illegible] This was in concordance with a recent meta-analysis, which reported pooled specificity of the in-ho[illegible]LAMP at 91.8% (95%CI 86.4-95.1) [14]. However, it was concluded that the specificity of the in-house assays was lower than that of the Loopamp commercial kit, which was reported at 96.5% (95%CI 94.7-97.7). A false positive LAMP result in smear-positive cases was frequently encountered in routine practice, which could be explained by multiple factors such as higher temperature, higher humidity, suboptimal reagents volume, and crossover contamination [14,30]. For in-house LAMP, an extensive laboratory technician training and continuous quality assessment should be conducted to lessen the risk of false-positive results. However, other potential factors might still account for the low specificity, such as temperature controls and volume of reaction used. For temperature, only available water bath was applied for temperature controls during LAMP procedures instead of a more stable dry heating block. A recent study suggested a high reaction volume of 30-35 µl due to the risk of self-priming in concentrated reagents [30].

Currently, the WHO only suppo[illegible] the use of two rapid molecular tests for the diagnosis of pulmonary TB, which were Xpert MTB/RIF and the commercialized TB-LAMP assay [9].

According to previous studies, both had shown comparable performance in smear-positive samples, but higher sensitivity was shown in Xpert MTB/RIF than in the LAMP test [6,25]. Xpert MTB/RIF has been endorsed for use in the diagnosis of TB in many countries, including Thailand [4,31]. Nonetheless, Xpert MTB/RIF might not be suitable in peripheral regions with poor infrastructure as the instrument requires a stable electricity supply and an appropriate environment. The device also requires high continuous maintenance costs leading to a relatively high cost per test compared to the LAMP test. The LAMP test is readily available and can be done in any resource-poor settings with regular infrastructure and technicians with adequate training. In Thailand, only a portion of patients, not including foreigners and ethnic minorities, could reimburse the cost for Xpert MTB/RIF due to the regulation stated by The National Health Security Office (NHSO). To effectively prevent the spread of TB, all patients to be evaluated for TB should have equal access to high-quality diagnostic tools. Therefore, smear microscopy and the LAMP test may be more applicable in terms of accessibility and affordability, especially in the distant areas and the borderlands.

However, there may be some limitations to this study. First, the study size might not be powered enough to confirm the statistical insignific[illegible]e of the between-test comparison. Second, no patients with HIV infection were included during the study period, as HIV status could be influential to the diagnostic performance of both the smear microscopy and the LAMP test, especially in areas with a high prevalence of TB-HIV coinfection. Third, this study had a higher proportion of salivary sputum than mucous sputum. This could affect the diagnostic performance of both the index and the reference test [32]. The percentage of culture-positive TB cases was lower in salivary samples than in mucous samples (35.8% vs. 65.0%, p=0.005). Both the quality and quantity of sputum specimens were associated with the positivity of smear, molecular testing methods (Xpert MTB/RIF and PCR), and TB

culture [33,34]. Thus, it was possible that some patients with pulmonary TB might be classified as smear-negative, LAMP-negative, or even culture-negative cases. No previous study had officially addressed the effect of sputum quality on the LAMP test. Moreover, the character of sputum specimens was rarely reported. Interestingly, it was revealed from our data that the proportion of smear-positive, LAMP-positive results was also significantly lower in salivary sputum than in mucous sputum (31.3% vs. 57.5%, p=0.009 and 29.9% vs. 60.0%, p=0.003, respectively). Therefore, the sensitivity and accuracy of all tests, including LAMP, might be underestimated. Previous studies reported that by improving the sputum quality, TB diagnostic yield increased [35,36]. Thus, high-quality sputum collection must be encouraged both in practice and studies.

Finally, the use of routine TB culture as a reference standard might be inadequate, as some TB patients could be classified as not having TB [6]. With a higher quality reference standard, the sensitivity of the in-house LAMP should be increased when a portion of three remaining false-positive cases was re-classified as true-positive cases. Different culture media and techniques could be used in composite to achieve different performance characteristics [37]. In our study, two different culture techniques, L-J and MGIT, were used to increase the diagnostic rate of TB[38]. We also applied a strict diagnostic definition in calculating specificity by considering only patients with smear-negative and culture-negative results[39].

Conclusions

In conclusion, the LAMP test is a practical and affordable nucleic amplification technique for the diagnosis of pulmonary TB, which should be implemented in resource-limiting settings where Xpert MTB/RIF is unavailable. The diagnostic accuracy of the in-hose LAMP was similar to previous studies for specificity. Better sputum processing and DNA extraction

method should be identified to improve the test sensitivity. The overall accuracy of the in-house LAMP test was comparable to that of conventional microscopy and fluorescence microscopy with minimal inferiority in terms of sensitivity. Therefore, a parallel examination of both smear microscopy and the in-house LAMP test is suggested to minimize the risk of false-negative results, especially in an endemic area.

Acknowledgements

The authors wish to acknowledge the contributions of all the medical and nursing staff of the TB clinic at Maesot hospital for their help in data collection, and all relevant personnel of The National Institute of Health, Department of Medical Science, The Ministry of Public Health for their technical advice and support.

References

1. World Health Organization. Global Tuberculosis Report 2018. World Health Organization; 2018.

2. Uplekar M, Weil D, Lonnroth K, Jaramillo E, Lienhardt C, Dias HM, et al. WHO's new end TB strategy. Lancet Lond Engl. 2015;385: 1799–1801. doi:10.1016/S0140-6736(15)60570-0

3. Parsons LM, Somoskövi Á, Gutierrez C, Lee E, Paramasivan CN, Abimiku A, et al. Laboratory Diagnosis of Tuberculosis in Resource-Poor Countries: Challenges and Opportunities. Clin Microbiol Rev. 2011;24: 314. doi:10.1128/CMR.00059-10

4. Kim C-K, Cho EA, Shin DM, Choi SW, Shin SY. Comparative Evaluation of the Loop-Mediated Isothermal Amplification Assay for Detecting Pulmonary Tuberculosis. Ann Lab Med. 2018;38: 119–124. doi:10.3343/alm.2018.38.2.119

5. Eddabra R, Ait Benhassou H. Rapid molecular assays for detection of tuberculosis. Pneumonia. 2018;10. doi:10.1186/s41479-018-0049-2

6. Shete PB, Farr K, Strnad L, Gray CM, Cattamanchi A. Diagnostic accuracy of TB-LAMP for pulmonary tuberculosis: a systematic review and meta-analysis. BMC Infect Dis. 2019;19: 268. doi:10.1186/s12879-019-3881-y

7. Notomi T, Okayama H, Masubuchi H, Yonekawa T, Watanabe K, Amino N, et al. Loop-mediated isothermal amplification of DNA. Nucleic Acids Res. 2000;28: e63.

8. Yan L, Xiao H, Zhang Q. Systematic review: Comparison of Xpert MTB/RIF, LAMP and SAT methods for the diagnosis of pulmonary tuberculosis. Tuberc Edinb Scotl. 2016;96: 75–86. doi:10.1016/j.tube.2015.11.005

9. The Use of Loop-Mediated Isothermal Amplification (TB-LAMP) for the Diagnosis of Pulmonary Tuberculosis: Policy Guidance. Geneva: World Health Organization; 2016. Available: http://www.ncbi.nlm.nih.gov/books/NBK384520/

10. Sohn H, Puri L, Nguyen NAT, Van't Hoog AH, Nguyen VAT, Nliwasa M, et al. Cost and affordability analysis of TB-LAMP and Xpert MTB/RIF assays as routine diagnostic tests in peripheral laboratories in Malawi and Vietnam. J Glob Health Sci. 2019;1: e22. doi:10.35500/jghs.2019.1.e22

11. Pandey BD, Poudel A, Yoda T, Tamaru A, Oda N, Fukushima Y, et al. Development of an in-house loop-mediated isothermal amplification (LAMP) assay for detection of Mycobacterium tuberculosis and evaluation in sputum samples of Nepalese patients. J Med Microbiol. 2008;57: 439–443. doi:10.1099/jmm.0.47499-0

12. George G, Mony P, Kenneth J. Comparison of the Efficacies of Loop-Mediated Isothermal Amplification, Fluorescence Smear Microscopy and Culture for the Diagnosis of Tuberculosis. PLoS ONE. 2011;6. doi:10.1371/journal.pone.0021007

13. Rudeeaneksin J, Bunchoo S, Srisungngam S, Sawanpanyalert P, Chamnangrom S, Kamolwat A, et al. Rapid identification of Mycobacterium tuberculosis in BACTEC MGIT960 cultures by in-house loop-medicated isothermal amplification. Jpn J Infect Dis. 2012;65: 306–311. doi:10.7883/yoken.65.306

14. Nagai K, Horita N, Yamamoto M, Tsukahara T, Nagakura H, Tashiro K, et al. Diagnostic test accuracy of loop-mediated isothermal amplification assay for Mycobacterium tuberculosis: systematic review and meta-analysis. Sci Rep. 2016;6: 39090. doi:10.1038/srep39090

15. Phetsuksiri B, Rudeeaneksin J, Srisungngam S, Bunchoo S, Klayut W, Nakajima C, et al. Comparison of Loop-Mediated Isothermal Amplification, Microscopy, Culture, and PCR for Diagnosis of Pulmonary Tuberculosis. Jpn J Infect Dis. 2020;advpub. doi:10.7883/yoken.JJID.2019.335

16. Kent PT. Public Health Mycobacteriology: A Guide for the Level III Laboratory. U.S. Department of Health and Human Services, Public Health Service, Centers for Disease Control; 1985.

17. Phetsuksiri B, Rudeeaneksin J, Srisungngam S, Bunchoo S, Roienthong D, Mukai T, et al. Applicability of in-house loop-mediated isothermal amplification for rapid identification of Mycobacterium tuberculosis complex grown on solid media. Jpn J Infect Dis. 2013;66: 249–251. doi:10.7883/yoken.66.249

18. Rudeeaneksin J. Rapid Detection of Mycobacterium Tuberculosis Complex in Sputum and in Culture Growth by LAMP Test. J Health Sci - วารสารวิชาการสาธารณสุข. 2017;19: 300–310.

19. Rafati A, Gill P. Microfluidic method for rapid turbidimetric detection of the DNA of Mycobacterium tuberculosis using loop-mediated isothermal amplification in capillary tubes. Microchim Acta. 2015;182: 523–530. doi:10.1007/s00604-014-1354-y

20. Poudel A, Pandey BD, Lekhak B, Rijal B, Sapkota BR, Suzuki Y. Clinical profiling and use of loop-mediated isothermal amplification assay for rapid detection of Mycobacterium tuberculosis from sputum. Kathmandu Univ Med J KUMJ. 2009;7: 109–114. doi:10.3126/kumj.v7i2.2701

21. Sethi SK, Singh S, Dhatwalia SK, Yadav R, Mewara A, Singh M, et al. Evaluation of In- House Loop- Mediated Isothermal Amplification (LAMP) Assay for Rapid Diagnosis of M. tuberculosis in Pulmonary Specimens. J Clin Lab Anal. 2013;27: 272–276. doi:10.1002/jcla.21596

22. Li Y, Shi L, Pan A, Cao W, Chen X, Meng H, et al. Evaluation of real-time loop-mediated isothermal amplification (RealAmp) for rapid detection of Mycobacterium tuberculosis from sputum samples. J Microbiol Methods. 2014;104: 55–58. doi:10.1016/j.mimet.2014.06.011

23. Lee M-F, Chen Y-H, Peng C-F. Evaluation of reverse transcription loop-mediated isothermal amplification in conjunction with ELISA-hybridization assay for molecular detection of

Mycobacterium tuberculosis. J Microbiol Methods. 2009;76: 174–180. doi:10.1016/j.mimet.2008.10.005

24. Kohan L, Shahhosseiny MH, Razavi MR, Parivar K, Moslemi E, Werngren J. Evaluation of loop mediated isothermal amplification for diagnosis of *Mycobacterium tuberculosis* complex in clinical samples. Afr J Biotechnol. 2011;10: 5096–5101. doi:10.4314/ajb.v10i26.

25. Habeenzu C, Nakajima C, Solo E, Bwalya P, Kajino K, Miller M, et al. Evaluation of in-house loop-mediated isothermal amplification for tuberculosis diagnosis compared with Xpert MTB/RIF. J Infect Dev Ctries. 2017;11: 440–444. doi:10.3855/jidc.7730

26. Pandey BD, Poudel A, Yoda T, Tamaru A, Oda N, Fukushima Y, et al. Development of an in-house loop-mediated isothermal amplification (LAMP) assay for detection of Mycobacterium tuberculosis and evaluation in sputum samples of Nepalese patients. J Med Microbiol. 2008;57: 439–443. doi:10.1099/jmm.0.47499-0

27. Pathak D, Chakravorty S, Hanif M, Tyagi JS. Lysis of tubercle bacilli in fresh and stored sputum specimens: implications for diagnosing tuberculosis in stored and paucibacillary specimens by PCR. BMC Microbiol. 2007;7: 83. doi:10.1186/1471-2180-7-83

28. Shea YR, Davis JL, Huang L, Kovacs JA, Masur H, Mulindwa F, et al. High Sensitivity and Specificity of Acid-Fast Microscopy for Diagnosis of Pulmonary Tuberculosis in an African Population with a High Prevalence of Human Immunodeficiency Virus. J Clin Microbiol. 2009;47: 1553–1555. doi:10.1128/JCM.00348-09

29. Pham TH, Peter J, Mello FCQ, Parraga T, Lan NTN, Nabeta P, et al. Performance of the TB-LAMP diagnostic assay in reference laboratories: Results from a multicentre study. Int J Infect Dis IJID Off Publ Int Soc Infect Dis. 2018;68: 44–49. doi:10.1016/j.ijid.2018.01.005

30. Gray CM, Katamba A, Narang P, Giraldo J, Zamudio C, Joloba M, et al. Feasibility and Operational Performance of Tuberculosis Detection by Loop-Mediated Isothermal

Amplification Platform in Decentralized Settings: Results from a Multicenter Study. J Clin Microbiol. 2016;54: 1984–1991. doi:10.1128/JCM.03036-15

31. Pai M, Flores LL, Pai N, Hubbard A, Riley LW, Colford JM. Diagnostic accuracy of nucleic acid amplification tests for tuberculous meningitis: a systematic review and meta-analysis. Lancet Infect Dis. 2003;3: 633–643. doi:10.1016/s1473-3099(03)00772-2

32. Shi J, Dong W, Ma Y, Liang Q, Shang Y, Wang F, et al. GeneXpert MTB/RIF Outperforms Mycobacterial Culture in Detecting Mycobacterium tuberculosis from Salivary Sputum. In: BioMed Research International [Internet]. 2018 [cited 12 Dec 2019]. Available: https://www.hindawi.com/journals/bmri/2018/1514381/

33. Ho J, Marks GB, Fox GJ. The impact of sputum quality on tuberculosis diagnosis: a systematic review. Int J Tuberc Lung Dis Off J Int Union Tuberc Lung Dis. 2015;19: 537–544. doi:10.5588/ijtld.14.0798

34. Yoon SH, Lee NK, Yim JJ. Impact of sputum gross appearance and volume on smear positivity of pulmonary tuberculosis: a prospective cohort study. BMC Infect Dis. 2012;12: 172. doi:10.1186/1471-2334-12-172

35. Hirooka T, Higuchi T, Tanaka N, Ogura T. [The value of proper sputum collection instruction in detection of acid-fast bacillus]. Kekkaku. 2004;79: 33–37.

36. Sicsú AN, Salem JI, Fujimoto LBM, Gonzales RIC, Cardoso M do S de L, Palha PF. Educational intervention for collecting sputum for tuberculosis: a quasi-experimental study 1. Rev Lat Am Enfermagem. 2016;24. doi:10.1590/1518-8345.0363.2703

37. Cudahy P, Shenoi S. Diagnostics for pulmonary tuberculosis. Postgrad Med J. 2016;92: 187–193. doi:10.1136/postgradmedj-2015-133278

38. Kadioglu EE, Ucar EY, Araz O, Aktas E, Saglam L. A Comparison of Two Different Culture Methods for Use in the Diagnosis of Pulmonary Tuberculosis. Eurasian J Med. 2014;46: 74–77. doi:10.5152/eajm.2014.19

39. Kaku T, Minamoto F, D’Meza R, Morose W, Boncy J, Bijou J, et al. Accuracy of LAMP-TB Method for Diagnosing Tuberculosis in Haiti. Jpn J Infect Dis. 2016;69: 488–492. doi:10.7883/yoken.JJID.2015.519

Supporting information

S1 Table. Review on diagnostic accuracy of in-house LAMP assays for diagnosis of pulmonary tuberculosis (DOCX)

S2 Table. LAMP minimal dataset (CSV)

Figure 1

Click here to access/download;Figure;Figure 1.png

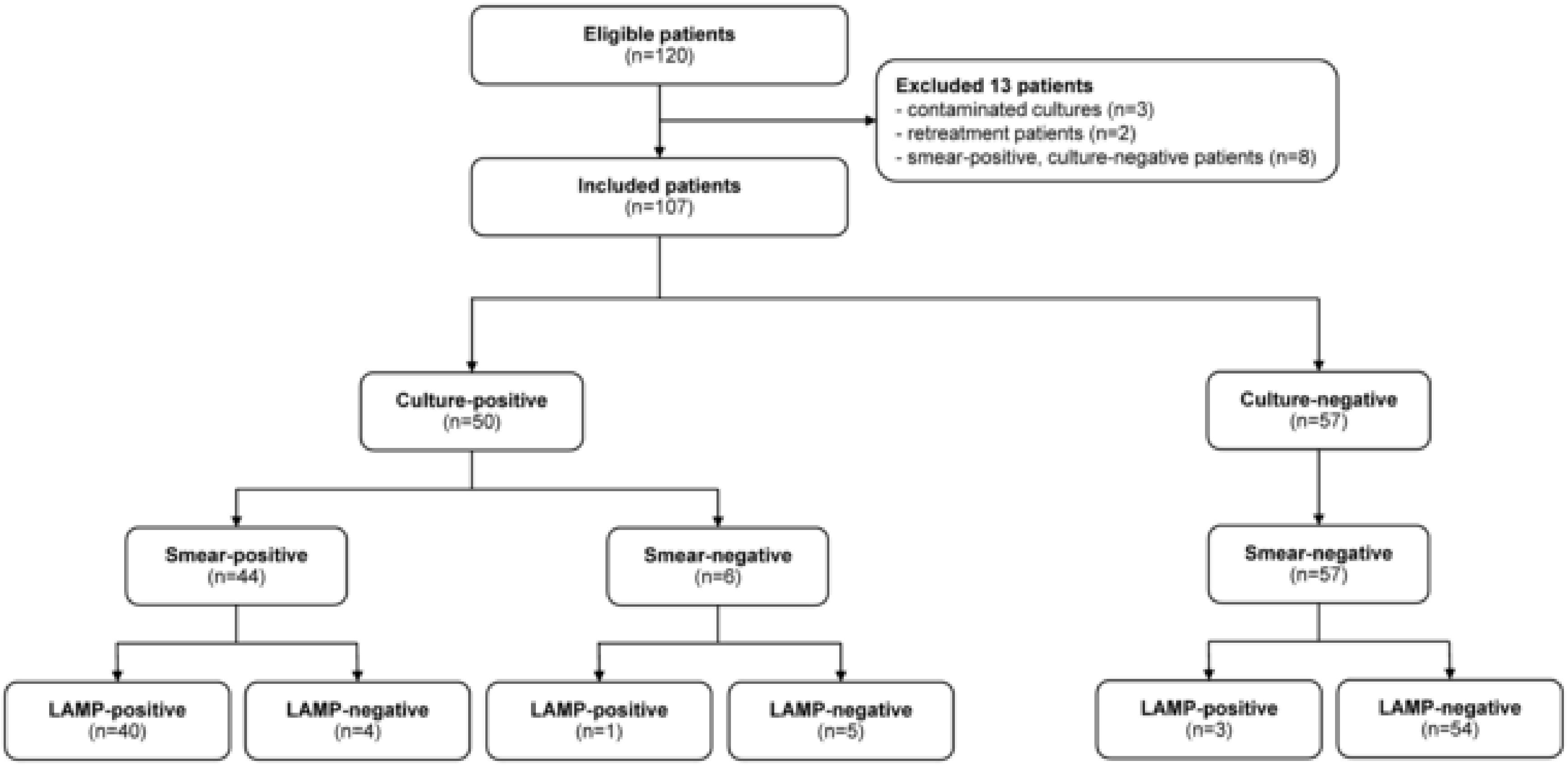

S2 LAMP dataset

Click here to access/download
Supporting Information
S2 LAMP dataset.csv

S1 Review table

Click here to access/download
Supporting Information
Table S1.docx

Pragmatic accuracy of in-house ~~of~~ loop-mediated isothermal amplification (LAMP) for diagnosis of pulmonary tuberculosis in a Thai community hospital

Short title: Diagnostic accuracy of in-house LAMP for pulmonary TB

Sarawut Toonkomdang[1], Phichayut Phinyo[2,3*], Benjawan Phetsuksiri[4], Jayanton Patumanond[3], Janisara Rudeeaneksin[4¶], Wiphat Klayut[4¶]

[1] Department of Medical Technology, Maesot General Hospital, Tak, Thailand.

[2]Department of Family Medicine, Faculty of Medicine, Chiang Mai University, Chiang Mai, Thailand.

[3] Center for Clinical Epidemiology and Clinical Statistics, Faculty of Medicine, Chiang Mai University, Chiang Mai, Thailand.

[4] National Institute of Health, Department of Medical Sciences, Ministry of Public Health, Nonthaburi, Thailand.

*Corresponding author:

E-mail: phichayutphinyo@gmail.com (PP)

¶These authors contributed equally to this work.

Formatted: Line spacing: Double

Formatted: Line spacing: Double

Formatted: No underline

Formatted: Line spacing: Double

Abstract

Background: To improve the quality of diagnosing pulmonary tuberculosis (TB), WHO recommends the use of rapid molecular testing as an alternative to conventional microscopic methods. Loop-mediated isothermal amplification assay (LAMP test) is a practical and ~~recently developed~~cost-effective nucleic amplification technique. ~~with proven diagnostic performance over the past decades. However, most of the results were validated within large centers with highly skilled personnel and adequate infrastructure.~~ We evaluated the pragmatic accuracy of ~~the~~ the in-house LAMP assay for the diagnosis of TB in a remote health care setting where an advanced rapid molecular test is not available.

Methods: ~~Diagnostic accuracy research using a cross-sectional design~~A prospective diagnostic accuracy study was conducted. ~~Clinically suspected TB patients~~Patients with clinical symptoms suggestive of TB were consecutively ~~included~~ enrolled from April to August 2016. Sputum samples were collected from each patient and were sent for microscopic examination (both acid-fast stain and fluorescence stain), ~~-~~in-house LAMP test, and TB culture~~, and LAMP test~~.

Results: One hundred and ~~fifteen~~ seven ~~TB suspects~~patients with TB symptoms were used in the final analysis. This included 50 (~~43.5~~46.7%) culture-positive TB patients and 57~~65~~ (53.3~~6.5~~%) culture-negative patients. ~~The sensitivity, specificity, positive predictive value, and negative predictive value of the LAMP test compared to the reference TB culture were 82.0% (68.6-91.4), 84.6% (73.5-92.4), 80.4% (66.9-90.2), and 85.9% (75.0-93.4), respectively.~~The overall sensitivity of the in-house LAMP based on culture positivity was 88.8% (95%CI 81.2,94.1). The sensitivity was 90.9% (95%CI 78.3,97.5) for smear-positive, culture-positive patients, and was 16.7% (95%CI 0.4,64.1) for smear-negative, culture-

positive patients. The overall ~~diagnostic performan~~sensitivity and accuracy~~ce~~ of the in-house LAMP test compared to ~~direct microscopic examination and fluorescence microscop~~smear microscopy methods~~y~~ were not significantly different (p=0.375 and p=1.000, respectively). The specificity of the in-house LAMP based on non-TB patients (smear-negative, culture-negative) was 94.7% (95%CI 85.4,98.9).~~.~~

Conclusions: The diagnostic accuracy of ~~the~~ the in-house LAMP test in a community hospital was comparable to other previous reports in terms of specificity. The sensitivity of the in-house assay could be improved with better sputum processing and DNA extraction method. ~~comparable to the conventional smear microscopy examination for the diagnosis of TB in a remote hospital of high TB burden country. Serial testing of both tests may be suggested to improve the overall accuracy of TB diagnosis.~~

Keywords: Pulmonary Tuberculosis, in-house LAMP, Diagnosis, Sensitivity, Specificity

Introduction

Tuberculosis (TB), an airborne communicable disease, has long been considered ~~as~~ a significant threat to global public health. According to The World Health Organization (WHO), 10 million people were newly infected with TB in 2018. Although the incidence and prevalence of TB vary greatly across the globe, 87% of total cases resided within 30 countries with high TB burden, including Thailand, where the incidence rate was 153 cases per 100,000 population in 2018 [1]. Early diagnosis and timely treatment is an essential component of The End TB Strategy endorsed by the WHO, aiming to end the global TB epidemic by the year 2035 [2]. However, tuberculosis is still underdiagnosed and undertreated, especially in resource-limiting countries due to the lack of highly sensitive and specific diagnostic tools which are usually expensive and require adequate infrastructure [1,3]. Novel diagnostic methods with enough simplicity and cost-effectiveness are therefore necessary to improve accurate identification of tuberculosis patients in these particular settings [3,4]~~[4,5]~~.

Molecular testing methods such as polymerase chain reaction (PCR) or Xpert MTB/RIF have been widely acknowledged as alternative tools for the diagnosis of tuberculosis patients [3,5]~~[5,6]~~. These nucleic amplification techniques were known for yielding rapid and accurate TB diagnosis, which would ~~clearly~~ overcome the limitations of classical methods, insensitivity for smear microscopy, and lengthy incubation period for TB culture. However, several obstacles remain for the application of these molecular tests as point-of-care testing in community settings because of their complexity in executions and substantial requirements for financial and personnel resources [3,6]~~[3,7]~~. Loop-mediated isothermal amplification (LAMP) assay is another recently developed nucleic acid amplification technique. Unlike PCR, where the amplification of DNA fragment occurs in temperature-dependent steps, the reaction of LAMP assay functions in isothermal~~,~~ or constant temperature~~,~~ conditions

Field Code Changed

Field Code Changed

Field Code Changed

Field Code Changed
Formatted: Font: (Default) Times New Roman, 12 pt
Formatted: Font: 11 pt, Font color: Auto

Formatted: Font: (Default) Times New Roman, 12 pt
Formatted: Font: 11 pt, Font color: Auto
Field Code Changed

Field Code Changed
Formatted: Font: (Default) Times New Roman, 12 pt
Formatted: Font: 11 pt, Font color: Auto

[7,8][8,9]. LAMP assay has a low cost per test, does not required advanced technological facilities, and can be routinely practiced in general hospital laboratories [3]. In 2016, WHO suggested the use of commercial TB-LAMP assay (Eiken Chemical Co., Tokyo, Japan) for theas a replacement for smear microscopy for the diagnosis of pulmonary tuberculosisTB in patients with symptoms suggestive of TB [9][10]. TB-LAMP assay has a low cost per test, does not required advanced technological facilities, and can be routinely practiced in general hospital laboratories [6,10].

As health care financial resources are usually limited in countries with high TB prevalence, setting up an infrastructure to support the commercial TB-LAMP could still be unattainable. A more affordable in-house LAMP was developed in 2008 [11]. The main advantage of the in-house assay was that it could be implicated on the readily-available infrastructure of any laboratory, even in the decentralized one. However, it did require extra-training and skill of technicians to process the clinical specimens. a simple and affordable molecular test would be suitable for achieving accurate TB diagnosis. In the past decades, several clinical studies and meta-analyses had evaluated the diagnostic accuracy of the in-house LAMP test for the diagnosis of pulmonary tuberculosis [12–14][7,11-14] (S1 Table). Overall, the LAMPFrom the latest meta-analysis, the overall sensitivity and specificity of the in-house LAMP was 93.0% (95%CI 88.9-95.7) and 91.8% (95%CI 86.4-95.1), respectively [14]. One recent study in Thailand reported the sensitivity and the specificity of the in-house LAMP at 94.4% (95%CI 88.9-97.7) and 94.3% (95%CI 87.2-98.1), respectively [15]. assay revealed high diagnostic performance especially in smear-positive TB patients and had been suggested as an alternative test for TB diagnosis, especially in resource-limiting areas where advanced molecular tests (e.g. PCR and Xpert MTB/RIF) are inaccessible [1,7]. However, the LAMP procedures and types of assay used (in-house or commercialized kit) varied across studies

Formatted: Font: (Default) Times New Roman, 12 pt
Formatted: Font: 11 pt, Font color: Auto
Field Code Changed
Field Code Changed
Field Code Changed
Formatted: Font: (Default) Times New Roman, 12 pt
Formatted: Font: 11 pt, Font color: Auto
Field Code Changed
Formatted: Font: (Default) Times New Roman, 12 pt
Field Code Changed
Formatted: Font: (Default) Times New Roman, 12 pt
Field Code Changed
Field Code Changed
Formatted: Font: (Default) Times New Roman, 12 pt
Formatted: Font: (Default) Times New Roman, 12 pt
Field Code Changed
Field Code Changed
Formatted: Font: 11 pt, Font color: Auto

and yielded some discrepancies in results. MoreoverHowever, the reported accuracy could be overestimated if being assessed in qualified laboratories with highly skilled technicians and sufficient resources where molecular tests usually are available [14][14]. Therefore, this study aimed to evaluate the pragmatic accuracy of the in-house LAMP assay for the diagnosis of pulmonary tuberculosis in a peripheral community hospital of a developing country with a high TB burden.

Field Code Changed

Formatted: Font: (Default) Times New Roman

Formatted: Font: 11 pt, Font color: Auto

Materials and Methods

Ethics Statement

This study was approved by the Research Ethics Committee of Maesot General Hospital, The Ministry of Public Health (serial number 37/2015) and The Human Research Ethics Committee of Thammasat University, Faculty of Medicine (COA number 081/2016). The clinical samples used in this study were collected from all patients as routinely done. Informed consent was obtained from all patients prior to inclusion.

Setting

The study was settled in Maesot General Hospital, a large large-sized community hospital with 365 in-patient beds. The hospital is located in Maesot district in Tak (province), which shares the border with Myanmar and provides standard health care to both Thai and non-Thai patients (Burmese immigrants and ethnic minorities). According to the Health Data Center, the ministry of public health, Thailand, the incidence rate of pulmonary TB in Maesot was 351 per 100,000 in 2019. The level of health care system of the hospital is considered rural. Maesot hospital has its own reference laboratory with biosafety cabinet infrastructure, BSC class II. There are four lab technicians and one lab assistant within each working shift. Power generator (350 kW) and UPS (2.7 kW) were available in case of power outages, which was infrequent.

Study Design

This prospective diagnostic accuracy research with a population-analog cross-sectional design was conducted from April to August 2016. New patients who were clinically

~~suspected of pulmonary TB (coughing for more than two weeks with or without hemoptysis), aged more than 18 years old were consecutively invited into the study regardless of nation status.~~Adult patients aged more than 15 years old with symptoms indicative of pulmonary TB (coughing for more than two weeks with or without hemoptysis) and no history of TB were consecutively enrolled regardless of nationality status. ~~Samples with contaminated culture results or samples from patients who were previously documented as TB cases were excluded.~~Patients with previously documented TB history or patients with two contaminated or missing cultures were excluded from the study.

Methods

Formatted: Space After: 8 pt, Line spacing: Double

Formatted: Line spacing: Double

All patients were given three sealed containers for the collection of morning sputum specimens. Of all containers sent to the laboratory, only the one with seemingly adequate sputum containing both mucoid or mucopurulent characters with a sample volume of more than 3 ml, was used for the whole investigation procedures as routinely done. Specimens were sent for smear microscopy with conventional AFB acid-fast bacilli (AFB) staining with Ziehl-Neelsen technique and fluorescence acid-fast staining with Auramine O solution. Smear-positive case was defined according to WHO definitions as the presence of at least two smears of scanty grade or one or more smears of 1+ or more. A smear negative case or AFB smear-negative was conversely defined. For TB culture, the reference test, we performed both conventional culture method on L-J (Lowenstein-Jensen) medium and BBL MGIT (mycobacterial growth indicator tube) culture method.

Sputum decontamination and culture examination

For the sputum decontamination process, the collected samples and 2% N-Acetyl-L-cysteine (NALC) NaOH were poured into a 50 ml sterile centrifuge tube in an equal proportion and were subsequently mixed by vortexing for 30 seconds and left at room temperature (20-25°C) for 15 minutes. Then, the test tubes were filled with phosphate buffer saline (pH 6.8) until the volume reached the level of 50 ml. The samples were put in a high-speed refrigerated centrifuge at 3,000 g for 20 minutes. Next, the supernatants were poured off, leaving the tube with decontaminated sputum samples. Finally, a drop (1 ml) of phosphate buffer saline (pH 6.8) was used for resuspension of the specimen.

For TB culture, the reference test, we performed both conventional culture method on L-J (Lowenstein-Jensen) medium and BBL MGIT 960 (mycobacterial growth indicator tube) culture method. The cCulture media were inoculated with processed sputum specimens and incubated at 35 to 37℃ and monitored weekly for growth until 8 weeks. The sputum samples were considered as “culture-positive” if growth was detected in either of L-J or MGIT culture, regardless of the smear status. If growth was not detected in neither of the culture methods and both microscopy results were negative, the samples were considered as “culture-negative” or “non-TB patients”. Patients with smear-positive and culture-negative, which were generally considered as probable TB, were excluded from the analysis. Both smear microscopy and culture methods were performed according to the standard protocols [16][15].

In-house LAMP test

The LAMP test consists of three steps as follows: DNA extraction, isothermal amplification, and visual interpretation with fluorescence. The National Institute of Health of Thailand had developed the TB Fast Amp technique (a modified LAMP procedure) to suite local practice since 2009. The procedures were described as follow. Flexi Gene® DNA Kit (Qiagen co., USA) and Protenase K Kit (Qiagen co., USA) were used for DNA extraction [17,18][16,17]. Four primers (MTB primers, MAV primers, MIN primers, and Muniv primers) were used for the recognition of six distinct regions on the 16S ribosomal RNA gene of M. *tuberculosis*. Each single LAMP reaction includes 12 µl of TB-Fast AMP mixture (FastAMP master mix includes 2 µl 10Xbuffer, 4 µl 2mM dNTPs, 3.2 µl 5M betaine, 1.2 µl 100 mM $MgSO_4$, 1.6 µl primer mixture), 1 µl *Bst* DNA polymerase enzyme, 1 µl fluorescent detection reagent and 6 µl of extracted DNA samples. Amplification of reaction mixture was performed in the heating blocks at 65℃ for 60 minutes, then examined directly by visual observation. The LAMP assay was considered “positive” if the color of the reaction mixture changed from

Formatted: Font: (Default) Times New Roman

Field Code Changed

Formatted: Font: 11 pt

Field Code Changed

Formatted: Font: (Default) Times New Roman

Formatted: Font: 11 pt

orange to green or fluorescence was directly observed with the naked eyes. The test was considered "negative" if the color of the mixture remained unchanged. For quality control, positive control (test tube with M. *tuberculosis* genetic materials) and negative control (test tube without M. *tuberculosis* genetic materials) were included in all runs.

~~Study size estimation~~

~~Pandey et al. reported the sensitivity and specificity of in-house LAMP assay for MTB detection at 97% and 94%, respectively [18]. Based on the hypothesis that the sensitivity of the LAMP test in this study would not differ from that previously reported by more than 10%, the study size was estimated (using one-sample comparison of proportion to hypothesized value), yielding a total number of 60 culture-positive TB cases. From a retrospective review of Maesot General Hospital data, the prevalence of culture-positive TB cases was 50% of all patients who were TB suspects. A total of 120 patients were therefore planned to be included in our study.~~

Field Code Changed

Statistical Analysis

~~Frequency and percentage were used for the description of categorical data. For continuous data, visualization of data distribution was done with histogram. For normally distributed data, mean and standard deviation was reported. For non-normally distributed data, median and interquartile range was reported.~~ We used Fisher's exact probability test for comparison of differences in independent proportions and Student's t-test for two independent means. The sensitivity, specificity, positive predictive values (PPV), negative predictive values (NPV), and positive and negative likelihood ratios ~~(LHR+) of all testing methods~~ were calculated and reported with its 95% confidence interval. The 95% confidence interval were

estimated using the Clopper Pearson binomial exact method. The comparison of sensitivity, specificity, and overall test accuracy between the LAMP test and smear microscopy methods was performed with McNemar's exact probability test. Pairwise testing to compare the specificity between the LAMP test and the smear microscopy methods was not performed as the specificity of the latter was affected by incorporation bias and would not be comparable to the in-house LAMP. The agreement of the LAMP test with smear microscopy methods was analyzed with Kappa's statistics and Kappa statisticsSpearman's rank correlation. The subgroup analysis of LAMP test accuracy in smear-negative, culture positive TB patients was pre-specified. P-values of less than 0.05 were considered statistically significant. All statistical analyses were done using Stata version 16 (StataCorp, Texas).

Results

A total of 120 ~~clinically suspected cases of TB~~patients to be evaluated for TB were consecutively included from April to August 2016. Three patients with two contaminated cultures, ~~and~~ two patients who subsequently were detected as previously documented TB cases, and eight patients who had smear-positive and culture-positive results were excluded from the analysis; only ~~115~~ 107 ~~samples~~ patients remained in the study (Fig. 1). Most of the included patients were male (60% vs. 40%) with a mean age of 47 years old. Fifty (~~43~~46.7~~5~~%) were culture-positive TB patients and ~~65~~ 57 (~~56.5~~53.3%) were culture-negative patients. The baseline demographic data between culture-positive and culture-negative patients were comparable. -For clinical characteristics, ~~except for~~ the presence of cavitary lesions on chest radiographs and the character of collected sputum was found to be significantly different (Table 1). Culture-positive TB patients had higher proportion of cavitary lesions (14.0% vs. 1.~~5~~8%, p=0.~~020~~024) and mucous sputum specimen (52.0% vs 24.6%, p=0.~~003~~005) than patients with negative TB culture.

~~**Table 1. Baseline clinical characteristics of the patients by culture status**~~

~~Characteristics~~	~~TB Culture Positive~~	~~TB Culture Negative~~	~~P-Value~~
	~~n=50 (43.5%)~~	~~n=65 (56.5%)~~	
~~Gender~~			
~~Male~~	~~30(60.0)~~	~~39(60.0)~~	~~1.000~~
~~Female~~	~~20(40.0)~~	~~26(40.0)~~	
~~Nationality~~			
~~Thai~~	~~22(44.0)~~	~~38(58.5)~~	~~0.136~~
~~Non-Thai~~	~~28(56.0)~~	~~27(41.5)~~	
~~Age (year, mean±SD)~~	~~48.7±17.4~~	~~45.6±18.5~~	~~0.362~~
~~Chest radiographs~~			
~~without cavitary lesions~~	~~43(86.0)~~	~~64(98.5)~~	~~0.020~~
~~with cavitary lesions~~	~~7(14.0)~~	~~1(1.5)~~	
~~Character of sputum~~			
~~Salivary~~	~~24(48.0)~~	~~49(75.4)~~	~~0.003~~
~~Mucous~~	~~26(52.0)~~	~~16(24.6)~~	

Table 1. Demographic and clinical characteristics of the patients by TB culture status

Characteristics	TB Culture Positive	TB Culture Negative	P-Value
	(S+ or S-, C+)	(S-, C-)	
	n=50 (46.7%)	n=57 (53.3%)	
Gender			
Male	30 (60.0)	36 (63.2)	0.842
Female	20 (40.0)	21 (36.84)	
Nationality			
Thai	28 (56.0)	21 (36.8)	0.054
Non-Thai	22 (44.0)	36 (63.2)	
Age (year, mean±SD)	48.7±17.4	45.8±18.7	0.408

Formatted: Line spacing: Double
Formatted: Font: 12 pt
Formatted: Line spacing: Double
Formatted: Font: 12 pt

Chest radiographs			
W~~w~~ithout cavitary lesions	43 (86.0)	56 (98.2)	~~0.02~~0.024
W~~w~~ith cavitary lesions	7 (14.0)	1 (1.8)	
Character of sputum			
Salivary	24(48.0)	43 (75.4)	0.005
Mucous	26 (52.0)	14 (24.6)	

Abbreviations: TB, tuberculosis; C, culture (+ positive or – negative); S, smear microscopy (+ positive or – negative); SD, standard deviation.

~~Abbreviations: TB, tuberculosis; SD, standard deviation.~~

Fig. 1. Study flow diagram of patient enrollment and results of index and reference test based on ~~conventional smear microscopy~~culture result

The overall sensitivity of the LAMP test was 82.0% (95%CI 68.6-91.4), whereas the sensitivity in smear-positive, culture-positive patients and smear-negative, culture-positive was 90.9% (95%CI 78.3-97.5) and 16.7% (95%CI 0.4-64.1), respectively. The overall sensitivity of both the AFB and the fluorescence stain was slightly higher than that of the LAMP test; however, the differences were non-significant (Table 2). The ~~sensitivity,~~ specificity, positive predictive value, and negative predictive value of LAMP test ~~compared to the reference TB culture~~ was ~~were 82.0% (68.6-91.4), 84.6~~94.7% ((95%CI ~~73.5-92.4~~85.4-98.9)), ~~80.4~~93.2% ((95%CI ~~66.9-90.2~~81.3-98.6)), and ~~85.9~~85.7% ((95%CI ~~75.0-93.4~~74.6-93.3)), respectively. ~~The diagnostic accuracy of both the AFB and the fluorescence stain was slightly higher than that of the LAMP test; however, the differences were non-significant (Table 2).~~ The positive and negative likelihood ratios of the LAMP test was 15.6 (95%CI 4.47-82.12) and 0.19 (95%CI 0.08-0.44), respectively~~all tests were depicted in table 2~~. Even though the accuracy measures for the diagnosis of tuberculosis cases were shown to vary across different test methods (LAMP test, AFB stain, and fluorescence stain), the differences were without statistical significance (Table 2).

LAMP test results ~~showed substantial to almost perfect agreement~~were highly correlated with ~~both~~ those of AFB ~~(Kappa 0.82, 95%CI: 0.64-1.01, p<0.001)~~ and fluorescence stain (~~Kappa 0.84, 95%CI: 0.66-1.03, p<0.001~~Spearman's rho 0.85, p<0.001) in the diagnosis of culture-positive TB cases (Table 3). The in-house LAMP also showed substantial to almost perfect agreement with both microscopy methods in the diagnosis of culture-positive cases (Kappa 0.85, 95%CI 0.74,0.95) (Table 3).

Formatted: Line spacing: Double

Table 2. Diagnostic accuracy of LAMP test, AFB stain, Fluorescence stain, parallel and serial testing of LAMP test and AFB stain.

	TB Culture Positive (n=50)	TB Culture Negative (n=65)	TB Culture Total (n=115)	Sensitivity % (95%CI)	Specificity % (95%CI)	PPV % (95%CI)	NPV % (95%CI)	LHR+ (95%CI)
LAMP Test								
Positive	41	10	51	82.0	84.6	80.4	85.9	5.3
Negative	9	55	64	(68.6-91.4)	(73.5-92.4)	(66.9-90.2)	(75.0-93.4)	(3.0-9.6)
AFB stain								
Positive	44	7	51	88.0	89.2	86.3	90.6	8.2
Negative	6	58	64	(75.7-95.5)	(79.1-95.6)	(73.7-94.3)	(80.7-96.5)	(4.0-16.6)
Fluorescence stain								
Positive	44	8	52	88.0	87.7	84.6	90.5	7.2
Negative	6	57	63	(75.7-95.5)	(77.2-94.5)	(71.9-93.1)	(80.4-96.4)	(3.7-13.8)
Parallel testing (LAMP or AFB)								
Positive	45	11	56	90.0	83.1	80.4 (67.6-89.9)	91.5 (81.3-97.2)	5.3
Negative	5	55	59	(78.2-96.7)	(71.7-91.2)			(3.1-9.2)
Serial testing (LAMP and AFB)								
Positive	40	6	46	80.0	90.8	87.0 (73.7-95.1)	85.5 (75.0-92.8)	8.67 (3.99-18.8)
Negative	10	59	69	(66.3-90.0)	(81.0-96.5)			
P-value				0.594	0.702	0.840	0.738	

Table 2. Diagnostic accuracy of the in-house LAMP test, AFB stain, and Fluorescence stain.

Method	Sensitivity% (95% CI), no. corrects	Specificity% (95%CI), no. corrects	Accuracy% (95%CI), no. corrects	PPV% (95%CI)	NPV (95%CI)	LR+ (95%CI)	LR- (95%CI)

Formatted: Line spacing: Double
Formatted: Line spacing: Double
Formatted: Line spacing: Double
Formatted: Line spacing: Double
Formatted: Line spacing: Double
Formatted: Line spacing: Double
Formatted: Line spacing: Double
Formatted: Line spacing: Double
Formatted: Line spacing: Double
Formatted: Line spacing: Double
Formatted: Line spacing: Double
Formatted: Line spacing: Double
Formatted: Line spacing: Double
Formatted: Line spacing: Double
Formatted: Line spacing: Double
Formatted: Line spacing: Double
Formatted: Font: 12 pt
Formatted: Line spacing: Double
Formatted: Font: 12 pt
Formatted: Font: 12 pt
Formatted: Font: 12 pt

	S+, C+ (n=44)	S-, C+ (n=6)	Any S, C+ (n=50)	S-, C- (n=57)	(n=107)				
LAMP	90.9 (78.3,97.5), N=40	16.7 (0.4,64.1), n=1	82.0 (68.6,91.4), n=41	94.7 (85.4,98.9), n=54	88.8 (81.2,94.1), n=95	93.2 (81.3,98.6)	85.7 (74.6,93.3)	15.6 (4.5,82.1)	0.2 (0.1,0.4)
AFB stain	-	-	88.0 (75.7,95.5), n=44	100.0 (93.7,100.0), n=57	94.4 (88.2,97.9), n=101	100.0 (93.7,100.0)	90.5 (80.4,96.4)	-	-
Fluorescence stain	-	-	88.0 (75.7,95.5), n=44	100.0 (93.7,100.0), n=57	94.4 (88.2,97.9), n=101	100.0 (93.7,100.0)	90.5 (80.4,96.4)	-	-
LAMP test vs. AFB stain			P=0.375*	P=0.250*	P=1.000*				
LAMP test vs. Fluorescence stain			P=0.375*	P=0.250*	P=1.000*				

*P-values from McNemar's Exact probability test

Abbreviations: AFB, acid fast bacilli; C, culture (+ positive or – negative); CI, confidence interval; LAMP, loop-mediated isothermal amplification; LR+, positive likelihood ratio; LR-, negative likelihood ratio; no. correct, number correctly identified; NPV, negative predictive value; PPV, positive predictive value; S, smear microscopy (+ positive or – negative).

Formatted: Line spacing: Double

Formatted: Line spacing: Double

Formatted: Line spacing: Double

Formatted: Line spacing: Double

Formatted: Line spacing: Double

Formatted: Line spacing: Double

Table 3. Diagnostic agreement and correlation between the in-house LAMP test and AFB stain-fluorescence stain.

LAMP Test	AFB Stain & Fluorescence stain		
	Positive	Negative	Total
Positive	40	4	44
Negative	4	59	63
Total	44	63	107
Agreement (%)	92.5%		
Kappa (95%CI, p-value)	0.85 (0.74,0.95, p<0.001)		
Spearman's rho (p-value)	0.85 (p<0.001)		

Abbreviations: LAMP, loop-mediated isothermal amplification; CI, confidence interval.

~~Abbreviations: TB, tuberculosis; PPV, positive predictive value; NPV, negative predictive value; LHR+, positive likelihood ratio; CI, confidence interval; LAMP, loop-mediated isothermal amplification; AFB, acid fast bacilli.~~

~~**Table 3. Diagnostic agreement between LAMP test and AFB stain-fluorescence stain.**~~

~~LAMP Test~~	~~AFB Stain~~			~~Fluorescence stain~~		
	~~Positive~~	~~Negative~~	~~Total~~	~~Positive~~	~~Negative~~	~~Total~~
~~Positive~~	~~46~~	~~5~~	~~51~~	~~47~~	~~4~~	~~51~~
~~Negative~~	~~5~~	~~59~~	~~64~~	~~5~~	~~59~~	~~64~~
~~Total~~	~~51~~	~~64~~	~~115~~	~~52~~	~~63~~	~~115~~
~~Agreement (%)~~	~~91.3~~			~~92.2~~		
~~Kappa (95%CI)~~	~~0.82 (0.72-0.93)~~			~~0.84 (0.74-0.94)~~		
~~P-value~~	~~<0.001~~			~~<0.001~~		

~~Abbreviations: LAMP, loop-mediated isothermal amplification; CI, confidence interval.~~

Formatted: Font: 12 pt
Formatted: Font: 12 pt
Formatted: Line spacing: Double
Formatted: Font: 12 pt
Formatted: Line spacing: Double
Formatted: Line spacing: Double
Formatted: Line spacing: Double
Formatted Table
Formatted: Line spacing: Double
Formatted: Line spacing: Double
Formatted: Line spacing: Double
Formatted: Line spacing: Double
Formatted: Line spacing: Double
Formatted: Line spacing: Double
Formatted: Line spacing: Double
Formatted: Line spacing: Double
Formatted: Line spacing: Double
Formatted: Line spacing: Double
Formatted: Line spacing: Double

When parallel testing of LAMP and AFB stain was done, the sensitivity raised to 90.0% (78.2-96.7) while the specificity dropped to 83.1% (71.7-91.2). Serial testing of LAMP and AFB stain yielded higher specificity at 90.8% (81.0-96.5) with relatively lower sensitivity at 80.0% (66.3-90.0). Even though the accuracy measures for the diagnosis of tuberculosis cases were shown to vary across different test methods (LAMP test, AFB stain, fluorescence stain, parallel testing and serial testing of both LAMP and AFB stain), the differences were without statistical significance (Table 2).

Of 50 culture-positive TB cases, six were smear-negative. The sensitivity, specificity, positive predictive value, and negative predictive value of LAMP test in smear-negative, culture-positive TB patients was 16.7% (0.4-64.1), 93.1% (83.3-98.1), 20.0% (0.5-71.6), and 91.5% (81.3-97.2), respectively. In smear-positive, culture positive TB patients, the sensitivity, specificity, positive predictive value, and negative predictive value of LAMP test was 90.9% (78.3-97.5), 84.5% (74.0-92.0), 78.4% (64.7-88.7), and 93.8% (84.8-98.3), respectively.

Discussion

This study had demonstrated the pragmatic diagnostic performance performance of the in-house LAMP testassay in a remote hospital of a high TB burden country. It was revealed that the overall sensitivity of the in-house LAMP in our study was lower than the numbers reported in the majority of the previous in-house LAMP studies. Nonetheless, the specificity was comparable to other figures reported in the literature. In comparison to microscopy methods, the AFB and fluorescence stain, the in-house LAMP , which was found to be comparable inferior to that of the conventional smear microscopy and the fluorescence microscopyin terms of overall sensitivity (82.0% vs. 88.0%, p=0.375) and accuracy (88.8% vs. 94.4%, p=1.000.); however, Although the sensitivity and specificity of the LAMP test were lower than that of the acid-fast stain and the fluorescence stain, the comparative statistical test revealed non-significant results. Based on the result of our study, we suggest that the in-house LAMP should not be a substitute to conventional smear methods, but should be done in parallel, whichUsing the LAMP test and the acid-fast stain in parallel might increase the sensitivity but lower the specificity in the diagnosis of tuberculosis patients. For screening purposes, parallel testing with high sensitivity would result in a higher sensitivity

with fewer false-negative TB cases. ~~However, the relative reduction in specificity would increase the number of false positives where some patients might be subject to unnecessary treatment with serious side effects and risk of drug resistance. In the clinical context of TB diagnosis, both the LAMP test and the smear microscopy are considered as a diagnostic test which would normally be done in TB suspects with high pre-test probability [14]. Therefore, a serial test relying on both the result from the LAMP test and the acid-fast stain would be more appropriate for use as a rule-in test as it carried higher specificity and positive likelihood ratio than other methods.~~

In this study, the sensitivity of the in-house LAMP test was 82.0% (95%CI 68.6-91.4) in culture-positive ~~and 16.7% in smear-positive and smear-negative~~ TB patients, respectively. ~~Unlike most of the previous studies which reported higher sensitivity of the LAMP test compared to conventional microscopic examination [7,14], the sensitivity of the LAMP test in our study was just comparable to lower than the smear microscopy.~~ In the past, several studies had reported a higher sensitivity of the in-house LAMP test, which ranges from 90.0 to 100.0% [11,15,19–24]. Most of these studies were either University hospitals, TB-specialized centers or hospitals, or national TB-specialized laboratories, which were generally equipped with highly-trained personnel and adequate infrastructural supports. The overall sensitivity of our in-house LAMP was consistent with two previous studies from India and Zambia, which was 79.5% (95%CI 64.0-89.0) and 81.4% (95%CI 71.6-89.0), respectively [12,25]. Although both studies were performed in University hospitals, the LAMP procedures were modified to suit local conditions, and sputum processing and DNA extraction was done with commercial kits. The higher sensitivity of the acid-fast stain and the fluorescence stain in our study could be explained by the high prevalence of TB, the absence of HIV patients or

Formatted: Font: 11 pt

Field Code Changed

Formatted: English (United States)

Field Code Changed

Formatted: Font: 11 pt

Field Code Changed

Formatted: Font: (Default) Times New Roman, 12 pt

Field Code Changed

less number of patients with paucibacillary sputum, and the availability of skilled technicians [12,26–28]~~[11,18-20]~~. Besides, specimen decontamination with concentrated NaOH decreases the amount of viable genetic materials for amplification, which could reduce the sensitivity of both the LAMP test and TB cultures. A lower concentration of NaOH (1-1.5%) or NaOH free methods during sample decontamination may be suggested [12,29]~~[11,21]~~. The sensitivity of the LAMP test in smear-negative specimens could not be accurately estimated in this study as there were too few smear-negative, culture-positive patients.

The overall specificity of the LAMP test was ~~84.6% and 93.1% in smear-positive and smear-negative~~94.7% (95%CI 85.4-98.9) for non-~~-~~TB patients, respectively. ~~A positive LAMP result in a smear-positive patient is ,therefore, at high risk of false-positive, whereas a positive result in a smear-negative patient would significantly increase the probability of TB diagnosis [14].~~ This was ~~discordant~~ in concordance with a recent meta-~~analyses~~analysis, which reported ~~higher~~ pooled specificity ~~ranging from 94.0-98.1%~~of the in-house LAMP at 91.8% (95%CI 86.4-95.1) ~~for smear-positive patients and 97.7-98.6% for smear-negative patients~~ [14]~~[7,14]~~. However, it was concluded that the specificity of the in-house assays was lower than that of the Loopamp commercial kit, which was reported at 96.5% (95%CI 94.7-97.7). ~~This was due to the higher occurrence of false-positive cases in this study.~~ A false positive LAMP result in smear-positive cases was frequently encountered in routine practice, which could ~~usually~~ be explained by multiple factors such as higher temperature, higher humidity, suboptimal reagents volume, and crossover contamination [14,30]~~[14,22]~~. For in-house LAMP, a~~A~~n extensive laboratory technician training and continuous quality assessment should be conducted to lessen the risk of false-positive results. However, other potential factors might still account for the low specificity, such as temperature controls and volume of reaction used. For temperature, only available water bath was applied for

Field Code Changed

Formatted: Font: 11 pt

Field Code Changed

Formatted: Font: (Default) Times New Roman

Formatted: Font: (Default) Times New Roman

Field Code Changed

Formatted: Font: 11 pt

Field Code Changed

Formatted: Font: (Default) Times New Roman

Formatted: Font: 11 pt

Field Code Changed

Formatted: Font: (Default) Times New Roman

Formatted: Font: 11 pt

temperature controls during LAMP procedures instead of a more stable dry heating block. A recent study suggested a high reaction volume of 30-35 µl due to the risk of self-priming in concentrated reagents [30]~~[22]~~. ~~The volume of reaction in our study was lower at 20 µl which was based on the previous study of the in-house LAMP by The National Institute of Health, The Ministry of Public Health of Thailand [16].~~

~~The diagnostic accuracy of the LAMP test in smear-negative specimens was consistent with previous literature. However, the sensitivity was much lower in our study, which could result from the low number of TB cases in smear-negative samples. This information supports the use of LAMP as a rule-in test in smear-negative adult patients. In smear-positive samples, a serial test of both acid-fast stain and LAMP test would likely result in a more accurate diagnosis of TB than each in isolation. The WHO had made a conditional recommendation based on a piece of very low-quality evidence that the LAMP test may be used as an alternative test for sputum direct microscopic examination to diagnose TB suspects [10]. Based on the result of this study, we suggest that both the smear microscopic method and the LAMP test should be tested in serial to maximize the diagnostic specificity. As the LAMP test had shown different diagnostic abilities on different smear status [23], the interpretation of the LAMP test in practice should also rely on the result of smear microscopy and thus should not be done independently.~~

Currently, the WHO only supported the use of two rapid molecular tests for the diagnosis of pulmonary tuberculosis, which were Xpert MTB/RIF and the commercialized TB-LAMP ~~test~~ assay [9]~~[10]~~. According to previous studies, both had shown comparable performance in smear-positive samples, but higher sensitivity was shown in Xpert MTB/RIF than in the LAMP test [6,25]~~[7,24]~~. Xpert MTB/RIF has been endorsed for use in the diagnosis of TB in

Formatted: Font: (Default) Times New Roman
Formatted: Font: 11 pt
Field Code Changed
Field Code Changed
Formatted: Font: 11 pt
Field Code Changed
Formatted: Font: 11 pt
Field Code Changed
Formatted: Font: 11 pt
Field Code Changed
Formatted: Font: (Default) Times New Roman
Formatted: Font: 11 pt
Field Code Changed
Formatted: Font: (Default) Times New Roman
Formatted: Font: 11 pt

many countries, including Thailand [4,31]~~[4,5]~~. Nonetheless, Xpert MTB/RIF might not be suitable in peripheral regions with poor infrastructure as the instrument requires a stable electricity supply and an appropriate environment. The device also requires high continuous maintenance costs leading to a relatively high cost per test compared to the LAMP test. ~~In contrast, the~~The LAMP test is readily available and can be done in any resource-poor settings with regular infrastructure and technicians with adequate training. In Thailand, only a portion of patients, not including foreigners and ethnic minorities, could reimburse the cost for Xpert MTB/RIF due to the regulation stated by The National Health Security Office (NHSO). To effectively prevent the spread of TB, all ~~suspected~~ patients to be evaluated for TB should have equal access to high-quality diagnostic tools. Therefore, smear microscopy and the LAMP test may be more applicable in terms of accessibility and affordability, especially in the distant areas and the borderlands.

Formatted: Font: (Default) Times New Roman

Formatted: Font: 11 pt

Field Code Changed

However, there may be some limitations to this study. First, the study size ~~was~~ might not be powered enough to confirm the statistical insignificance of the between-test comparison. Second, ~~there were no new suspected TB cases with HIV infection during study recruitments~~no patients with HIV infection were included during the study period, as HIV status could be influential to the diagnostic performance of both the smear microscopy and the LAMP test, especially in areas with a high prevalence of TB-HIV coinfection. Third, this study had a higher proportion of salivary sputum than mucous sputum. This could affect the diagnostic performance of both the index and the reference test [32]. The percentage of culture-positive TB cases was lower in salivary samples than in mucous samples (35.8% vs. 65.0%, p=0.005). Both the quality and quantity of sputum specimens were associated with the positivity of smear, molecular testing methods (Xpert MTB/RIF and PCR), and TB culture [33,34]. Thus, it was possible that some patients with pulmonary TB might be

Field Code Changed

Formatted: Font: (Default) Times New Roman, 12 pt

Field Code Changed

Formatted: Font: (Default) Times New Roman, 12 pt

classified as smear-negative, LAMP-negative, or even culture-negative cases. No previous study had officially addressed the effect of sputum quality on the LAMP test. Moreover, the character of sputum specimens was rarely reported. Interestingly, it was revealed from our data that the proportion of smear-positive, LAMP-positive results was also significantly lower in salivary sputum than in mucous sputum (31.3% vs. 57.5%, p=0.009 and 29.9% vs. 60.0%, p=0.003, respectively). Therefore, the sensitivity and accuracy of all tests, including LAMP, might be underestimated. Previous studies reported that by improving the sputum quality, TB diagnostic yield increased [35,36]. Thus, high-quality sputum collection must be encouraged both in practice and studies.

~~Third, this study had a higher proportion of salivary sputum than mucous sputum. This could affect the diagnostic performance of both the index and the reference test [25]. The percentage of culture-positive TB cases was lower in salivary samples than in mucous samples (32.9% vs. 61.9%, p=0.003). Thus, it was possible that some TB patients might be classified as culture-negative or false-negative cases.~~ Finally, the use of routine TB culture as a reference standard might be inadequate, as some TB patients could be classified as not having TB [6]~~[7]~~. With a higher quality reference standard, the sensitivity of ~~TB~~ the in-house LAMP should be increased when a portion of ~~10~~ three remaining false-positive cases was re-classified as true-positive cases. Different culture media and techniques could be used in composite to achieve different performance characteristics [37]. In our study, two different culture techniques, L-J and MGIT, were used to increase the diagnostic rate of TB[38]. We also applied a strict diagnostic definition in calculating specificity by considering only patients with smear-negative and culture-negative results[39].

Conclusions

Formatted: Font: (Default) Times New Roman, 12 pt
Field Code Changed
Formatted: Font: 11 pt
Field Code Changed
Field Code Changed
Formatted: Font: (Default) Times New Roman, 12 pt
Formatted: Font: 11 pt
Formatted: Font: (Default) Times New Roman
Field Code Changed
Formatted: Font: (Default) Times New Roman, 12 pt
Field Code Changed
Formatted: Font: (Default) Times New Roman, 12 pt
Field Code Changed
Formatted: Font: (Default) Times New Roman, 12 pt

In conclusion, the LAMP test is a practical and affordable nucleic amplification technique for the diagnosis of pulmonary tuberculosis, which should be implemented in resource-limiting settings where Xpert MTB/RIF is unavailable. The diagnostic accuracy of the in-hose LAMP was similar to previous studies for specificity. Better sputum processing and DNA extraction method should be identified to improve the test sensitivity. -The ~~pragmatic diagnostic~~overall accuracy of the in-house LAMP test was comparable to that of conventional microscopy and fluorescence microscopy with minimal inferiority in terms of sensitivity. ~~To rule in TB diagnosis~~Therefore, a ~~serial~~ parallel examination of both smear microscopy and the in-house LAMP test is suggested to minimize the risk of false-~~positive~~ negative results, especially in an endemic area.

Acknowledgements

The authors wish to acknowledge the contributions of all the medical and nursing staff of the tuberculosis clinic at Maesot hospital for their help in data collection, and all relevant personnel of The National Institute of Health, Department of Medical Science, The Ministry of Public Health for their technical advice and support.

References

1. World Health Organization. Global Tuberculosis Report 2018. World Health Organization; 2018.

2. Uplekar M, Weil D, Lonnroth K, Jaramillo E, Lienhardt C, Dias HM, et al. WHO's new end TB strategy. Lancet Lond Engl. 2015;385: 1799–1801. doi:10.1016/S0140-6736(15)60570-0

3. Parsons LM, Somoskövi Á, Gutierrez C, Lee E, Paramasivan CN, Abimiku A, et al. Laboratory Diagnosis of Tuberculosis in Resource-Poor Countries: Challenges and Opportunities. Clin Microbiol Rev. 2011;24: 314. doi:10.1128/CMR.00059-10

4. Kim C-K, Cho EA, Shin DM, Choi SW, Shin SY. Comparative Evaluation of the Loop-Mediated Isothermal Amplification Assay for Detecting Pulmonary Tuberculosis. Ann Lab Med. 2018;38: 119–124. doi:10.3343/alm.2018.38.2.119

5. Eddabra R, Ait Benhassou H. Rapid molecular assays for detection of tuberculosis. Pneumonia. 2018;10. doi:10.1186/s41479-018-0049-2

6. Shete PB, Farr K, Strnad L, Gray CM, Cattamanchi A. Diagnostic accuracy of TB-LAMP for pulmonary tuberculosis: a systematic review and meta-analysis. BMC Infect Dis. 2019;19: 268. doi:10.1186/s12879-019-3881-y

7. Notomi T, Okayama H, Masubuchi H, Yonekawa T, Watanabe K, Amino N, et al. Loop-mediated isothermal amplification of DNA. Nucleic Acids Res. 2000;28: e63.

8. Yan L, Xiao H, Zhang Q. Systematic review: Comparison of Xpert MTB/RIF, LAMP and SAT methods for the diagnosis of pulmonary tuberculosis. Tuberc Edinb Scotl. 2016;96: 75–86. doi:10.1016/j.tube.2015.11.005

Formatted: Font: Times New Roman

Formatted: Bibliography, Line spacing: Double, Widow/Orphan control, Adjust space between Latin and Asian text, Adjust space between Asian text and numbers

9. The Use of Loop-Mediated Isothermal Amplification (TB-LAMP) for the Diagnosis of Pulmonary Tuberculosis: Policy Guidance. Geneva: World Health Organization; 2016. Available: http://www.ncbi.nlm.nih.gov/books/NBK384520/

10. Sohn H, Puri L, Nguyen NAT, Van't Hoog AH, Nguyen VAT, Nliwasa M, et al. Cost and affordability analysis of TB-LAMP and Xpert MTB/RIF assays as routine diagnostic tests in peripheral laboratories in Malawi and Vietnam. J Glob Health Sci. 2019;1: e22. doi:10.35500/jghs.2019.1.e22

11. Pandey BD, Poudel A, Yoda T, Tamaru A, Oda N, Fukushima Y, et al. Development of an in-house loop-mediated isothermal amplification (LAMP) assay for detection of Mycobacterium tuberculosis and evaluation in sputum samples of Nepalese patients. J Med Microbiol. 2008;57: 439–443. doi:10.1099/jmm.0.47499-0

12. George G, Mony P, Kenneth J. Comparison of the Efficacies of Loop-Mediated Isothermal Amplification, Fluorescence Smear Microscopy and Culture for the Diagnosis of Tuberculosis. PLoS ONE. 2011;6. doi:10.1371/journal.pone.0021007

13. Rudeeaneksin J, Bunchoo S, Srisungngam S, Sawanpanyalert P, Chamnangrom S, Kamolwat A, et al. Rapid identification of Mycobacterium tuberculosis in BACTEC MGIT960 cultures by in-house loop-medicated isothermal amplification. Jpn J Infect Dis. 2012;65: 306–311. doi:10.7883/yoken.65.306

14. Nagai K, Horita N, Yamamoto M, Tsukahara T, Nagakura H, Tashiro K, et al. Diagnostic test accuracy of loop-mediated isothermal amplification assay for Mycobacterium tuberculosis: systematic review and meta-analysis. Sci Rep. 2016;6: 39090. doi:10.1038/srep39090

15. Phetsuksiri B, Rudeeaneksin J, Srisungngam S, Bunchoo S, Klayut W, Nakajima C, et al. Comparison of Loop-Mediated Isothermal Amplification, Microscopy, Culture, and PCR for Diagnosis of Pulmonary Tuberculosis. Jpn J Infect Dis. 2020;advpub. doi:10.7883/yoken.JJID.2019.335

16. Kent PT. Public Health Mycobacteriology: A Guide for the Level III Laboratory. U.S. Department of Health and Human Services, Public Health Service, Centers for Disease Control; 1985.

17. Phetsuksiri B, Rudeeaneksin J, Srisungngam S, Bunchoo S, Roienthong D, Mukai T, et al. Applicability of in-house loop-mediated isothermal amplification for rapid identification of Mycobacterium tuberculosis complex grown on solid media. Jpn J Infect Dis. 2013;66: 249–251. doi:10.7883/yoken.66.249

18. Rudeeaneksin J. Rapid Detection of Mycobacterium Tuberculosis Complex in Sputum and in Culture Growth by LAMP Test. J Health Sci - วารสารวิชาการสาธารณสุข. 2017;19: 300–310.

19. Rafati A, Gill P. Microfluidic method for rapid turbidimetric detection of the DNA of Mycobacterium tuberculosis using loop-mediated isothermal amplification in capillary tubes. Microchim Acta. 2015;182: 523–530. doi:10.1007/s00604-014-1354-y

20. Poudel A, Pandey BD, Lekhak B, Rijal B, Sapkota BR, Suzuki Y. Clinical profiling and use of loop-mediated isothermal amplification assay for rapid detection of Mycobacterium tuberculosis from sputum. Kathmandu Univ Med J KUMJ. 2009;7: 109–114. doi:10.3126/kumj.v7i2.2701

21. Sethi SK, Singh S, Dhatwalia SK, Yadav R, Mewara A, Singh M, et al. Evaluation of In- House Loop- Mediated Isothermal Amplification (LAMP) Assay for Rapid Diagnosis of M. tuberculosis in Pulmonary Specimens. J Clin Lab Anal. 2013;27: 272–276. doi:10.1002/jcla.21596

22. Li Y, Shi L, Pan A, Cao W, Chen X, Meng H, et al. Evaluation of real-time loop-mediated isothermal amplification (RealAmp) for rapid detection of Mycobacterium tuberculosis from sputum samples. J Microbiol Methods. 2014;104: 55–58. doi:10.1016/j.mimet.2014.06.011

23. Lee M-F, Chen Y-H, Peng C-F. Evaluation of reverse transcription loop-mediated isothermal amplification in conjunction with ELISA-hybridization assay for molecular detection of

Mycobacterium tuberculosis. J Microbiol Methods. 2009;76: 174–180. doi:10.1016/j.mimet.2008.10.005

24. Kohan L, Shahhosseiny MH, Razavi MR, Parivar K, Moslemi E, Werngren J. Evaluation of loop mediated isothermal amplification for diagnosis of *Mycobacterium tuberculosis* complex in clinical samples. Afr J Biotechnol. 2011;10: 5096–5101. doi:10.4314/ajb.v10i26.

25. Habeenzu C, Nakajima C, Solo E, Bwalya P, Kajino K, Miller M, et al. Evaluation of in-house loop-mediated isothermal amplification for tuberculosis diagnosis compared with Xpert MTB/RIF. J Infect Dev Ctries. 2017;11: 440–444. doi:10.3855/jidc.7730

26. Pandey BD, Poudel A, Yoda T, Tamaru A, Oda N, Fukushima Y, et al. Development of an in-house loop-mediated isothermal amplification (LAMP) assay for detection of Mycobacterium tuberculosis and evaluation in sputum samples of Nepalese patients. J Med Microbiol. 2008;57: 439–443. doi:10.1099/jmm.0.47499-0

27. Pathak D, Chakravorty S, Hanif M, Tyagi JS. Lysis of tubercle bacilli in fresh and stored sputum specimens: implications for diagnosing tuberculosis in stored and paucibacillary specimens by PCR. BMC Microbiol. 2007;7: 83. doi:10.1186/1471-2180-7-83

28. Shea YR, Davis JL, Huang L, Kovacs JA, Masur H, Mulindwa F, et al. High Sensitivity and Specificity of Acid-Fast Microscopy for Diagnosis of Pulmonary Tuberculosis in an African Population with a High Prevalence of Human Immunodeficiency Virus. J Clin Microbiol. 2009;47: 1553–1555. doi:10.1128/JCM.00348-09

29. Pham TH, Peter J, Mello FCQ, Parraga T, Lan NTN, Nabeta P, et al. Performance of the TB-LAMP diagnostic assay in reference laboratories: Results from a multicentre study. Int J Infect Dis IJID Off Publ Int Soc Infect Dis. 2018;68: 44–49. doi:10.1016/j.ijid.2018.01.005

30. Gray CM, Katamba A, Narang P, Giraldo J, Zamudio C, Joloba M, et al. Feasibility and Operational Performance of Tuberculosis Detection by Loop-Mediated Isothermal

Amplification Platform in Decentralized Settings: Results from a Multicenter Study. J Clin Microbiol. 2016;54: 1984–1991. doi:10.1128/JCM.03036-15

31. Pai M, Flores LL, Pai N, Hubbard A, Riley LW, Colford JM. Diagnostic accuracy of nucleic acid amplification tests for tuberculous meningitis: a systematic review and meta-analysis. Lancet Infect Dis. 2003;3: 633–643. doi:10.1016/s1473-3099(03)00772-2

32. Shi J, Dong W, Ma Y, Liang Q, Shang Y, Wang F, et al. GeneXpert MTB/RIF Outperforms Mycobacterial Culture in Detecting Mycobacterium tuberculosis from Salivary Sputum. In: BioMed Research International [Internet]. 2018 [cited 12 Dec 2019]. Available: https://www.hindawi.com/journals/bmri/2018/1514381/

33. Ho J, Marks GB, Fox GJ. The impact of sputum quality on tuberculosis diagnosis: a systematic review. Int J Tuberc Lung Dis Off J Int Union Tuberc Lung Dis. 2015;19: 537–544. doi:10.5588/ijtld.14.0798

34. Yoon SH, Lee NK, Yim JJ. Impact of sputum gross appearance and volume on smear positivity of pulmonary tuberculosis: a prospective cohort study. BMC Infect Dis. 2012;12: 172. doi:10.1186/1471-2334-12-172

35. Hirooka T, Higuchi T, Tanaka N, Ogura T. [The value of proper sputum collection instruction in detection of acid-fast bacillus]. Kekkaku. 2004;79: 33–37.

36. Sicsú AN, Salem JI, Fujimoto LBM, Gonzales RIC, Cardoso M do S de L, Palha PF. Educational intervention for collecting sputum for tuberculosis: a quasi-experimental study 1. Rev Lat Am Enfermagem. 2016;24. doi:10.1590/1518-8345.0363.2703

37. Cudahy P, Shenoi S. Diagnostics for pulmonary tuberculosis. Postgrad Med J. 2016;92: 187–193. doi:10.1136/postgradmedj-2015-133278

38. Kadioglu EE, Ucar EY, Araz O, Aktas E, Saglam L. A Comparison of Two Different Culture Methods for Use in the Diagnosis of Pulmonary Tuberculosis. Eurasian J Med. 2014;46: 74–77. doi:10.5152/eajm.2014.19

39. Kaku T, Minamoto F, D'Meza R, Morose W, Boncy J, Bijou J, et al. Accuracy of LAMP-TB Method for Diagnosing Tuberculosis in Haiti. Jpn J Infect Dis. 2016;69: 488–492. doi:10.7883/yoken.JJID.2015.519

1. World Health Organization. Global Tuberculosis Report 2018. World Health Organization; 2018.

2. Uplekar M, Weil D, Lonnroth K, Jaramillo E, Lienhardt C, Dias HM, et al. WHO's new end TB strategy. Lancet Lond Engl. 2015;385: 1799–1801. doi:10.1016/S0140-6736(15)60570-0

3. Parsons LM, Somoskövi Á, Gutierrez C, Lee E, Paramasivan CN, Abimiku A, et al. Laboratory Diagnosis of Tuberculosis in Resource-Poor Countries: Challenges and Opportunities. Clin Microbiol Rev. 2011;24: 314. doi:10.1128/CMR.00059-10

4. Kim C-K, Cho EA, Shin DM, Choi SW, Shin SY. Comparative Evaluation of the Loop-Mediated Isothermal Amplification Assay for Detecting Pulmonary Tuberculosis. Ann Lab Med. 2018;38: 119–124. doi:10.3343/alm.2018.38.2.119

5. Pai M, Flores LL, Pai N, Hubbard A, Riley LW, Colford JM. Diagnostic accuracy of nucleic acid amplification tests for tuberculous meningitis: a systematic review and meta-analysis. Lancet Infect Dis. 2003;3: 633–643. doi:10.1016/s1473-3099(03)00772-2

6. Eddabra R, Ait Benhassou H. Rapid molecular assays for detection of tuberculosis. Pneumonia. 2018;10. doi:10.1186/s41479-018-0049-2

Formatted: Line spacing: Double

7. Shete PB, Farr K, Strnad L, Gray CM, Cattamanchi A. Diagnostic accuracy of TB-LAMP for pulmonary tuberculosis: a systematic review and meta-analysis. BMC Infect Dis. 2019;19: 268. doi:10.1186/s12879-019-3881-y

8. Notomi T, Okayama H, Masubuchi H, Yonekawa T, Watanabe K, Amino N, et al. Loop-mediated isothermal amplification of DNA. Nucleic Acids Res. 2000;28: e63.

9. Yan L, Xiao H, Zhang Q. Systematic review: Comparison of Xpert MTB/RIF, LAMP and SAT methods for the diagnosis of pulmonary tuberculosis. Tuberc Edinb Scotl. 2016;96: 75–86. doi:10.1016/j.tube.2015.11.005

10. The Use of Loop-Mediated Isothermal Amplification (TB-LAMP) for the Diagnosis of Pulmonary Tuberculosis: Policy Guidance. Geneva: World Health Organization; 2016. Available: http://www.ncbi.nlm.nih.gov/books/NBK384520/

11. George G, Mony P, Kenneth J. Comparison of the Efficacies of Loop-Mediated Isothermal Amplification, Fluorescence Smear Microscopy and Culture for the Diagnosis of Tuberculosis. PLoS ONE. 2011;6. doi:10.1371/journal.pone.0021007

12. Ou X, Li Q, Xia H, Pang Y, Wang S, Zhao B, et al. Diagnostic accuracy of the PURE-LAMP test for pulmonary tuberculosis at the county-level laboratory in China. PloS One. 2014;9: e94544. doi:10.1371/journal.pone.0094544

13. Rudeeaneksin J, Bunchoo S, Srisungngam S, Sawanpanyalert P, Chamnangrom S, Kamolwat A, et al. Rapid identification of Mycobacterium tuberculosis in BACTEC MGIT960 cultures by in-house loop-medicated isothermal amplification. Jpn J Infect Dis. 2012;65: 306–311. doi:10.7883/yoken.65.306

14. Nagai K, Horita N, Yamamoto M, Tsukahara T, Nagakura H, Tashiro K, et al. Diagnostic test accuracy of loop-mediated isothermal amplification assay for Mycobacterium tuberculosis: systematic review and meta-analysis. Sci Rep. 2016;6: 39090. doi:10.1038/srep39090

15. Kent PT. Public Health Mycobacteriology: A Guide for the Level III Laboratory. U.S. Department of Health and Human Services, Public Health Service, Centers for Disease Control; 1985.

16. Phetsuksiri B, Rudeeaneksin J, Srisungngam S, Bunchoo S, Roienthong D, Mukai T, et al. Applicability of in-house loop-mediated isothermal amplification for rapid identification of Mycobacterium tuberculosis complex grown on solid media. Jpn J Infect Dis. 2013;66: 249–251. doi:10.7883/yoken.66.249

17. Rudeeaneksin J. Rapid Detection of Mycobacterium Tuberculosis Complex in Sputum and in Culture Growth by LAMP Test. J Health Sci. 2017;19: 300–310.

18. Pandey BD, Poudel A, Yoda T, Tamaru A, Oda N, Fukushima Y, et al. Development of an in-house loop-mediated isothermal amplification (LAMP) assay for detection of Mycobacterium tuberculosis and evaluation in sputum samples of Nepalese patients. J Med Microbiol. 2008;57: 439–443. doi:10.1099/jmm.0.47499-0

19. Pathak D, Chakravorty S, Hanif M, Tyagi JS. Lysis of tubercle bacilli in fresh and stored sputum specimens: implications for diagnosing tuberculosis in stored and paucibacillary specimens by PCR. BMC Microbiol. 2007;7: 83. doi:10.1186/1471-2180-7-83

20. Shea YR, Davis JL, Huang L, Kovacs JA, Masur H, Mulindwa F, et al. High Sensitivity and Specificity of Acid-Fast Microscopy for Diagnosis of Pulmonary Tuberculosis in an

African Population with a High Prevalence of Human Immunodeficiency Virus. J Clin Microbiol. 2009;47: 1553–1555. doi:10.1128/JCM.00348-09

21. Pham TH, Peter J, Mello FCQ, Parraga T, Lan NTN, Nabeta P, et al. Performance of the TB-LAMP diagnostic assay in reference laboratories: Results from a multicentre study. Int J Infect Dis IJID Off Publ Int Soc Infect Dis. 2018;68: 44–49. doi:10.1016/j.ijid.2018.01.005

22. Gray CM, Katamba A, Narang P, Giraldo J, Zamudio C, Joloba M, et al. Feasibility and Operational Performance of Tuberculosis Detection by Loop-Mediated Isothermal Amplification Platform in Decentralized Settings: Results from a Multicenter Study. J Clin Microbiol. 2016;54: 1984–1991. doi:10.1128/JCM.03036-15

23. Horita N, Yamamoto M, Sato T, Tsukahara T, Nagakura H, Tashiro K, et al. Sensitivity and specificity of Cobas TaqMan MTB real-time polymerase chain reaction for culture-proven Mycobacterium tuberculosis: meta-analysis of 26999 specimens from 17 Studies. Sci Rep. 2015;5. doi:10.1038/srep18113

24. Habeenzu C, Nakajima C, Solo E, Bwalya P, Kajino K, Miller M, et al. Evaluation of in-house loop-mediated isothermal amplification for tuberculosis diagnosis compared with Xpert MTB/RIF. J Infect Dev Ctries. 2017;11: 440–444. doi:10.3855/jidc.7730

25. Shi J, Dong W, Ma Y, Liang Q, Shang Y, Wang F, et al. GeneXpert MTB/RIF Outperforms Mycobacterial Culture in Detecting Mycobacterium tuberculosis from Salivary Sputum. BioMed Res Int. 2018;2018. doi:10.1155/2018/1514381

Supporting information

S1 Table. Review on diagnostic accuracy of in-house LAMP assays for diagnosis of pulmonary tuberculosis (DOCX)

S2 Table. LAMP minimal dataset (CSV)

Formatted: Font: 18 pt, Bold

Formatted: Font: Bold

Formatted: Font: Bold

Formatted: Line spacing: Double

Formatted: Normal, Space After: 8 pt, Line spacing: Double

Responses to Reviewers' comments

Pragmatic accuracy of loop-mediated isothermal amplification (LAMP) for diagnosis of pulmonary tuberculosis in a Thai community hospital

Reviewer #1:

1. The study aims to evaluate usefulness of a LAMP method in a practical setting in Thailand. The LAMP method is now available as an only commercial kit TB-LAMP assay (Loopamp™MTBC Detection Kit, Eiken Chemical Company Ltd., Japan) as endorsed by WHO in 2016. It seems that the method used in this study is a unique system at least partially. So, it is important to state explicitly that the target to be evaluated was an in-house LAMP and not one commercially available LAMP recommended by WHO.
    - The LAMP test in our study was a non-commercial, in-house LAMP.
    - We re-wrote the manuscript and emphasized that the test used was in-house LAMP.
2. In evaluating the sensitivity of the method, the authors used culture negative (clinically defined) cases, as well as bacteriologically confirmed cases, as a gold standard of the cases of TB. It may be difficult to admit the clinical diagnosis as a diagnostic basis for such a study as this, apart from clinical practice. Vice versa, the definition of the gold (conventional) standard for specificity (non-cases) should be reconsidered. The following paper may be of use in revising the paper; Kaku et al: Accuracy of LAMP-TB Method for Diagnosing Tuberculosis in Haiti. Jpn. J. Infect. Dis., 69, 488–492, 2016.
    - We modified the inclusion criteria for analysis as suggested by both reviewers.
    - As the analysis was done in a per-patient fashion, patients with smear-positive and culture-negative results would be excluded, as these patients were considered as probable TB cases. Therefore, the evaluation of sensitivity would include patients with both smear positive and smear negative with positive culture results. In contrast, the evaluation of specificity would include only patients with smear-negative and culture-negative results.

Reviewer #2:

1. Abstract/Background: “proven diagnostic performance” – this is both vague and too specific at the same time, “most of the results were validated” – the results aren’t validated, the assay is validated
    - We rewrote the abstract and introduction part as suggested.
2. The language surrounding people with possible TB needs to be updated throughout the paper - avoid the use of terms like "TB suspects" that increase the stigma surrounding this disease. http://www.stoptb.org/assets/documents/resources/publications/acsm/LanguageGuide_ForWeb20131110.pdf
    - We rewrote the abstract and introduction part as suggested.
3. The paper states repeatedly that there is little work published from resource-challenged settings, but this claim is not supported. Even the references given cite studies in such decentralized settings. Maybe it just hasn’t been done in Thailand? A better summary of the literature needs to be included. How does this compare to other studies? How is the TB LAMP test performed in this study compare to the TB LAMP tests in other published literature? A better focus on properly relating the current study to the body of work in the literature rather than trying to claim it is quite novel would actually strengthen the paper. There is merit in replication or demonstrating an important diagnostic in a new geographical area.
    - We rewrote the abstract and introduction part as suggested.
4. In-house vs commercialized kit is mentioned but not explained. And the position of this paper (what LAMP testing approach is used) is not properly placed in the context of what other papers are using and the potential impact on sensitivity/specificity.
    - We rewrote the abstract and introduction part as suggested.
5. The sensitivity/specificity of LAMP in other papers, settings, etc needs to be stated with numbers and not just alluded to. A proper, specific summary of the literature is lacking.
    - We rewrote the abstract and introduction part as suggested.
6. “In 2016, WHO suggested the use of LAMP assay for the diagnosis of pulmonary tuberculosis” – this is not quite right, WHO recommendations are very specific and it is important to get that right. From the abstract of the citation provided: “WHO recommends that TB-LAMP can be used as a replacement for microscopy for the diagnosis of pulmonary TB in adults with signs and symptoms of TB”. This needs to be stated correctly. Also, given the paper has mentioned in-house vs commercialized kits, it needs to be clarified that the WHO guidance refers only to the Eiken LAMP kit.
    - We rewrote the abstract and introduction part as suggested.
7. “LAMP assay has a low cost per test, does not required advanced technological facilities, and can be routinely practiced in general hospital laboratories [3].” Reference 3 doesn’t support this statement – it doesn’t say anywhere that the LAMP assay has a low cost per test. It says “Costs can be kept to a minimum if testing is limited to specimens from the most high-risk patients based on proper clinical assessments and national testing algorithms based on public health policies.” There are other publications on the cost of the LAMP assay for TB diagnosis. The authors might explain better the infrastructure/training needed for LAMP based on this reference and others.
    - We rewrote the abstract and introduction part as suggested.
    - We changed the references to the statement as follow: Sohn H. Cost, affordability, and cost-effectiveness of TB-LAMP assay. In: Report to WHO Guideline Development Group Meeting on TB-LAMP Assay. Edn. Geneva: World Health Organization; 2016 and Shete PB, Farr K, Strnad L, Gray CM, Cattamanchi A. Diagnostic accuracy of TB-LAMP for pulmonary tuberculosis: a systematic review

and meta-analysis. BMC Infect Dis. 2019;19(1):268. Published 2019 Mar 19. doi:10.1186/s12879-019-3881-y

8. Reference 5 doesn't appear to really relate to the sentences it comes after. Reference 3 would make a lot more sense as it is a detailed overview of TB diagnostics including many molecular diagnostics.
    - We rewrote the abstract and introduction part as suggested.

Setting

1. The paper needs to do more to state what sets this setting apart from (or ties it to) other studies. See the methods section describing setting in reference 22 for how attributes of the specific site can be expressed in the context of the needs of LAMP.
    - We elaborated the character of our setting as suggested:
    - Level of health system: rural
    - Distance to reference laboratory: 0 km
    - Median LAMP test workload: 6 (4-10)
    - Electricity and backup power: infrequent power outages, power generator (350 Kw) and UPS (2.7 Kw)
    - Biosafety cabinet infrastructure: BSC class II
    - Laboratory staff: 4 lab technicians, 1 lab assistant
2. Study Design: This is not a cross-sectional design; it is a prospective design. The plan was to prospectively enroll 120 patients.
    - We changed the type of design to prospective diagnostic accuracy study as suggested.
    - We would like to make a constructive argument on this point, as the diagnostic accuracy research is actually cross-sectional study in design. The cross-sectional design is only the type of membership condition, single component of study base, and cross-sectional design can therefore be collected prospectively or retrospectively. We would like to ask you to kindly refer to this reference: Assessment of the accuracy of diagnostic tests: the cross-sectional study by Knottnerus JA, 2003.
      Link: https://www.ncbi.nlm.nih.gov/pubmed/14615003
3. "New patients who were clinically suspected of 109 pulmonary TB (coughing for more than two weeks with or without hemoptysis), aged more than 18 years old were consecutively invited into the study regardless of nation status." Suggest re-writing to something more like: 'Adults more than 18yrs of age with symptoms indicative of pulmonary TB (coughing…) and no history of TB were consecutively enrolled regardless of national status.' If patients were 'invited' but not enrolled, we need numbers on how many declined.
    - We re-wrote the sentence as suggested: Adult patients aged more than 15 years old with symptoms indicative of pulmonary TB (coughing for more than two weeks with or without hemoptysis) and no history of TB were consecutively enrolled regardless of national status.
4. "Samples with contaminated culture results or samples from patients who were previously documented as TB cases were excluded." Were the patients excluded or the samples?
    - Patients with previously documented TB cases were excluded.
    - Patients with two contaminated or missing culture results were excluded.

Methods

1. A map of which samples were used for what tests would be quite helpful. Highlight if any of the reference tests (smear, LJ culture, MGIT culture) were performed on the same sputum as LAMP.
    - Conventional macroscopy, LAMP test, and culture were conducted as routinely done.

- All patients were given three sealed containers for the collection of morning sputum specimens. Of all containers sent to the laboratory, only the one with seemingly adequate sputum, containing both mucoid or mucopurulent characters with a sample volume more than 3 ml, was used for the whole investigation procedures as routinely done. Specimens were sent for smear microscopy with conventional acid-fast bacilli (AFB) staining with Ziehl-Neelsen technique and fluorescence acid-fast staining with Auramine O solution.

2. Make it clear somewhere that smear-negative refers to AFB smear-negative.
    - We added detail on the smear-negative status as suggested.
    - According to WHO definitions, any patient with at least two AFB smears of scanty grade or one or more smears of 1+ or more was defined as smear-positive case. Smear-negative case was conversely defined.
3. Study size estimation
   This has no purpose here – the study is done. Sample size estimation is for study planning purposes, for securing funding and making sure the plan has statistical validity.
    - The study size estimation part was removed as suggested.
4. Statistical analysis. The first four sentences are unnecessary.
    - The first four sentences were removed as suggested.
5. The authors need to state what method was used to obtain the 95% CI for the sens/spec/PPV/NPV/LR+. It is clear from my testing that the Clopper Pearson binomial exact test was used, the authors should include the reference (usually found in the software documentation).
    - The 95% confidence intervals were calculated using the Clopper Pearson binomial exact method.
    - We added this statement in the statistical section and added the citation as suggested.
6. Kappa statistics are for inter-reader reliability, not for comparison of correlations between tests. It includes the concept that agreement may happen by chance when two people are guessing. However, it is not appropriate for comparison of diagnostic results because there isn't guessing – the samples should not agree by chance but because they are or are not TB and the sensitivities of tests objectively vary. Spearman's correlation can be used, but I think what you actually want is McNemar's test. The desire is to compare the diagnostic performance (i.e. accuracy) between tests – McNemar's test will do that. Alternatively, Spearman's correlation can look at the [objective] agreement between tests.
    - Spearman's rank correlation was inserted into the manuscript to represent the objective agreement between tests as suggested.
    - The agreement of LAMP test with smear microscopy methods was analyzed with Kappa's statistics and Spearman's rank correlation.
    - We still presented the value of Kappa's statistics as many of the previous studies on LAMP assay and other diagnostic tests had done [1–3].

Results

1. Table 1 is dedicated to showing the patient clinical characteristics by culture status. The p-values shown test whether these characteristics differ significantly dependent on culture status. It is expected that gender, nationality, and age should not differ. Whereas it is also expected that chest x-rays and sputum quality would differ. The baseline demographic data between culture188 positive and negative patients were comparable except for the presence of cavitary lesions on 189 chest radiographs and the character of collected sputum (Table 1). Age, nationality, and gender are demographic data. Chest x-ray and sputum quality are clinical characteristics.
    - We reanalyzed all the data after exclusion of patients with probable TB (LAMP test positive and AFB smear positive patients with negative culture).
    - All the baseline demographic and clinical characteristics data were reanalyzed and presented in Table 1.
    - The statements in the results section were re-written as suggested.
2. Table 2 – re-check the NPV for parallel testing
    - We reanalyzed all the data after exclusion of patients with probable TB (LAMP test positive and AFB smear positive patients with negative culture).
    - All the data on Table 2 were checked for any error as suggested.
3. There are a lot of LAMP-positive and AFB smear-positive patients with negative culture. Especially given that the tests are done on different sputum samples, these should be considered patients with probable TB and not used in assessing sensitivity and specificity.
    - We reanalyzed all the data after exclusion of patients with probable TB (LAMP test positive and AFB smear positive patients with negative culture).
    - The final study size for analysis of LAMP test diagnostic accuracy was therefore 107 patients. (8 patients were excluded, 6 patients with both LAMP test and AFB smear-positive and culture negative, 1 patient with AFB positive and culture negative, and 1 patient with fluorescence stain positive and culture negative)
4. There are too few smear-negative, culture-positive patients to assess sensitivity. Specificity should not be stratified by smear status, only sensitivity. For the reason above (that smear-positive, culture-negative patients shouldn't be included in estimations of sensitivity/specificity of LAMP), what the paper is calling 'smear-negative specificity' should in fact be reported as the actual specificity of LAMP.
    - We exclude smear-positive, culture negative patients from the analysis as suggested.
    - We reported the actual specificity of LAMP test without stratification.
    - We acknowledged that our there are too few smear negative, culture positive patients to assess sensitivity in the discussion part.
5. Table 2 – the p-values shown have no real meaning! If you want to compare accuracy of tests, you cannot do a p-value over the final accuracy measures among a bunch of tests. You need to compare tests 1 against another by using 2x2 grids and McNemar's test. So, if you want to compare the accuracy of LAMP to the accuracy of AFB stain, you use the grid in Table 3 and McNemar's test:
    - The comparison of diagnostic indices between LAMP test and AFB, fluorescence stain was re-analyzed using McNemar's exact probability test as suggested. We presented the result of the pairwise tests separately and reformatted Table 2.
    - Pairwise testing was not performed to compare the specificity between the LAMP test and the smear microscopy methods as the specificity of the latter was affected by incorporation bias and would not be comparable to the in-house LAMP.
    - Table 3 was also reformatted.
    - Spearman's rank correlation was used as suggested.

Discussion

1. “This study had demonstrated the pragmatic performance of the LAMP test, which was comparable to that of the conventional smear microscopy and the fluorescence microscopy.” Not true, the performance of LAMP as evaluated in this study was below that of smear microscopy.
    - We rewrote the discussion part as suggested.
    - “This study had demonstrated the pragmatic diagnostic performance of the in-house LAMP assay in a remote hospital of a high TB burden country. It was revealed that the overall sensitivity of the in-house LAMP in our study was lower than the numbers reported in the majority of the previous in-house LAMP studies. Nonetheless, the specificity was comparable to other figures reported in literature. In comparison to microscopy methods, the AFB and fluorescence stain, the in-house LAMP was found to be inferior in terms of overall sensitivity (82.0% vs. 88.0%, p=0.375) and accuracy (88.8% vs. 94.4%, p=1.000); however, the comparative statistical test revealed non-significant results. Based on the result of our study, we suggest that the in-house LAMP should not be a substitute to conventional smear methods, but should be done in parallel, which would result in a higher sensitivity with fewer false-negative TB cases.”
2. “Although the sensitivity and specificity of the LAMP test were lower than that of the acid-fast stain and the fluorescence stain, the comparative statistical test revealed non-significant results” This is still true when McNemar’s test is performed, but the right statistical tests need to be used in the paper. Furthermore, a non-significant result doesn't mean no difference, it means the difference is likely smaller than the power of the study to detect.
    - We rewrote the discussion part as suggested.
    - We reanalyzed our data using McNemar’s exact probability test as suggested.
3. Put PPV/NPV in the context of the local prevalence of disease! State from the literature or reliable source what the prevalence of TB is in the hospital’s area of Thailand. I would suggest giving the readers an example: Given that prevalence and a group of 1000 patients, state how many would be true positives, false positive, true negatives, and false negatives. You can therefore assess what burden the different accuracies will place on the hospital. I.e. if the specificity is quite low and the sensitivity is higher, is that better? If the sensitivity is high and the specificity is lower, is that better? Relate this to the LR+.
    - We would like to make a constructive argument to this question as follow: The prevalence of culture-positive TB in this study was 46.7%. As this was a “consecutive recruitment of patients with sign and symptoms suggestive of pulmonary TB” or “patients with higher pre-test probability that the general prevalence” or the “person that the in-house LAMP test was intended to be used”, the calculation of positive predictive values could be directly calculated and reported from the study data as in the other study [1]. Moreover, both the in-house LAMP assay and acid-fast stain were not intended to be used as screening tests in the general population. For this reason, we did not include this part in our manuscript; however, we provide the answer to the question in this response paper.
    - The latest Maesot’s population figures from the Health Data Center (HDC), the ministry of public health, Thailand, was 115,108 in 2019. The prevalence of pulmonary tuberculosis was 351 per 100,000 or 35 per 10,000.

	TB case	Non-TB case	Total	
LAMP positive	29	528	557	PPV 29/557=5.2%
LAMP negative	6	9,437	9,443	NPV 9437/9443=94.9%

Total	35	9,965	10,000	Prevalence=0.0035

4. "In the clinical context of TB diagnosis, both the LAMP test and the smear microscopy are considered as a diagnostic test which would normally be done in TB suspects with high pre-test probability [14]" – this is not what the reference says.
    - The reference states "The TB LAMP assay is usually applied for TB-suspected patients and is rarely used for screening purpose. To rule-in the TB diagnosis, specificity is more important than sensitivity."
    - What we're trying to imply from this statement was that the LAMP test was developed to be applied for patients who were suspicious of having TB with "higher pre-test probability than average person". As the LAMP test was not for screening purpose, specificity is more important and should be more focused than sensitivity.
    - After we re-analyzed the data with the exclusion of probable TB cases, our specificity increased to comparable level with previous studies. The parallel and serial testing was omitted from our analysis as the test accuracy of combination of the in-house LAMP with other smear microscopy methods would be seriously affected by incorporation bias (smear-positive, culture-negative patients were all excluded.
5. "Therefore, a serial test relying on both the result from the LAMP test and the acid-fast stain would be more appropriate for use as a rule-in test as it carried higher specificity and positive likelihood ratio than other methods." Authors should define 'rule-in' test and what is generally expected of such a test. Should note the increased cost of such an approach.
    - After we re-analyzed the data with the exclusion of probable TB cases, our specificity increased to comparable level with previous studies. The parallel and serial testing was omitted from our analysis as the test accuracy of combination of the in-house LAMP with other smear microscopy methods would be seriously affected by incorporation bias (smear-positive, culture-negative patients were all excluded.
6. The effect of a gold standard which is not itself perfect should be discussed. Also the variability between sputum samples should be discussed.
    - The use of routine TB culture as a reference standard might be inadequate, as some TB patients could be classified as not having TB [6]. Different culture media and techniques could be used in composite to achieve different performance characteristics[4]. With a higher quality reference standard, the sensitivity of the in-house LAMP should be increased when a portion of three remaining false-positive cases was re-classified as true-positive cases.
    - This study had a higher proportion of salivary sputum than mucous sputum. This could affect the diagnostic performance of both the index and the reference test[5]. The percentage of culture-positive TB cases was lower in salivary samples than in mucous samples (35.8% vs. 65.0%, p=0.005). Both the quality and quantity of sputum specimens were associated with positivity of smear, molecular testing methods (Xpert MTB/RIF and PCR), and TB culture [6,7]. Thus, it was possible that some patients with pulmonary TB might be classified as smear-negative, LAMP-negative, or even culture-negative cases. Interestingly, it was revealed from our data that the proportion of smear-positive, LAMP-positive results was also significantly lower in salivary sputum than in mucous sputum (31.3% vs 57.5%, p=0.009 and 29.9% vs. 60.0%, p=0.003, respectively). Therefore, the sensitivity and accuracy of all tests, including LAMP, might be underestimated. Previous studies reported that by improving the sputum quality, TB diagnostic yield increased[8,9]. Therefore, high-quality sputum collection must be encouraged both in practice and studies.
7. A better look at the differences between this study and others with better test performance needs to be done.
    - In this study, the sensitivity of the in-house LAMP test was 82.0% (95%CI 68.6-91.4) in culture-positive TB patients, respectively. In the past, several studies had reported

a higher sensitivity of the in-house LAMP test, which ranges from 90.0 to 100.0%. Most of these studies were either University hospital, TB-specialized centers or hospitals, or national TB-specialized laboratory, which were generally equipped with highly-trained personnel and adequate infrastructural supports. The overall sensitivity of our in-house LAMP was consistent with two previous studies from India and Zambia, which was 79.5% (95%CI 64.0-89.0) and 81.4% (95%CI 71.6-89.0), respectively. Although both studies were performed in University hospitals, the LAMP procedures were modified to suit local conditions, and sputum processing and DNA extraction was done with commercial kits. The higher sensitivity of the acid-fast stain and the fluorescence stain in our study could be explained by the high prevalence of TB, the absence of HIV patient or a smaller number of patients with paucibacillary sputum, and the availability of skilled technicians

8. "Currently, the WHO only supported the use of two rapid molecular tests for the diagnosis of 294 pulmonary tuberculosis, which were Xpert MTB/RIF and the LAMP test" – as the concept of LAMP test from a kit and other LAMP tests has been raised, and the variability of accuracy depending, it needs to be clear that the WHO recommendation is only for the Eiken LAMP test kit!
    - We edited the statement as follow: "Currently, the WHO only supported the use of two rapid molecular tests for the diagnosis of pulmonary tuberculosis, which were Xpert MTB/RIF and the commercialized TB-LAMP assay".

References

1. George G, Mony P, Kenneth J. Comparison of the Efficacies of Loop-Mediated Isothermal Amplification, Fluorescence Smear Microscopy and Culture for the Diagnosis of Tuberculosis. PLoS ONE. 2011;6. doi:10.1371/journal.pone.0021007

2. Phetsuksiri B, Rudeeaneksin J, Srisungngam S, Bunchoo S, Klayut W, Nakajima C, et al. Comparison of Loop-Mediated Isothermal Amplification, Microscopy, Culture, and PCR for Diagnosis of Pulmonary Tuberculosis. Jpn J Infect Dis. 2020;advpub. doi:10.7883/yoken.JJID.2019.335

3. Wang Z, Sun H, Ren Z, Xue B, Lu J, Zhang H. Feasibility and Performance of Loop-Mediated Isothermal Amplification Assay in the Diagnosis of Pulmonary Tuberculosis in Decentralized Settings in Eastern China. BioMed Res Int. 2019;2019. doi:10.1155/2019/6845756

4. Cudahy P, Shenoi S. Diagnostics for pulmonary tuberculosis. Postgrad Med J. 2016;92: 187–193. doi:10.1136/postgradmedj-2015-133278

5. Shi J, Dong W, Ma Y, Liang Q, Shang Y, Wang F, et al. GeneXpert MTB/RIF Outperforms Mycobacterial Culture in Detecting Mycobacterium tuberculosis from Salivary Sputum. In: BioMed Research International [Internet]. 2018 [cited 12 Dec 2019]. Available: https://www.hindawi.com/journals/bmri/2018/1514381/

6. Ho J, Marks GB, Fox GJ. The impact of sputum quality on tuberculosis diagnosis: a systematic review. Int J Tuberc Lung Dis Off J Int Union Tuberc Lung Dis. 2015;19: 537–544. doi:10.5588/ijtld.14.0798

7. Yoon SH, Lee NK, Yim JJ. Impact of sputum gross appearance and volume on smear positivity of pulmonary tuberculosis: a prospective cohort study. BMC Infect Dis. 2012;12: 172. doi:10.1186/1471-2334-12-172

8. Hirooka T, Higuchi T, Tanaka N, Ogura T. [The value of proper sputum collection instruction in detection of acid-fast bacillus]. Kekkaku. 2004;79: 33–37.

9. Sicsú AN, Salem JI, Fujimoto LBM, Gonzales RIC, Cardoso M do S de L, Palha PF. Educational intervention for collecting sputum for tuberculosis: a quasi-experimental study 1. Rev Lat Am Enfermagem. 2016;24. doi:10.1590/1518-8345.0363.2703
