# Supplementary material for: Pragmatic accuracy of an in-house loop-mediated isothermal amplification (LAMP) for diagnosis of pulmonary tuberculosis in a Thai community hospital
Source: PLoS One. 2020 Jul 23;15(7):e0236496. doi: 10.1371/journal.pone.0236496 (PMC7377475; doi:10.1371/journal.pone.0236496)
Supplement: Attachment — Submitted filename: PONE-D-20-00432_R2_reviewed.pdf [file pone.0236496.s007.pdf]

PLOS ONE

Pragmatic accuracy of an in-house loop-mediated isothermal amplification (LAMP) for diagnosis of pulmonary tuberculosis in a Thai community hospital

--Manuscript Draft--

**Manuscript Number:**	PONE-D-20-00432R2
**Article Type:**	Research Article
**Full Title:**	Pragmatic accuracy of an in-house loop-mediated isothermal amplification (LAMP) for diagnosis of pulmonary tuberculosis in a Thai community hospital
**Short Title:**	Diagnostic accuracy of an in-house LAMP for pulmonary TB
**Corresponding Author:**	Phichayut Phinyo<br>Chiang Mai University<br>Chiang Mai, Chiang Mai THAILAND
**Keywords:**	Pulmonary Tuberculosis, In-House LAMP, Diagnosis, Sensitivity, Specificity
**Abstract:**	Background: To improve the quality of diagnosing pulmonary tuberculosis (TB), WHO recommends the use of rapid molecular testing as an alternative to conventional microscopic methods. Loop-mediated isothermal amplification assay (LAMP test) is a practical and cost-effective nucleic amplification technique. We evaluated the pragmatic accuracy of an in-house LAMP assay for the diagnosis of TB in a remote health care setting where an advanced rapid molecular test is not available.<br>Methods: A prospective diagnostic accuracy study was conducted. Patients with clinical symptoms suggestive of TB were consecutively enrolled from April to August 2016. Sputum samples were collected from each patient and were sent for microscopic examination (both acid-fast stain and fluorescence stain), in-house LAMP test, and TB culture.<br>Results: One hundred and seven patients with TB symptoms were used in the final analysis. This included 50 (46.7%) culture-positive TB patients and 57 (53.3%) culture-negative patients. The overall sensitivity of the in-house LAMP based on culture positivity was 88.8% (95/107) with a 95%CI of 81.2-94.1. The sensitivity was 90.9% (40/44) with a 95%CI of 78.3-97.5 for smear-positive, culture-positive patients, and was 16.7% (1/6) with a 95%CI of 0.4-64.1 for smear-negative, culture-positive patients. The overall sensitivity of the in-house LAMP test compared to smear microscopy methods were not significantly different (p=0.375). The specificity of the in-house LAMP based on non-TB patients (smear-negative, culture-negative) was 94.7% (54/57) with a 95%CI of 85.4-98.9.<br>Conclusions: The diagnostic accuracy of the in-house LAMP test in a community hospital was comparable to other previous reports in terms of specificity. The sensitivity of the in-house assay could be improved with better sputum processing and DNA extraction method.
**Order of Authors:**	Sarawut Toonkomdang
	Phichayut Phinyo
	Benjawan Phetsuksiri
	Jayanton Patumanond
	Janisara Rudeeaneksin
	Wiphat Klayut
**Response to Reviewers:**	Response to Reviewers<br>We want to thank both the editor and the reviewers for granting the opportunity to revise our manuscript for publication in the PLOS one journal. We hope that our responses and revisions would substantially improve the quality of our manuscript and would be qualified for publication in the journal. If there were any further questions or minor points to be addressed or elaborated, please let us know. We would be more than eager to make any further revision.<br>Editor’s comments<br>Specific comments:

Powered by Editorial Manager® and ProduXion Manager® from Aries Systems Corporation

1.Abstract and Tables: Change the format 95%CI to be consistent with the rest of the manuscript, for example “95%CI 78.3,97.5” should be “95%CI 78.3-97.5”.
•Changed as suggested.

2.Line 80, implicated has negative meaning, suggest changing to implemented.
•We modified the first two sentences as “As financial resources are usually limited in countries with high TB prevalence, a commercial TB-LAMP could still be unattainable. More affordable in-house LAMP assays were later developed and applied in several centers”.

3.Line 162 change M. tuberculosis to Mycobacterium tuberculosis since this is the first time you mentioned the bacteria. Also, all the “M.” need to be italicized in M. tuberculosis throughout the manuscript.
•Corrected as suggested.

4.Line 192 – 193, should the “smear-positive and culture-positive results” be “smear-positive and culture-negative results”?
•Corrected as suggested.

5.Line 243 – 246: These sentences need reference(s)
•We inserted some references to the two sentences as suggested.
Reviewer’s comments
Thank you to the authors for the revisions made. This is a much better paper to present what is important work. However, I still have a few concerns. These focus on clarification of the ‘in-house assay’ and the discussion. Additionally, I think a review of the paper by a medical writer or any strong English editor would boost the communication of the results enormously.
1.The paper needs to be reviewed in detail for grammar and English. Other than general tidiness, in a number of places, the intent of what the authors are saying is lost due to odd grammar choices. For the best readability and better reach for the research contained, a review of the writing is recommended. I have made a few notes and suggestions in specific places.
a.We corrected all of your English suggestions.
b.We also modified and re-written some of the sentences in the manuscript to improve the readability.
2.The difference in assays still needs to be clearer. An ‘in-house assay’ is one that is not performed from a kit. You refer to ‘the in-house’ LAMP assay a lot as if there is only one, which is not the case. There are many papers out there with different ‘in-house’ LAMP assays. From the introduction, it sounds like you are presenting the findings from an in-house assay you developed following the protocol presented in Pandey et al. If so, this needs to be stated very clearly. However, from the methods section, it does not necessarily sound like you are not following that protocol and that this is a unique in-house assay. Please clarify in the paper.
a.We made the modification and improved the clarity of our in-house LAMP method as suggested.
3.When discussing previous results and meta-analyses, it needs to be clear that these refer to ‘in-house LAMP assays’ and not ‘the in-house LAMP assay’ as they are not uniform.
a.Corrected as suggested.
4.Inclusion of ‘Accuracy’ in Table 2 is a bit odd, but it can be kept if it is defined in the statistical methods section.
a.It was pre-specified in the methods section.
5.The discussion has a lengthy discourse on the costs of Xpert vs LAMP. But there is no referencing of the studies that have costed these two in order to make a proper comparison. It feels quite unsupported.
a.We removed unsupported statements from the paragraph and make the paragraph more concise.
6.In the discussion, the authors state ‘No previous study had officially addressed the effect of sputum quality on the LAMP test’. I’m not sure this is true and would caution the authors not to make such a sweeping statement.
a.We removed the sentence out of the discussion section as suggested.
7.In the discussion, “Interestingly, it was revealed from our data that the proportion of smear-positive, LAMP-positive results was also significantly lower in salivary sputum than in mucous sputum (31.3% vs. 57.5%, p=0.009 and 29.9% vs. 60.0%, p=0.003,

Powered by Editorial Manager® and ProduXion Manager® from Aries Systems Corporation

	respectively). Therefore, the sensitivity and accuracy of all tests, including LAMP, might be underestimated." 1) Do not present new results in the discussion – these need to be included in the Results section first. 3) are these sensivity? Specificity? Accuracy? 3) This is not an interpretation that makes sense. The sensitivity/specificity is reported based on the best sputum sample available from the patients – quality samples are difficult to obtain. You can instead interpret it as 'Sensitivity and specificity would be improved if higher quality sputum is obtained'.<br>a.We modified the content as suggested.<br>b.We moved the findings to the results section.<br>8.In general, the discussion needs to be revised to make only statements supported by the literature, the study, or a comparison of the two. Much of the discussion feels like the authors musings.<br>a.We modified the whole discussion sections to be as objective as possible.<br>9.In the discussion, I would suggest focusing on sensitivity and specificity and not accuracy as accuracy is not a common way of discussing or assessing diagnostic tests due to its difficulty of interpretation.<br>a.Corrected as suggested.<br>10.The references as displayed in the reference section aren't quite right. In reference #1, instead of Lancet, the Journal is listed as Lancet Lond Engl which is not correct. This inclusion of a city occurs in reference #7 as well.<br>a.Corrected as suggested.
**Additional Information:**	
**Question**	**Response**
**Financial Disclosure**<br><br>Enter a financial disclosure statement that describes the sources of funding for the work included in this submission. Review the submission guidelines for detailed requirements. View published research articles from *PLOS ONE* for specific examples.<br><br>This statement is required for submission and **will appear in the published article** if the submission is accepted. Please make sure it is accurate.	The author(s) received no specific funding for this work.

**Unfunded studies**<br>Enter: *The author(s) received no specific funding for this work.*<br><br>**Funded studies**<br>Enter a statement with the following details:<br>• Initials of the authors who received each award<br>• Grant numbers awarded to each author<br>• The full name of each funder<br>• URL of each funder website<br>• Did the sponsors or funders play any role in the study design, data collection and analysis, decision to publish, or preparation of the manuscript?<br>• **NO** - Include this sentence at the end of your statement: *The funders had no role in study design, data collection and analysis, decision to publish, or preparation of the manuscript.*<br>• **YES** - Specify the role(s) played.<br><br>* typeset	
**Competing Interests**<br><br>Use the instructions below to enter a competing interest statement for this submission. On behalf of all authors, disclose any competing interests that could be perceived to bias this work—acknowledging all financial support and any other relevant financial or non-financial competing interests.<br><br>This statement **will appear in the published article** if the submission is accepted. Please make sure it is accurate. View published research articles from *PLOS ONE* for specific examples.	The authors have declared that no competing interests exist.

**NO authors have competing interests**<br><br>Enter: *The authors have declared that no competing interests exist.*<br><br>**Authors with competing interests**<br><br>Enter competing interest details beginning with this statement:<br><br>*I have read the journal's policy and the authors of this manuscript have the following competing interests: [insert competing interests here]*<br><br>* typeset	
**Ethics Statement**<br><br>Enter an ethics statement for this submission. This statement is required if the study involved:<br><br>• Human participants<br>• Human specimens or tissue<br>• Vertebrate animals or cephalopods<br>• Vertebrate embryos or tissues<br>• Field research<br><br>Write "N/A" if the submission does not require an ethics statement.<br><br>General guidance is provided below. Consult the submission guidelines for detailed instructions. **Make sure that all information entered here is included in the Methods section of the manuscript.**	This study was approved by the Research Ethics Committee of Maesot General Hospital, The Ministry of Public Health (serial number 37/2015) and The Human Research Ethics Committee of Thammasat University, Faculty of Medicine (COA number 081/2016). The clinical samples used in this study were collected from all patients as routinely done. Informed consent was obtained from all patients prior to inclusion.

Powered by Editorial Manager® and ProduXion Manager® from Aries Systems Corporation

**Format for specific study types**<br><br>**Human Subject Research (involving human participants and/or tissue)**<br>• Give the name of the institutional review board or ethics committee that approved the study<br>• Include the approval number and/or a statement indicating approval of this research<br>• Indicate the form of consent obtained (written/oral) or the reason that consent was not obtained (e.g. the data were analyzed anonymously)<br><br>**Animal Research (involving vertebrate animals, embryos or tissues)**<br>• Provide the name of the Institutional Animal Care and Use Committee (IACUC) or other relevant ethics board that reviewed the study protocol, and indicate whether they approved this research or granted a formal waiver of ethical approval<br>• Include an approval number if one was obtained<br>• If the study involved *non-human primates*, add *additional details* about animal welfare and steps taken to ameliorate suffering<br>• If anesthesia, euthanasia, or any kind of animal sacrifice is part of the study, include briefly which substances and/or methods were applied<br><br>**Field Research**<br><br>Include the following details if this study involves the collection of plant, animal, or other materials from a natural setting:<br>• Field permit number<br>• Name of the institution or relevant body that granted permission	
**Data Availability**<br><br>Authors are required to make all data underlying the findings described fully available, without restriction, and from the time of publication. PLOS allows rare exceptions to address legal and ethical concerns. See the PLOS Data Policy and FAQ for detailed information.	Yes - all data are fully available without restriction

A Data Availability Statement describing where the data can be found is required at submission. Your answers to this question constitute the Data Availability Statement and **will be published in the article**, if accepted.<br><br>**Important:** Stating 'data available on request from the author' is not sufficient. If your data are only available upon request, select 'No' for the first question and explain your exceptional situation in the text box.<br><br>Do the authors confirm that all data underlying the findings described in their manuscript are fully available without restriction?	
**Describe where the data may be found in full sentences. If you are copying our sample text, replace any instances of XXX with the appropriate details.**<br><br>• If the data are **held or will be held in a public repository**, include URLs, accession numbers or DOIs. If this information will only be available after acceptance, indicate this by ticking the box below. For example: *All XXX files are available from the XXX database (accession number(s) XXX, XXX.*).<br>• If the data are all contained **within the manuscript and/or Supporting Information files**, enter the following: *All relevant data are within the manuscript and its Supporting Information files.*<br>• If neither of these applies but you are able to provide **details of access elsewhere**, with or without limitations, please do so. For example:<br><br>*Data cannot be shared publicly because of [XXX]. Data are available from the XXX Institutional Data Access / Ethics Committee (contact via XXX) for researchers who meet the criteria for access to confidential data.*<br><br>*The data underlying the results presented in the study are available from (include the name of the third party*	All relevant data are within the manuscript and its Supporting Information files.

and contact information or URL).<br>• This text is appropriate if the data are owned by a third party and authors do not have permission to share the data.<br><br>* typeset	
Additional data availability information:	

Manuscript Click here to access/download;Manuscript;Manuscript.docx

Pragmatic accuracy of an in-house loop-mediated isothermal amplification (LAMP) for diagnosis of pulmonary tuberculosis in a Thai community hospital

Short title: Diagnostic accuracy of an in-house LAMP for pulmonary TB

Sarawut Toonkomdang[1], Phichayut Phinyo[2,3*], Benjawan Phetsuksiri[4], Jayanton Patumanond[3], Janisara Rudeeaneksin[4¶], Wiphat Klayut[4¶]

[1] Department of Medical Technology, Maesot General Hospital, Tak, Thailand.

[2]Department of Family Medicine, Faculty of Medicine, Chiang Mai University, Chiang Mai, Thailand.

[3] Center for Clinical Epidemiology and Clinical Statistics, Faculty of Medicine, Chiang Mai University, Chiang Mai, Thailand.

[4] National Institute of Health, Department of Medical Sciences, Ministry of Public Health, Nonthaburi, Thailand.

*Corresponding author:

E-mail: phichayutphinyo@gmail.com (PP)

¶These authors contributed equally to this work.

Abstract

Background: To improve the quality of diagnosing pulmonary tuberculosis (TB), WHO recommends the use of rapid molecular testing as an alternative to conventional microscopic methods. Loop-mediated isothermal amplification assay (LAMP test) is a practical and cost-effective nucleic amplification technique. We evaluated the pragmatic accuracy of an in-house LAMP assay for the diagnosis of TB in a remote health care setting where an advanced rapid molecular test is not available.

Methods: A prospective diagnostic accuracy study was conducted. Patients with clinical symptoms suggestive of TB were consecutively enrolled from April to August 2016. Sputum samples were collected from each patient and were sent for microscopic examination (both acid-fast stain and fluorescence stain), in-house LAMP test, and TB culture.

Results: One hundred and seven patients with TB symptoms were used in the final analysis. This included 50 (46.7%) culture-positive TB patients and 57 (53.3%) culture-negative patients. The overall sensitivity of the in-house LAMP based on culture positivity was 88.8% (95/107) with a 95%CI of 81.2-94.1. The sensitivity was 90.9% (40/44) with a 95%CI of 78.3-97.5 for smear-positive, culture-positive patients, and was 16.7% (1/6) with a 95%CI of 0.4-64.1 for smear-negative, culture-positive patients. The overall sensitivity of the in-house LAMP test compared to smear microscopy methods were not significantly different (p=0.375). The specificity of the in-house LAMP based on non-TB patients (smear-negative, culture-negative) was 94.7% (54/57) with a 95%CI of 85.4-98.9.

Conclusions: The diagnostic accuracy of the in-house LAMP test in a community hospital was comparable to other previous reports in terms of specificity. The sensitivity of the in-house assay could be improved with better sputum processing and DNA extraction method.

Keywords: Pulmonary Tuberculosis, In-House LAMP, Diagnosis, Sensitivity, Specificity

Introduction

Tuberculosis (TB), an airborne communicable disease, has long been considered a significant threat to global public health. According to The World Health Organization (WHO), 10 million people were newly infected with TB in 2018 [1]. The incidence and prevalence of TB vary greatly across the globe, 87% of total cases resided within 30 countries with a high TB burden. In Thailand, the incidence rate was 153 cases per 100,000 population in 2018. Early diagnosis and timely treatment is an essential component of The End TB Strategy endorsed by the WHO, aiming to end the global TB epidemic by the year 2035 [2]. However, TB is still underdiagnosed and undertreated, especially in resource-limited countries, due to the lack of highly sensitive and specific diagnostic tools which are usually expensive and require adequate infrastructure [1,3]. Novel diagnostic methods with enough simplicity and cost-effectiveness are therefore necessary to improve the accurate identification of TB patients in those resource-limited settings [3,4].

Molecular testing methods such as polymerase chain reaction (PCR) or Xpert MTB/RIF have been widely acknowledged as alternative tools to TB culture for the diagnosis of TB patients [3,5]. These nucleic amplification techniques were known for yielding rapid and accurate TB diagnosis. This would overcome the limitations of classical methods, insensitivity for smear microscopy, and lengthy incubation period for TB culture. However, several obstacles remain for the application of these molecular tests as point-of-care testing in community settings. This is because of their complexity to execute and substantial requirements for financial and personnel resources [3,6]. Loop-mediated isothermal amplification (LAMP) assay is another recently developed nucleic acid amplification technique. Unlike PCR, where the amplification of DNA fragment occurs in temperature-dependent steps, the reaction of LAMP

assay functions in isothermal or constant temperature conditions [7,8]. In 2016, WHO endorsed the use of commercial TB-LAMP assay (Eiken Chemical Co., Tokyo, Japan) as a replacement for smear microscopy for the diagnosis of TB [9]. TB-LAMP assay has a low cost per test, does not required advanced technological facilities, and can be routinely practiced in general hospital laboratories [6,10].

As financial resources are usually limited in countries with high TB prevalence, a commercial TB-LAMP could still be unattainable. More affordable in-house LAMP assays were later developed and applied in several centers [11–15]. However, it did require extra-training and skill of technicians to process the clinical specimens. In the past decades, several clinical studies and meta-analyses had evaluated the diagnostic accuracy of in-house LAMP tests for the diagnosis of pulmonary TB [14,16,17] (S1 Table). From the latest meta-analysis, the overall sensitivity and specificity of in-house LAMP was 93.0% (95%CI 88.9-95.7) and 91.8% (95%CI 86.4-95.1), respectively [17]. One recent study in Thailand reported the sensitivity and the specificity of the in-house LAMP at 94.4% (95%CI 88.9-97.7) and 94.3% (95%CI 87.2-98.1), respectively [15]. However, the reported accuracy could be overestimated if it is assessed in qualified laboratories with highly skilled technicians and sufficient resources where molecular tests are usually available [17]. Therefore, this study aimed to evaluate the pragmatic accuracy of the in-house LAMP assay for the diagnosis of pulmonary TB in a community hospital of a developing country with a high TB burden.

Materials and Methods

Ethics Statement

This study was approved by the Research Ethics Committee of Maesot General Hospital, The Ministry of Public Health (serial number 37/2015) and The Human Research Ethics Committee of Thammasat University, Faculty of Medicine (COA number 081/2016). The clinical samples used in this study were collected from all patients as routinely done. Informed consent was obtained from all patients prior to inclusion.

Setting

The study was performed in Maesot General Hospital, a large-sized community hospital with 365 in-patient beds. It is located in Maesot District, Tak Province, which shares the border with Myanmar. The hospital provides standard health care to both Thai and non-Thai patients (Burmese immigrants and ethnic minorities). According to the Health Data Center, the Ministry of Public Health, in Thailand, the incidence rate of pulmonary TB in Maesot was 351 per 100,000 in 2019. The health care system of the hospital is considered rural. Maesot hospital has its reference laboratory with biosafety cabinet infrastructure, BSC class II. There are four lab technicians and one lab assistant within each working shift. Power generator (350 kW) and UPS (2.7 kW) were available in case of power outages, which was infrequent. The median LAMP test workload per day was 6 (range 4-10).

Study Design

This prospective diagnostic accuracy research was conducted from April to August 2016. Adult patients aged more than 15 years old with symptoms indicative of pulmonary TB (coughing for more than two weeks with or without hemoptysis) and no history of TB were consecutively enrolled regardless of nationality status. Patients with previously documented

TB history or patients with two contaminated cultures or missing cultures were excluded from the study.

Methods

All patients were given three sealed containers for the collection of morning sputum specimens. Only one sputum specimen with adequate sputum containing both mucoid or mucopurulent characters and a sample volume of more than 3 ml was selected in all investigation procedures. Specimens were sent for smear microscopy with conventional acid-fast bacilli (AFB) staining with Ziehl-Neelsen technique and fluorescence acid-fast staining with Auramine O solution. The smear-positive case was defined according to WHO definitions as the presence of at least two smears of scanty grade, or one or more smears of 1+ or more. A smear negative case was conversely defined.

Sputum decontamination and culture examination

For the sputum decontamination process, the collected samples and 2% N-Acetyl-L-cysteine (NALC) NaOH were poured into a 50 ml sterile centrifuge tube in an equal proportion. The specimens were subsequently mixed by vortexing for 30 seconds and left at room temperature (20-25℃) for 15 minutes. Then, the test tubes were filled with phosphate buffer saline (pH 6.8) until the volume reached the level of 50 ml. The samples were put in a high-speed refrigerated centrifuge at 3,000 g for 20 minutes. Next, the supernatants were poured off, leaving the tube with decontaminated sputum samples. Finally, a drop (1 ml) of phosphate buffer saline (pH 6.8) was used for resuspension of the specimens.

For TB culture, the reference test, we performed both conventional culture method on L-J (Lowenstein-Jensen) medium and BBL MGIT 960 (mycobacterial growth indicator tube) culture method. The culture media were inoculated with processed sputum specimens and incubated at 35 to 37℃ and monitored weekly for growth until 8 weeks. The sputum samples

were considered as "culture-positive" if growth was detected in either of L-J or MGIT culture, regardless of the smear status. If growth was not detected in neither of the culture methods and both microscopy results were negative, the samples were considered as "culture-negative" or "non-TB patients". Patients with smear-positive and culture-negative, which were generally considered as probable TB, were excluded from the analysis. Both smear microscopy and culture methods were performed according to the standard protocols [18].

In-house LAMP test

The LAMP test consists of three steps as follows: DNA extraction, isothermal amplification, and visual interpretation with fluorescence. In this study, we followed the TB Fast AMP technique, which was developed by the National Institute of Health of Thailand and was described in our previous studies [13,15,19]. The procedures were described as follow. Flexi Gene® DNA Kit (Qiagen co., USA) and Protenase K Kit (Qiagen co., USA) were used for DNA extraction. Six primers were used for the recognition of eight distinct regions on the 16S ribosomal RNA gene of M. *tuberculosis*. Each single LAMP reaction includes 12 µl of TB-Fast AMP mixture (FastAMP master mix includes 2 µl 10Xbuffer, 4 µl 2mM dNTPs, 3.2 µl 5M betaine, 1.2 µl 100 mM $MgSO_4$, 1.6 µl primer mixture), 1 µl *Bst* DNA polymerase enzyme (New England Biolabs, Ipswich MA, USA), 1 µl fluorescent detection reagent (FDR; Eiken Chemical Tokyo, Japan) and 6 µl of extracted DNA samples. Amplification of reaction mixture was performed in the heating blocks at 65℃ for 60 minutes, then examined directly by visual observation. The LAMP assay was considered "positive" if the color of the reaction mixture changed from orange to green, or fluorescence was directly observed with the naked eyes. The test was considered "negative" if the color of the mixture remained unchanged. For quality control, positive control (test tube with *Mycobacterium tuberculosis* genetic materials) and negative control (test tube without *M. tuberculosis* genetic materials) were included in all runs.

Statistical Analysis

We used Fisher's exact probability test for comparison of differences in independent proportions and Student's t-test for two independent means. The sensitivity, specificity, positive predictive values (PPV), negative predictive values (NPV), and positive and negative likelihood ratios were calculated and reported with its 95% confidence interval. The 95% confidence interval were estimated using the Clopper Pearson binomial exact method. The comparison of sensitivity, specificity, and overall test accuracy between the LAMP test and smear microscopy methods was performed with McNemar's exact probability test. Pairwise testing to compare the specificity between the LAMP test and the smear microscopy methods was not performed as the specificity of the latter was affected by incorporation bias and would not be comparable to the in-house LAMP. The inter-rater reliability and the correlation of the LAMP test with smear microscopy methods was analyzed with Kappa's statistics and Spearman's rank correlation, respectively. P-values of less than 0.05 were considered statistically significant. All statistical analyses were done using Stata version 16 (StataCorp, Texas).

Results

A total of 120 patients to be evaluated for TB were consecutively included from April to August 2016. Three patients with two contaminated cultures, two patients who subsequently were detected as previously documented TB cases, and eight patients who had smear-positive and culture-negative results were excluded from the analysis; only 107 patients remained in the study (Fig. 1). Most of the included patients were male (60% vs. 40%) with a mean age of 47. Fifty (46.7%) were culture-positive TB patients and 57 (53.3%) were culture-negative patients. The baseline demographic data between culture-positive and culture-negative patients were comparable (Table 1). For clinical characteristics, the presence of cavitary lesions on chest radiographs and the character of collected sputum was statistically different. Culture-positive TB patients had higher proportion of cavitary lesions (14.0% vs. 1.8%, p=0.024) and mucous sputum specimen (52.0% vs 24.6%, p=0.005) than those with negative TB culture. The proportion of patients with salivary sputum was significantly lower than mucous sputum in both smear-positive and LAMP-positive results (31.3% vs. 57.5%, p=0.009 and 29.9% vs. 60.0%, p=0.003, respectively).

Table 1. Demographic and clinical characteristics of the patients by TB culture status

Characteristics	TB Culture Positive (S+ or S-, C+)	TB Culture Negative (S-, C-)	P-Value
	n=50 (46.7%)	n=57 (53.3%)	
Gender			
Male	30 (60.0)	36 (63.2)	0.842
Female	20 (40.0)	21 (36.8)	
Nationality			
Thai	28 (56.0)	21 (36.8)	0.054
Non-Thai	22 (44.0)	36 (63.2)	
Age (year, mean±SD)	48.7±17.4	45.8±18.7	0.408
Chest radiographs			
Without cavitary lesions	43 (86.0)	56 (98.2)	0.024
With cavitary lesions	7 (14.0)	1 (1.8)	
Character of sputum			
Salivary	24(48.0)	43 (75.4)	0.005
Mucous	26 (52.0)	14 (24.6)	

Abbreviations: TB, tuberculosis; C, culture (+ positive or – negative); S, smear microscopy (+ positive or – negative); SD, standard deviation.

Fig. 1. Study flow diagram of patient enrollment and results of index and reference test based on culture result

The overall sensitivity of the LAMP test was 82.0% (95%CI 68.6-91.4), whereas the sensitivity in smear-positive, culture-positive patients and smear-negative, culture-positive was 90.9% (95%CI 78.3-97.5) and 16.7% (95%CI 0.4-64.1), respectively. The overall sensitivity of both the AFB and the fluorescence stain was slightly higher than that of the LAMP test; however, the differences were non-significant (Table 2). The specificity, positive predictive value, and negative predictive value of the LAMP test was 94.7% (95%CI 85.4-98.9), 93.2% (95%CI 81.3-98.6), and 85.7% (95%CI 74.6-93.3), respectively. The positive and negative likelihood ratios of the LAMP test was 15.6 (95%CI 4.47-82.12) and 0.19 (95%CI 0.08-0.44), respectively. The accuracy measures for the diagnosis of TB cases were shown to vary across different test methods (LAMP test, AFB stain, and fluorescence stain), the differences were without statistical significance (Table 2).

LAMP test results were highly correlated with those of AFB and fluorescence stain (Spearman's rho 0.85, 95%CI 0.74-0.95, $p<0.001$) in the diagnosis of culture-positive TB cases (Table 3). The in-house LAMP also showed substantial to an almost perfect agreement with both microscopy methods in the diagnosis of culture-positive cases (Kappa 0.85, 95%CI 0.74-0.95, $p<0.001$) (Table 3).

Table 2. Diagnostic accuracy of the in-house LAMP test, AFB stain, and Fluorescence stain.

Method	Sensitivity% (95% CI), no. corrects			Specificity% (95%CI), no. corrects	Accuracy% (95%CI), no. corrects (n=107)	PPV% (95%CI)	NPV (95%CI)	LR+ (95%CI)	LR- (95%CI)
	S+, C+ (n=44)	S-, C+ (n=6)	Any S, C+ (n=50)	S-, C- (n=57)					
LAMP	90.9 (78.3,97.5), N=40	16.7 (0.4,64.1), n=1	82.0 (68.6,91.4), n=41	94.7 (85.4,98.9), n=54	88.8 (81.2,94.1), n=95	93.2 (81.3,98.6)	85.7 (74.6,93.3)	15.6 (4.5,82.1)	0.2 (0.1,0.4)
AFB stain	-	-	88.0 (75.7,95.5), n=44	100.0 (93.7,100.0), n=57	94.4 (88.2,97.9), n=101	100.0 (93.7,100.0)	90.5 (80.4,96.4)	-	-
Fluorescence stain	-	-	88.0 (75.7,95.5), n=44	100.0 (93.7,100.0), n=57	94.4 (88.2,97.9), n=101	100.0 (93.7,100.0)	90.5 (80.4,96.4)	-	-
LAMP test vs. AFB stain			P=0.375*	P=0.250*	P=1.000*				
LAMP test vs. Fluorescence stain			P=0.375*	P=0.250*	P=1.000*				

*P-values from McNemar’s Exact probability test

Abbreviations: AFB, acid fast bacilli; C, culture (+ positive or – negative); CI, confidence interval; LAMP, loop-mediated isothermal amplification; LR+, positive likelihood ratio; LR-, negative likelihood ratio; no. correct, number correctly identified; NPV, negative predictive value; PPV, positive predictive value; S, smear microscopy (+ positive or – negative).

Table 3. Inter-rater reliability and diagnostic agreement between an in-house LAMP test and AFB stain-fluorescence stain.

LAMP Test	AFB Stain & Fluorescence stain		
	Positive	Negative	Total
Positive	40	4	44
Negative	4	59	63
Total	44	63	107
Agreement (%)	92.5%		
Kappa (95%CI, p-value)	0.85 (0.74-0.95, p<0.001)		
Spearman's rho (p-value)	0.85 (0.74-0.95, p<0.001)		

Abbreviations: LAMP, loop-mediated isothermal amplification; CI, confidence interval.

Discussion

This study has demonstrated the pragmatic diagnostic performance of our in-house LAMP assay in a remote hospital of a high TB burden country. The overall sensitivity was lower than the majority of the previous in-house LAMP studies [11,15,20–23]. Nonetheless, the specificity was comparable to other figures reported in the literature [11,12,15,21,22]. In comparison to microscopy methods (AFB and fluorescence stain), the in-house LAMP was inferior in terms of overall sensitivity. Based on the result of our study, we suggest that the in-house LAMP should not be a substitute to conventional smear methods, but should be done in parallel, which would result in a higher sensitivity with fewer false-negative TB cases.

In the past, several studies reported a higher sensitivity of in-house LAMP tests, ranging from 90.0 to 100.0% [11,15,20–25]. Most of these studies were reported from either university hospitals, TB-specialized centers or hospitals, or national TB-specialized laboratories, which were generally equipped with highly trained personnel and adequate infrastructural supports [17]. The overall sensitivity of our in-house LAMP was consistent with two previous studies from India and Zambia, which was 79.5% (95%CI 64.0-89.0) and 81.4% (95%CI 71.6-89.0), respectively [12,16]. Although both studies were performed in university hospitals, the LAMP procedures were modified to suit local conditions, and sputum processing and DNA extraction were done with commercial kits. The higher sensitivity of the acid-fast stain and the fluorescence stain in our study could be explained by the high prevalence of TB, the absence of HIV patients or fewer patients with paucibacillary sputum, and the availability of skilled technicians [16,26–28]. Besides, specimen decontamination with concentrated NaOH decreases the amount of viable genetic materials for amplification, which could reduce the sensitivity of both the LAMP test and TB cultures. A lower concentration of NaOH (1-1.5%) or NaOH free methods during sample

decontamination may be suggested [16,29]. The sensitivity of the LAMP test in smear-negative specimens could not be accurately estimated in this study as there were too few smear-negative, culture-positive patients.

The overall specificity of the LAMP test was 94.7% (95%CI 85.4-98.9) for non-TB patients. This was in concordance with a recent meta-analysis, which reported pooled specificity of in-house LAMP tests of 91.8% (95%CI 86.4-95.1) [17]. However, the specificity of the in-house assays was lower than that of the Loopamp commercial kit, which was reported at 96.5% (95%CI 94.7-97.7). A false positive LAMP result in smear-positive cases was frequently encountered in routine practice, which could be explained by multiple factors such as higher temperature, higher humidity, suboptimal reagents volume, and crossover contamination [17,30]. For temperature, only available water bath was applied for temperature controls during LAMP procedures instead of a more stable dry heating block. A recent study suggested a high reaction volume of 30-35 µl due to the risk of self-priming in concentrated reagents [30].

Currently, the WHO only endorses the use of two rapid molecular tests for the diagnosis of pulmonary TB, which were Xpert MTB/RIF and the commercialized TB-LAMP assay [9]. According to previous studies, both had shown comparable performance in smear-positive samples, but higher sensitivity was shown in Xpert MTB/RIF than in the LAMP test [6,12]. Xpert MTB/RIF has been endorsed for use in the diagnosis of TB in many countries, including Thailand [4,31]. However, only a portion of patients, excluding foreigners and ethnic minorities, could reimburse the cost for Xpert MTB/RIF due to the regulation stated by The National Health Security Office (NHSO). To better control the spread of TB, access to rapid diagnostic tools should be provided to all patients with symptoms suggestive of TB [3]. Thus, a LAMP assay may be more applicable in terms of accessibility and affordability, especially in the decentralized areas [4,32].

However, there were some limitations to this study. First, the study size may not be substantial enough to provide the power required to detect a statistically significant difference between tests. Second, no patients with HIV infection were included during the study period, as HIV status could be influential to the diagnostic performance of both the smear microscopy and the LAMP test, especially in areas with a high prevalence of TB-HIV coinfection. Third, there was a higher proportion of salivary sputum than mucous sputum in this study. This could affect the diagnostic performance of both the index and the reference test [33]. Both the quality and quantity of sputum specimens were associated with the positivity of smear, molecular testing methods (Xpert MTB/RIF and PCR), and TB culture [34,35]. Thus, some patients with pulmonary TB might be classified as smear-negative, LAMP-negative, or even culture-negative cases. Sensitivity and specificity would be improved if higher quality sputum is obtained [36,37].

Finally, the use of routine TB culture as a reference standard might be inadequate, as some TB patients could be classified as not having TB [6]. With a higher quality reference standard, the sensitivity of the in-house LAMP should be increased when a portion of three remaining false-positive cases was re-classified as true-positive cases. Different culture media and techniques could be used in composite to achieve different performance characteristics [38]. In our study, two different culture techniques, L-J and MGIT, were used to increase the diagnostic rate of TB [39]. We also applied a strict diagnostic definition in calculating specificity by considering only patients with smear-negative and culture-negative results [40].

Conclusions

In conclusion, a LAMP test is a practical and affordable nucleic amplification technique for the diagnosis of pulmonary TB, which should be implemented in resource-limited settings where Xpert MTB/RIF is unavailable. The diagnostic accuracy of the in-hose LAMP was

similar to previous studies for specificity. To improve the test sensitivity, a better sputum processing and DNA extraction method is essential. The in-house LAMP test had lower sensitivity than smear microscopy. Therefore, a parallel examination of both smear microscopy and the in-house LAMP test is suggested to minimize the risk of false-negative results, especially in an endemic area.

Acknowledgements

The authors wish to acknowledge the contributions of all the medical and nursing staff of the TB clinic at Maesot hospital for their help in data collection, and all relevant personnel of The National Institute of Health, Department of Medical Science, The Ministry of Public Health for their technical advice and support.

References

1. World Health Organization. Global Tuberculosis Report 2018. World Health Organization; 2018.

2. Uplekar M, Weil D, Lonnroth K, Jaramillo E, Lienhardt C, Dias HM, et al. WHO's new end TB strategy. Lancet. 2015;385: 1799–1801. doi:10.1016/S0140-6736(15)60570-0

3. Parsons LM, Somoskövi Á, Gutierrez C, Lee E, Paramasivan CN, Abimiku A, et al. Laboratory Diagnosis of Tuberculosis in Resource-Poor Countries: Challenges and Opportunities. Clin Microbiol Rev. 2011;24: 314. doi:10.1128/CMR.00059-10

4. Kim C-K, Cho EA, Shin DM, Choi SW, Shin SY. Comparative Evaluation of the Loop-Mediated Isothermal Amplification Assay for Detecting Pulmonary Tuberculosis. Ann Lab Med. 2018;38: 119–124. doi:10.3343/alm.2018.38.2.119

5. Eddabra R, Ait Benhassou H. Rapid molecular assays for detection of tuberculosis. Pneumonia. 2018;10. doi:10.1186/s41479-018-0049-2

6. Shete PB, Farr K, Strnad L, Gray CM, Cattamanchi A. Diagnostic accuracy of TB-LAMP for pulmonary tuberculosis: a systematic review and meta-analysis. BMC Infect Dis. 2019;19: 268. doi:10.1186/s12879-019-3881-y

7. Notomi T, Okayama H, Masubuchi H, Yonekawa T, Watanabe K, Amino N, et al. Loop-mediated isothermal amplification of DNA. Nucleic Acids Res. 2000;28: e63.

8. Yan L, Xiao H, Zhang Q. Systematic review: Comparison of Xpert MTB/RIF, LAMP and SAT methods for the diagnosis of pulmonary tuberculosis. Tuberculosis. 2016;96: 75–86. doi:10.1016/j.tube.2015.11.005

9. The Use of Loop-Mediated Isothermal Amplification (TB-LAMP) for the Diagnosis of Pulmonary Tuberculosis: Policy Guidance. Geneva: World Health Organization; 2016. Available: http://www.ncbi.nlm.nih.gov/books/NBK384520/

10. Sohn H, Puri L, Nguyen NAT, Van't Hoog AH, Nguyen VAT, Nliwasa M, et al. Cost and affordability analysis of TB-LAMP and Xpert MTB/RIF assays as routine diagnostic tests in peripheral laboratories in Malawi and Vietnam. J Glob Health Sci. 2019;1: e22. doi:10.35500/jghs.2019.1.e22

11. Pandey BD, Poudel A, Yoda T, Tamaru A, Oda N, Fukushima Y, et al. Development of an in-house loop-mediated isothermal amplification (LAMP) assay for detection of Mycobacterium tuberculosis and evaluation in sputum samples of Nepalese patients. J Med Microbiol. 2008;57: 439–443. doi:10.1099/jmm.0.47499-0

12. Habeenzu C, Nakajima C, Solo E, Bwalya P, Kajino K, Miller M, et al. Evaluation of in-house loop-mediated isothermal amplification for tuberculosis diagnosis compared with Xpert MTB/RIF. J Infect Dev Ctries. 2017;11: 440–444. doi:10.3855/jidc.7730

13. Rudeeaneksin J. Rapid Detection of Mycobacterium Tuberculosis Complex in Sputum and in Culture Growth by LAMP Test. J Health Sci. 2017;19: 300–310.

14. Rudeeaneksin J, Bunchoo S, Srisungngam S, Sawanpanyalert P, Chamnangrom S, Kamolwat A, et al. Rapid identification of Mycobacterium tuberculosis in BACTEC MGIT960 cultures by in-house loop-medicated isothermal amplification. Jpn J Infect Dis. 2012;65: 306–311. doi:10.7883/yoken.65.306

15. Phetsuksiri B, Rudeeaneksin J, Srisungngam S, Bunchoo S, Klayut W, Nakajima C, et al. Comparison of Loop-Mediated Isothermal Amplification, Microscopy, Culture, and PCR for Diagnosis of Pulmonary Tuberculosis. Jpn J Infect Dis. 2020. doi:10.7883/yoken.jjid.2019.335

16. George G, Mony P, Kenneth J. Comparison of the Efficacies of Loop-Mediated Isothermal Amplification, Fluorescence Smear Microscopy and Culture for the Diagnosis of Tuberculosis. PLoS ONE. 2011;6. doi:10.1371/journal.pone.0021007

17. Nagai K, Horita N, Yamamoto M, Tsukahara T, Nagakura H, Tashiro K, et al. Diagnostic test accuracy of loop-mediated isothermal amplification assay for Mycobacterium tuberculosis: systematic review and meta-analysis. Sci Rep. 2016;6: 39090. doi:10.1038/srep39090

18. Kent PT. Public Health Mycobacteriology: A Guide for the Level III Laboratory. U.S. Department of Health and Human Services, Public Health Service, Centers for Disease Control; 1985.

19. Phetsuksiri B, Rudeeaneksin J, Srisungngam S, Bunchoo S, Roienthong D, Mukai T, et al. Applicability of in-house loop-mediated isothermal amplification for rapid identification of Mycobacterium tuberculosis complex grown on solid media. Jpn J Infect Dis. 2013;66: 249–251. doi:10.7883/yoken.66.249

20. Sethi SK, Singh S, Dhatwalia SK, Yadav R, Mewara A, Singh M, et al. Evaluation of In- House Loop- Mediated Isothermal Amplification (LAMP) Assay for Rapid Diagnosis of M. tuberculosis in Pulmonary Specimens. J Clin Lab Anal. 2013;27: 272–276. doi:10.1002/jcla.21596

21. Rafati A, Gill P. Microfluidic method for rapid turbidimetric detection of the DNA of Mycobacterium tuberculosis using loop-mediated isothermal amplification in capillary tubes. Microchim Acta. 2015;182: 523–530. doi:10.1007/s00604-014-1354-y

22. Poudel A, Pandey BD, Lekhak B, Rijal B, Sapkota BR, Suzuki Y. Clinical profiling and use of loop-mediated isothermal amplification assay for rapid detection of Mycobacterium tuberculosis from sputum. Kathmandu Univ Med J KUMJ. 2009;7: 109–114. doi:10.3126/kumj.v7i2.2701

23. Lee M-F, Chen Y-H, Peng C-F. Evaluation of reverse transcription loop-mediated isothermal amplification in conjunction with ELISA-hybridization assay for molecular detection of Mycobacterium tuberculosis. J Microbiol Methods. 2009;76: 174–180. doi:10.1016/j.mimet.2008.10.005

24. Li Y, Shi L, Pan A, Cao W, Chen X, Meng H, et al. Evaluation of real-time loop-mediated isothermal amplification (RealAmp) for rapid detection of Mycobacterium tuberculosis from sputum samples. J Microbiol Methods. 2014;104: 55–58. doi:10.1016/j.mimet.2014.06.011

25. Kohan L, Shahhosseiny MH, Razavi MR, Parivar K, Moslemi E, Werngren J. Evaluation of loop mediated isothermal amplification for diagnosis of *Mycobacterium tuberculosis* complex in clinical samples. Afr J Biotechnol. 2011;10: 5096–5101. doi:10.4314/ajb.v10i26.

26. Pandey BD, Poudel A, Yoda T, Tamaru A, Oda N, Fukushima Y, et al. Development of an in-house loop-mediated isothermal amplification (LAMP) assay for detection of Mycobacterium tuberculosis and evaluation in sputum samples of Nepalese patients. J Med Microbiol. 2008;57: 439–443. doi:10.1099/jmm.0.47499-0

27. Pathak D, Chakravorty S, Hanif M, Tyagi JS. Lysis of tubercle bacilli in fresh and stored sputum specimens: implications for diagnosing tuberculosis in stored and paucibacillary specimens by PCR. BMC Microbiol. 2007;7: 83. doi:10.1186/1471-2180-7-83

28. Shea YR, Davis JL, Huang L, Kovacs JA, Masur H, Mulindwa F, et al. High Sensitivity and Specificity of Acid-Fast Microscopy for Diagnosis of Pulmonary Tuberculosis in an African Population with a High Prevalence of Human Immunodeficiency Virus. J Clin Microbiol. 2009;47: 1553–1555. doi:10.1128/JCM.00348-09

29. Pham TH, Peter J, Mello FCQ, Parraga T, Lan NTN, Nabeta P, et al. Performance of the TB-LAMP diagnostic assay in reference laboratories: Results from a multicentre study. Int J Infect Dis IJID Off Publ Int Soc Infect Dis. 2018;68: 44–49. doi:10.1016/j.ijid.2018.01.005

30. Gray CM, Katamba A, Narang P, Giraldo J, Zamudio C, Joloba M, et al. Feasibility and Operational Performance of Tuberculosis Detection by Loop-Mediated Isothermal Amplification Platform in Decentralized Settings: Results from a Multicenter Study. J Clin Microbiol. 2016;54: 1984–1991. doi:10.1128/JCM.03036-15

31. Pai M, Flores LL, Pai N, Hubbard A, Riley LW, Colford JM. Diagnostic accuracy of nucleic acid amplification tests for tuberculous meningitis: a systematic review and meta-analysis. Lancet Infect Dis. 2003;3: 633–643. doi:10.1016/s1473-3099(03)00772-2

32. Geojith G, Dhanasekaran S, Chandran SP, Kenneth J. Efficacy of loop mediated isothermal amplification (LAMP) assay for the laboratory identification of Mycobacterium tuberculosis isolates in a resource limited setting. J Microbiol Methods. 2011;84: 71–73. doi:10.1016/j.mimet.2010.10.015

33. Shi J, Dong W, Ma Y, Liang Q, Shang Y, Wang F, et al. GeneXpert MTB/RIF Outperforms Mycobacterial Culture in Detecting Mycobacterium tuberculosis from Salivary Sputum. In: BioMed Research International [Internet]. 2018 [cited 12 Dec 2019]. Available: https://www.hindawi.com/journals/bmri/2018/1514381/

34. Ho J, Marks GB, Fox GJ. The impact of sputum quality on tuberculosis diagnosis: a systematic review. Int J Tuberc Lung Dis Off J Int Union Tuberc Lung Dis. 2015;19: 537–544. doi:10.5588/ijtld.14.0798

35. Yoon SH, Lee NK, Yim JJ. Impact of sputum gross appearance and volume on smear positivity of pulmonary tuberculosis: a prospective cohort study. BMC Infect Dis. 2012;12: 172. doi:10.1186/1471-2334-12-172

36. Hirooka T, Higuchi T, Tanaka N, Ogura T. [The value of proper sputum collection instruction in detection of acid-fast bacillus]. Kekkaku. 2004;79: 33–37.

37. Sicsú AN, Salem JI, Fujimoto LBM, Gonzales RIC, Cardoso M do S de L, Palha PF. Educational intervention for collecting sputum for tuberculosis: a quasi-experimental study 1. Rev Lat Am Enfermagem. 2016;24. doi:10.1590/1518-8345.0363.2703

38. Cudahy P, Shenoi S. Diagnostics for pulmonary tuberculosis. Postgrad Med J. 2016;92: 187–193. doi:10.1136/postgradmedj-2015-133278

39. Kadioglu EE, Ucar EY, Araz O, Aktas E, Saglam L. A Comparison of Two Different Culture Methods for Use in the Diagnosis of Pulmonary Tuberculosis. Eurasian J Med. 2014;46: 74–77. doi:10.5152/eajm.2014.19

40. Kaku T, Minamoto F, D’Meza R, Morose W, Boncy J, Bijou J, et al. Accuracy of LAMP-TB Method for Diagnosing Tuberculosis in Haiti. Jpn J Infect Dis. 2016;69: 488–492. doi:10.7883/yoken.JJID.2015.519

Supporting information

S1 Table. Review on diagnostic accuracy of in-house LAMP assays for diagnosis of pulmonary tuberculosis (DOCX)

S2 Table. LAMP minimal dataset (CSV)

Figure 1

Click here to access/download;Figure;Figure 1.png

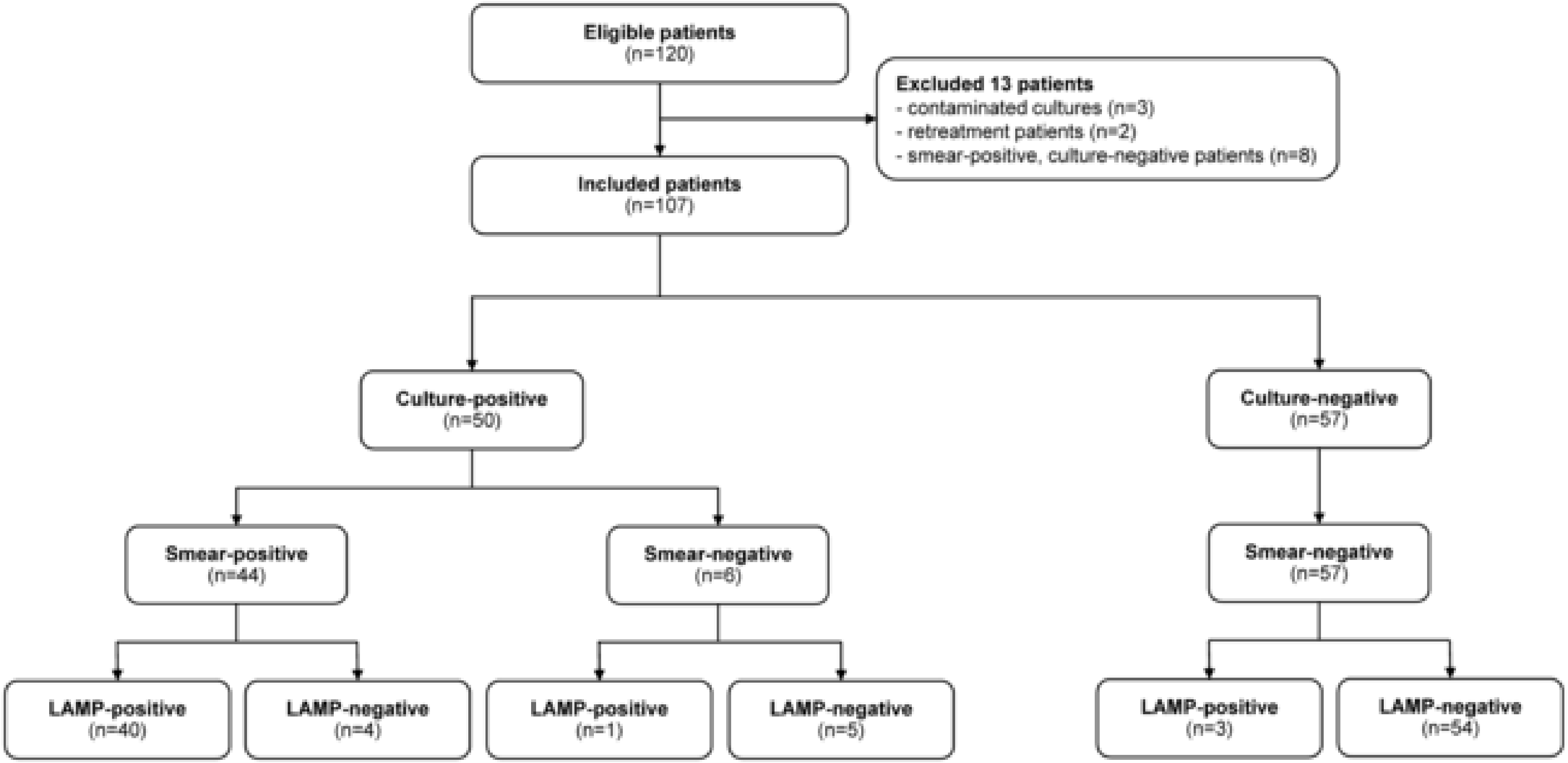

S2 LAMP dataset

Click here to access/download
Supporting Information
S2 LAMP dataset.csv

S1 Review table

Click here to access/download
Supporting Information
Table S1.docx

Pragmatic accuracy of an in-house loop-mediated isothermal amplification (LAMP) for diagnosis of pulmonary tuberculosis in a Thai community hospital

Short title: Diagnostic accuracy of an in-house LAMP for pulmonary TB

Sarawut Toonkomdang[1], Phichayut Phinyo[2,3*], Benjawan Phetsuksiri[4], Jayanton Patumanond[3], Janisara Rudeeaneksin[4¶], Wiphat Klayut[4¶]

[1] Department of Medical Technology, Maesot General Hospital, Tak, Thailand.

[2]Department of Family Medicine, Faculty of Medicine, Chiang Mai University, Chiang Mai, Thailand.

[3] Center for Clinical Epidemiology and Clinical Statistics, Faculty of Medicine, Chiang Mai University, Chiang Mai, Thailand.

[4] National Institute of Health, Department of Medical Sciences, Ministry of Public Health, Nonthaburi, Thailand.

*Corresponding author:

E-mail: phichayutphinyo@gmail.com (PP)

[¶]These authors contributed equally to this work.

Formatted: No underline

Abstract

Background: To improve the quality of diagnosing pulmonary tuberculosis (TB), WHO recommends the use of rapid molecular testing as an alternative to conventional microscopic methods. Loop-mediated isothermal amplification assay (LAMP test) is a practical and cost-effective nucleic amplification technique. We evaluated the pragmatic accuracy of ~~the~~ an in-house LAMP assay for the diagnosis of TB in a remote health care setting where an advanced rapid molecular test is not available.

Methods: A prospective diagnostic accuracy study was conducted. Patients with clinical symptoms suggestive of TB were consecutively enrolled from April to August 2016. Sputum samples were collected from each patient and were sent for microscopic examination (both acid-fast stain and fluorescence stain), in-house LAMP test, and TB culture.

Results: One hundred and seven patients with TB symptoms were used in the final analysis. This included 50 (46.7%) culture-positive TB patients and 57 (53.3%) culture-negative patients. The overall sensitivity of the in-house LAMP based on culture positivity was 88.8% (95/107) ~~(~~with a 95%CI of 81.2~~,~~-94.1~~)~~. The sensitivity was 90.9% (40/44) ~~(~~with a 95%CI of 78.3~~,~~-97.5~~)~~ for smear-positive, culture-positive patients, and was 16.7% (1/6) with a ~~(~~95%CI of 0.4~~,~~-64.1~~)~~ for smear-negative, culture-positive patients. The overall sensitivity ~~and accuracy~~ of the in-house LAMP test compared to smear microscopy methods were not significantly different (p=0.375~~ and p=1.000, respectively~~). The specificity of the in-house LAMP based on non-TB patients (smear-negative, culture-negative) was 94.7% (54/57) with a ~~(~~95%CI of 85.4~~,~~-98.9~~)~~.

Conclusions: The diagnostic accuracy of the in-house LAMP test in a community hospital was comparable to other previous reports in terms of specificity. The sensitivity of the in-house assay could be improved with better sputum processing and DNA extraction method.

Keywords: Pulmonary Tuberculosis, inIn-house House LAMP, Diagnosis, Sensitivity, Specificity

Introduction

Tuberculosis (TB), an airborne communicable disease, has long been considered a significant threat to global public health. According to The World Health Organization (WHO), 10 million people were newly infected with TB in 2018 [1]. ~~Although the~~The incidence and prevalence of TB vary greatly across the globe, 87% of total cases resided within 30 countries with a high TB burden. In Thailand, ~~, including Thailand, where~~ the incidence rate was 153 cases per 100,000 population in 2018~~[1]~~. Early diagnosis and timely treatment is an essential component of The End TB Strategy endorsed by the WHO, aiming to end the global TB epidemic by the year 2035 [2]. However, TB is still underdiagnosed and undertreated, especially in resource-limited~~ing~~ countries, due to the lack of highly sensitive and specific diagnostic tools which are usually expensive and require adequate infrastructure [1,3]. Novel diagnostic methods with enough simplicity and cost-effectiveness are ~~are therefore necessary to improve accurate identification of TB patients in these particular settings~~~~to be~~therefore necessary to improve the accurate identification of TB patients ~~used~~ in those resource-limited settings [3,4].

Molecular testing methods such as polymerase chain reaction (PCR) or Xpert MTB/RIF have been widely acknowledged as alternative tools to TB culture for the diagnosis of TB patients [3,5]. These nucleic amplification techniques were known for yielding rapid and accurate TB diagnosis~~, . which~~ Thi[illegible]uld overcome the limitations of classical methods, insensitivity for smear microscopy, and lengthy incubation period for TB culture. However, several obstacles remain for the application of these molecular tests as point-of-care testing in community settings. Th[illegible] is because of their complexity ~~in executions~~to execute and substantial requirements for financial and personnel resources [3,6]. Loop-mediated isothermal

Formatted: Space After: 8 pt

Field Code Changed

amplification (LAMP) assay is another recently developed nucleic acid amplification technique. Unlike PCR, where the amplification of DNA fragment occurs in temperature-dependent steps, the reaction of LAMP assay functions in isothermal or constant temperature conditions [7,8]. In 2016, WHO suggested endorsed the use of commercial TB-LAMP assay (Eiken Chemical Co., Tokyo, Japan) as a replacement for smear microscopy for the diagnosis of TB in patients with symptoms suggestive of TB [9]. TB-LAMP assay has a low cost per test, does not required advanced technological facilities, and can be routinely practiced in general hospital laboratories [6,10].

As financial resources are usually limited in countries with high TB prevalence, setting up an infrastructure to support the a commercial TB-LAMP could still be unattainable. A moreMore affordable in-house LAMP assays was were later developed and applied in 2008 several centers [11–15][11]. The main advantage of the in-house assay was that it could be implicated on the readily-available infrastructure of any laboratory, even in the decentralized one. However, it did require extra-training and skill of technicians to process the clinical specimens. In the past decades, several clinical studies and meta-analyses had evaluated the diagnostic accuracy of the in-house LAMP tests for the diagnosis of pulmonary TB [14,16,17][12–14] (S1 Table). From the latest meta-analysis, the overall sensitivity and specificity of the in-house LAMP was 93.0% (95%CI 88.9-95.7) and 91.8% (95%CI 86.4-95.1), respectively [17][14]. One recent study in Thailand reported the sensitivity and the specificity of the in-house LAMP at 94.4% (95%CI 88.9-97.7) and 94.3% (95%CI 87.2-98.1), respectively [15]. However, the reported accuracy could be overestimated if being it is assessed in qualified laboratories with highly skilled technicians and sufficient resources where molecular tests are usually are available [17][14]. Therefore, this study aimed to

Formatted: Font: (Default) Times New Roman, 12 pt

Formatted: Font: 11 pt

Formatted: Font: (Default) Times New Roman, 12 pt

Formatted: Font: 11 pt

Formatted: Font: (Default) Times New Roman

evaluate the pragmatic accuracy of the in-house LAMP assay for the diagnosis of pulmonary TB in a ~~peripheral~~ community hospital of a developing country with a high TB burden.

Materials and Methods

Ethics Statement

This study was approved by the Research Ethics Committee of Maesot General Hospital, The Ministry of Public Health (serial number 37/2015) and The Human Research Ethics Committee of Thammasat University, Faculty of Medicine (COA number 081/2016). The clinical samples used in this study were collected from all patients as routinely done. Informed consent was obtained from all patients prior to inclusion.

Setting

The study was settled performed in Maesot General Hospital, a large-sized community hospital with 365 in-patient beds. The hospitalIt [illegible]located in Maesot district District,in Tak Province(province), which shares the border with Myanmar. The hospital and provides standard health care to both Thai and non-Thai patients (Burmese immigrants and ethnic minorities). According to the Health Data Center, the ministry Ministry of public Public healthHealth [illegible]Thailand, the incidence rate of pulmonary TB in Maesot was 351 per 100,000 in 2019. The level o[illegible]lth care system of the hospital is considered rural. Maesot hospital has its [illegible] reference laboratory with biosafety cabinet infrastructure, BSC class II. There are four lab technicians and one lab assistant within each working shift. Power generator (350 kW) and UPS (2.7 kW) were available in case of power outages, which was infrequent. The mMedian LAMP test workload per day was 6 (range 4-10).

Study Design

This prospective diagnostic accuracy research was conducted from April to August 2016. Adult patients aged more than 15 years old with symptoms indicative of pulmonary TB (coughing for more than two weeks with or without hemoptysis) and no history of TB were consecutively enrolled regardless of nationality status. Patients with previously documented TB history or patients with two contaminated cultu[illegible] missing cultures were excluded from the study.

Methods

All patients were given three sealed containers for the collection of morning sputum specimens. Of all containers sent to the laboratory, only the one with seemingly adequate sputum containing both mucoid or mucopurulent characters with a sample volume of more than 3 ml, was used for the whole investigation procedures as routinely done.Only one sputum specimen with adequate sputum containing both mucoid or mucopurulent characters and a sample volume of more than 3 ml was selected to be us[illegible] all investigation procedures. Specimens were sent for smear microscopy with conventional acid-fast bacilli (AFB) staining with Ziehl-Neelsen technique and fluorescence acid-fast staining with Auramine O solution. The sSmear-positive case was defined according to WHO definitions as the presence of at least two smears of scanty grade, or one or more smears of 1+ or more. A smear negative case or AFB smear-negative was conversely defined.

Sputum decontamination and culture examination

For the sputum decontamination process, the collected samples and 2% N-Acetyl-L-cysteine (NALC) NaOH were poured into a 50 ml sterile centrifuge tube in an equal proportion. The and werespecimens were subsequently mixed by vortexing for 30 seconds and left at room

temperature (20-25℃) for 15 minutes. Then, the test tubes were filled with phosphate buffer saline (pH 6.8) until the volume reached the level of 50 ml. The samples were put in a high-speed refrigerated centrifuge at 3,000 g for 20 minutes. Next, the supernatants were poured off, leaving the tube with decontaminated sputum samples. Finally, a drop (1 ml) of phosphate buffer saline (pH 6.8) was used for resuspension of the specimens.

For TB culture, the reference test, we performed both conventional culture method on L-J (Lowenstein-Jensen) medium and BBL MGIT 960 (mycobacterial growth indicator tube) culture method. The culture media were inoculated with processed sputum specimens and incubated at 35 to 37℃ and monitored weekly for growth until 8 weeks. The sputum samples were considered as “culture-positive” if growth was detected in either of L-J or MGIT culture, regardless of the smear status. If growth was not detected in neither of the culture methods and both microscopy results were negative, the samples were considered as “culture-negative” or “non-TB patients”. Patients with smear-positive and culture-negative, which were generally considered as probable TB, were excluded from the analysis. Both smear microscopy and culture methods were performed according to the standard protocols [18][16].

In-house LAMP test

The LAMP test consists of three steps as follows: DNA extraction, isothermal amplification, and visual interpretation with fluorescence. The National Institute of Health of Thailand had developed the TB Fast Amp technique (a modified LAMP procedure) to suite local practice since 2009.In this study, we followed the TB Fast AMP technique, which was developed by the National Institute of Health of Thailand and was described in our previous studies [13,15,19]. -The procedures were described as follow. Flexi Gene® DNA Kit (Qiagen co., USA) and Protenase K Kit (Qiagen co., USA) were used for DNA extraction [17,18]. Four

Formatted: Font: (Default) Times New Roman

Formatted: Font: (Default) Times New Roman

Field Code Changed

Six primers ~~(MTB primers, MAV primers, MIN primers, and Muniv primers)~~ were used for the recognition of ~~six~~ eight distinct regions on the 16S ribosomal RNA gene of M. *tuberculosis*. Each single LAMP reaction includes 12 µl of TB-Fast AMP mixture (FastAMP master mix includes 2 µl 10Xbuffer, 4 µl 2mM dNTPs, 3.2 µl 5M betaine, 1.2 µl 100 mM $MgSO_4$, 1.6 µl primer mixture), 1 µl *Bst* DNA polymerase enzyme (New England Biolabs, Ipswich MA, USA), 1 µl fluorescent detection reagent (FDR; Eiken Chemical Tokyo, Japan) and 6 µl of extracted DNA samples. Amplification of reaction mixture was performed in the heating blocks at 65°C for 60 minutes, then examined directly by visual observation. The LAMP assay was considered "positive" if the color of the reaction mixture changed from orange to green, or fluorescence was directly observed with the naked eyes. The test was considered "negative" if the color of the mixture remained unchanged. For quality control, positive control (test tube with ~~M~~ *Mycobacterium*~~.~~ *tuberculosis* g[illegible]etic materials) and negative control (test tube without *M. tuberculosis* genetic materials) were included in all runs.

Formatted: Font: Italic

Formatted: Font: Italic

Statistical Analysis

We used Fisher's exact probability test for comparison of differences in independent proportions and Student's t-test for two independent means. The sensitivity, specificity, positive predictive values (PPV), negative predictive values (NPV), and positive and negative likelihood ratios were calculated and reported with its 95% confidence interval. The 95% confidence interval were estimated using the Clopper Pearson binomial exact method. The comparison of sensitivity, specificity, and overall test accuracy between the LAMP test and smear microscopy methods was performed with McNemar's exact probability test. Pairwise testing to compare the specificity between the LAMP test and the smear microscopy methods was not performed as the specificity of the latter was affected by incorporation bias and

would not be comparable to the in-house LAMP. The inter-rater reliability and the ~~agreement~~ correlation of the LAMP test with smear microscopy methods was analyzed with Kappa's statistics and Spearman's rank correlation, respectively. P-values of less than 0.05 were considered statistically significant. All statistical analyses were done using Stata version 16 (StataCorp, Texas).

Results

A total of 120 patients to be evaluated for TB were consecutively included from April to August 2016. Three patients with two contaminated cultures, two patients who subsequently were detected as previously documented TB cases, and eight patients who had smear-positive and culture-~~positive~~ negative results were excluded from the analysis; only 107 patients remained in the study (Fig. 1). Most of the included patients were male (60% vs. 40%) with a mean age of 47 ~~year~~. Fifty (46.7%) were culture-positive TB patients and 57 (53.3%) were culture-negative patients. The baseline demographic data between culture-positive and culture-negative patients were comparable (Table 1). For clinical characteristics, the presence of cavitary lesions on chest radiographs and the ~~character of collected sputum~~character of collected sputum was ~~found to be~~statistically ~~significantly~~ differen~~t~~t ~~(Table 1)~~. Culture-positive TB patients had higher proportion of cavitary lesions (14.0% vs. 1.8%, p=0.024) and mucous sputum specimen (52.0% vs 24.6%, p=0.005) than ~~patients~~ those with negative TB culture. The proportion of patients with salivary sputum was significantly lower than mucous sputum in both smear-positive and ~~,~~ LAMP-positive results ~~was significantly lower in salivary sputum than in mucous sputum~~ (31.3% vs. 57.5%, p=0.009 and 29.9% vs. 60.0%, p=0.003, respectively).

Table 1. Demographic and clinical characteristics of the patients by TB culture status

Characteristics	TB Culture Positive (S+ or S-, C+)	TB Culture Negative (S-, C-)	P-Value
	n=50 (46.7%)	n=57 (53.3%)	
Gender			
Male	30 (60.0)	36 (63.2)	0.842
Female	20 (40.0)	21 (36.8)	
Nationality			
Thai	28 (56.0)	21 (36.8)	0.054
Non-Thai	22 (44.0)	36 (63.2)	
Age (year, mean±SD)	48.7±17.4	45.8±18.7	0.408
Chest radiographs			
Without cavitary lesions	43 (86.0)	56 (98.2)	0.024
With cavitary lesions	7 (14.0)	1 (1.8)	
Character of sputum			
Salivary	24(48.0)	43 (75.4)	0.005
Mucous	26 (52.0)	14 (24.6)	

Abbreviations: TB, tuberculosis; C, culture (+ positive or – negative); S, smear microscopy (+ positive or – negative); SD, standard deviation.

Fig. 1. Study flow diagram of patient enrollment and results of index and reference test based on culture result

The overall sensitivity of the LAMP test was 82.0% (95%CI 68.6-91.4), whereas the sensitivity in smear-positive, culture-positive patients and smear-negative, culture-positive was 90.9% (95%CI 78.3-97.5) and 16.7% (95%CI 0.4-64.1), respectively. The overall sensitivity of both the AFB and the fluorescence stain was slightly higher than that of the LAMP test; however, the differences were non-significant (Table 2). The specificity, positive predictive value, and negative predictive value of the LAMP test was 94.7% (95%CI 85.4-98.9), 93.2% (95%CI 81.3-98.6), and 85.7% (95%CI 74.6-93.3), respectively. The positive and negative likelihood ratios of the LAMP test was 15.6 (95%CI 4.47-82.12) and 0.19 (95%CI 0.08-0.44), respectively. ~~Even though the~~The accuracy measures for the diagnosis of TB cases were shown to vary across different test methods (LAMP test, AFB stain, and fluorescence stain), the differences were without statistical significance (Table 2).

LAMP test results were highly correlated with those of AFB and fluorescence stain (Spearman's rho 0.85~~;~~, 95%CI 0.74-0.95, $p<0.001$) in the diagnosis of culture-positive TB cases (Table 3). The in-house LAMP also showed substantial to an almost perfect agreement with both microscopy methods in the diagnosis of culture-positive cases (Kappa 0.85, 95%CI 0.74~~,~~-0.95, $p<0.001$) (Table 3).

Table 2. Diagnostic accuracy of the in-house LAMP test, AFB ~~stain, and~~stain, and Fluorescence stain.

Method	Sensitivity% (95% CI), no. corrects			Specificity% (95%CI), no. corrects	Accuracy% (95%CI), no. corrects (n=107)	PPV% (95%CI)	NPV (95%CI)	LR+ (95%CI)	LR- (95%CI)
	S+, C+ (n=44)	S-, C+ (n=6)	Any S, C+ (n=50)	S-, C- (n=57)					
LAMP	90.9 (78.3,97.5), N=40	16.7 (0.4,64.1), n=1	82.0 (68.6,91.4), n=41	94.7 (85.4,98.9), n=54	88.8 (81.2,94.1), n=95	93.2 (81.3,98.6)	85.7 (74.6,93.3)	15.6 (4.5,82.1)	0.2 (0.1,0.4)
AFB stain	-	-	88.0 (75.7,95.5), n=44	100.0 (93.7,100.0), n=57	94.4 (88.2,97.9), n=101	100.0 (93.7,100.0)	90.5 (80.4,96.4)	-	-
Fluorescence stain	-	-	88.0 (75.7,95.5), n=44	100.0 (93.7,100.0), n=57	94.4 (88.2,97.9), n=101	100.0 (93.7,100.0)	90.5 (80.4,96.4)	-	-
LAMP test vs. AFB stain			P=0.375*	P=0.250*	P=1.000*				
LAMP test vs. Fluorescence stain			P=0.375*	P=0.250*	P=1.000*				

*P-values from McNemar's Exact probability test

Abbreviations: AFB, acid fast bacilli; C, culture (+ positive or – negative); CI, confidence interval; LAMP, loop-mediated isothermal amplification; LR+, positive likelihood ratio; LR-, negative likelihood ratio; no. correct, number correctly identified; NPV, negative predictive value; PPV, positive predictive value; S, smear microscopy (+ positive or – negative).

Table 3. ~~Diagnostic agreement and correlation~~Inter-rater reliability and diagnostic agreement between ~~the~~ an in-house LAMP test and AFB stain-fluorescence stain.

LAMP Test	AFB Stain & Fluorescence stain		
	Positive	Negative	Total
Positive	40	4	44
Negative	4	59	63
Total	44	63	107
Agreement (%)	92.5%		
Kappa (95%CI, p-value)	0.85 (0.74~~,~~0.95, p<0.001)		
Spearman's rho (p-value)	0.85 (0.74-0.95, p<0.001)		

Abbreviations: LAMP, loop-mediated isothermal amplification; CI, confidence interval.

Discussion

This study ~~had~~ has demonstrated the pragmatic diagnostic performance of ~~the~~ our in-house LAMP assay in a remote hospital of a high TB burden country. ~~It was revealed that the~~The overall sensitivity ~~of the in-house LAMP in our study~~ was lower than ~~the numbers reported in~~ the majority of the previous in-house LAMP studies [11,15,20–23]. Nonetheless, the specificity was comparable to other figures reported in the literature [11,12,15,21,22]. ~~-~~In comparison to microscopy methods (~~, the~~ AFB and fluorescence stain), the in-house LAMP was ~~found to be~~ inferior in terms of overall sensitivity~~ (82.0% vs. 88.0%, p=0.375) and accuracy (88.8% vs. 94.4%, p=1.000); however, the comparative statistical test revealed non-significant results~~. Based on the result of our study, we suggest that the in-house LAMP should not be a substitute to conventional smear methods, but should be done in parallel, which would result in a higher sensitivity with fewer false-negative TB cases.

~~In this study, the sensitivity of the in-house LAMP test was 82.0% (95%CI 68.6-91.4) in culture-positive TB patients, respectively.~~ In the past, several studies ~~had~~ reported a higher sensitivity of ~~the~~ in-house LAMP tests, ~~which ranges~~ranging from 90.0 to 100.0% [11,15,20–25]~~[11,15,19–24]~~. Most of these studies were reported from either ~~University~~ university hospitals, TB-specialized centers or hospitals, or national TB-specialized laboratories, which were generally equipped with ~~highly-trained~~highly trained personnel and adequate infrastructural supports [17]. The overall sensitivity of our in-house LAMP was consistent with two previous studies from India and Zambia, which was 79.5% (95%CI 64.0-89.0) and 81.4% (95%CI 71.6-89.0), respectively [12,16]~~[12,25]~~. Although both studies were performed in ~~University~~ university hospitals, the LAMP procedures were modified to suit local conditions, and sputum processing and DNA extraction ~~was~~ were done with commercial kits. The higher sensitivity of the acid-fast stain and the fluorescence stain in our study could be

Formatted: Font: (Default) Times New Roman, 12 pt

Formatted: Font: (Default) Times New Roman, 12 pt

Formatted: Font: (Default) Times New Roman

Formatted: Font: 11 pt

explained by the high prevalence of TB, the absence of HIV patients or ~~less~~ fewer ~~number of~~ patients with paucibacillary sputum, and the availability of skilled technicians [16,26–28]~~[12,26–28]~~. Besides, specimen decontamination with concentrated NaOH decreases the amount of viable genetic materials for amplification, which could reduce the sensitivity of both the LAMP test and TB cultures. A lower concentration of NaOH (1-1.5%) or NaOH free methods during sample decontamination may be suggested [16,29]~~[12,29]~~. The sensitivity of the LAMP test in smear-negative specimens could not be accurately estimated in this study as there were too few smear-negative, culture-positive patients.

The overall specificity of the LAMP test was 94.7% (95%CI 85.4-98.9) for non-TB patients~~, respectively~~. This was in concordance with a recent meta-analysis, which reported pooled specificity of ~~the~~ in-house LAMP tests ~~at~~ of 91.8% (95%CI 86.4-95.1) [17]~~[14]~~. However, ~~it was concluded that~~ the specificity of the in-house assays was lower than that of the Loopamp commercial kit, which was reported at 96.5% (95%CI 94.7-97.7). A false positive LAMP result in smear-positive cases was frequently encountered in routine practice, which could be explained by multiple factors such as higher temperature, higher humidity, suboptimal reagents volume, and crossover contamination [17,30]~~[14,30]~~. ~~For in-house LAMP, an extensive laboratory technician training and continuous quality assessment should be conducted to lessen the risk of false-positive results. However, other potential factors might still account for the low specificity, such as temperature controls and volume of reaction used.~~ For temperature, only available water bath was applied for temperature controls during LAMP procedures instead of a more stable dry heating block. A recent study suggested a high reaction volume of 30-35 µl due to the risk of self-priming in concentrated reagents [30].

Formatted: Font: (Default) Times New Roman

Formatted: Font: (Default) Times New Roman

Formatted: Font: (Default) Times New Roman

Currently, the WHO only ~~supported~~ endorses the use of two rapid molecular tests for the diagnosis of pulmonary TB, which were Xpert MTB/RIF and the commercialized TB-LAMP assay [9]. According to previous studies, both had shown comparable performance in smear-positive samples, but higher sensitivity was shown in Xpert MTB/RIF than in the LAMP test [6,12]~~[6,25]~~. Xpert MTB/RIF has been endorsed for use in the diagnosis of TB in many countries, including Thailand [4,31]. ~~Nonetheless, Xpert MTB/RIF might not be suitable in peripheral regions with poor infrastructure as the instrument requires a stable electricity supply and an appropriate environment. The device also requires high continuous maintenance costs leading to a relatively high cost per test compared to the LAMP test. The LAMP test is readily available and can be done in any resource-poor settings with regular infrastructure and technicians with adequate training. In Thailand,~~However, only a portion of patients, ~~not including~~excluding foreigners and ethnic minorities, could reimburse the cost for Xpert MTB/RIF due to the regulation stated by The National Health Security Office (NHSO). To ~~effectively~~ better ~~prevent~~ control the spread of TB, ~~an~~ access to rapid diagnostic tools should be provided to all patients ~~to be evaluated for TB~~with symptoms suggestive of TB ~~should have equal access to high-quality diagnostic tools~~[3]. ~~Therefore~~~~For this reason~~Thus, a ~~smear microscopy and the~~ LAMP ~~test~~ assay may be more applicable in terms of accessibility and affordability, especially in the ~~distant~~ decentralized areas ~~and the borderlands~~[4,32].

However, there ~~may be~~were some limitations to this study. First, ~~the study size might not be powered enough to confirm the statistical insignificance of the between-test comparison~~the study size may not be substantial enough to provide the power required to detect a statistically significant difference between tests. Second, no patients with HIV infection were included during the study period, as HIV status could be influential to the diagnostic performance of both the smear microscopy and the LAMP test, especially in areas with a high

Formatted: Font: (Default) Times New Roman

Formatted: Font: (Default) Times New Roman, 12 pt

Formatted: Font: (Default) Times New Roman, 12 pt

prevalence of TB-HIV coinfection. Third, ~~this study had~~there was a higher proportion of salivary sputum than mucous sputum in this study. This could affect the diagnostic performance of both the index and the reference test [33]~~[32]~~. ~~The percentage of culture-positive TB cases was lower in salivary samples than in mucous samples (35.8% vs. 65.0%, p=0.005).~~ Both the quality and quantity of sputum specimens were associated with the positivity of smear, molecular testing methods (Xpert MTB/RIF and PCR), and TB culture [34,35]~~[33,34]~~. Thus, ~~it was possible that~~ some patients with pulmonary TB might be classified as smear-negative, LAMP-negative, or even culture-negative cases. Sensitivity and specificity would be improved if higher quality sputum is obtained ~~No previous study had officially addressed the effect of sputum quality on the LAMP test. Moreover, the character of sputum specimens was rarely reported. Interestingly, it was revealed from our data that the proportion of smear-positive, LAMP-positive results was also significantly lower in salivary sputum than in mucous sputum (31.3% vs. 57.5%, p=0.009 and 29.9% vs. 60.0%, p=0.003, respectively). Therefore, the sensitivity and accuracy of all tests, including LAMP, might be underestimated. Previous studies reported that by improving the sputum quality, TB diagnostic yield increased~~ [36,37]~~[35,36]~~. ~~Thus, high-quality sputum collection must be encouraged both in practice and studies.~~

Finally, the use of routine TB culture as a reference standard might be inadequate, as some TB patients could be classified as not having TB [6]. With a higher quality reference standard, the sensitivity of the in-house LAMP should be increased when a portion of three remaining false-positive cases was re-classified as true-positive cases. Different culture media and techniques could be used in composite to achieve different performance characteristics [38]~~[37]~~. In our study, two different culture techniques, L-J and MGIT, were used to increase the diagnostic rate of TB [39]~~[38]~~. We also applied a strict diagnostic definition in calculating

Formatted: Font: (Default) Times New Roman, 12 pt

Formatted: Font: 11 pt

Formatted: Font: (Default) Times New Roman, 12 pt

Formatted: Font: 11 pt

Formatted: Font: (Default) Times New Roman, 12 pt

Formatted: Font: 11 pt

Formatted: Font: (Default) Times New Roman, 12 pt

Formatted: Font: 11 pt

Formatted: Font: (Default) Times New Roman, 12 pt

Formatted: Font: 11 pt

specificity by considering only patients with smear-negative and culture-negative results [40]~~[39]~~.

Formatted: Font: (Default) Times New Roman, 12 pt

Formatted: Font: 11 pt

Conclusions

In conclusion, ~~the~~ a LAMP test is a practical and affordable nucleic amplification technique for the diagnosis of pulmonary TB, which should be implemented in resource-limited~~ing~~ settings where Xpert MTB/RIF is unavailable. The diagnostic accuracy of the in-ho[illegible]AMP was similar to previous studies for specificity. ~~Better sputum processing and DNA extraction method should be identified to improve the test sensitivity.~~To improve the test sensitivity, a better sputum processing and DNA extraction method is essential. -T~~The overall accuracy of t~~he in-house LAMP test ~~showed~~had lower~~minimal inferiority in terms of~~ sensitivity ~~to~~than ~~was comparable to that of conventional microscopy and fluorescence microscopy~~smear microscopy~~ with minimal inferiority in terms of sensitivity~~. Therefore, a parallel examination of both smear microscopy and the in-house LAMP test is suggested to minimize the risk of false-negative results, especially in an endemic area.

Formatted: English (United States)

Acknowledgements

The authors wish to acknowledge the contributions of all the medical and nursing staff of the TB clinic at Maesot hospital for their help in data collection, and all relevant personnel of The National Institute of Health, Department of Medical Science, The Ministry of Public Health for their technical advice and support.

References

1. World Health Organization. Global Tuberculosis Report 2018. World Health Organization; 2018.

2. Uplekar M, Weil D, Lonnroth K, Jaramillo E, Lienhardt C, Dias HM, et al. WHO's new end TB strategy. Lancet Lond Engl. 2015;385: 1799–1801. doi:10.1016/S0140-6736(15)60570-0

3. Parsons LM, Somoskövi Á, Gutierrez C, Lee E, Paramasivan CN, Abimiku A, et al. Laboratory Diagnosis of Tuberculosis in Resource-Poor Countries: Challenges and Opportunities. Clin Microbiol Rev. 2011;24: 314. doi:10.1128/CMR.00059-10

4. Kim C-K, Cho EA, Shin DM, Choi SW, Shin SY. Comparative Evaluation of the Loop-Mediated Isothermal Amplification Assay for Detecting Pulmonary Tuberculosis. Ann Lab Med. 2018;38: 119–124. doi:10.3343/alm.2018.38.2.119

5. Eddabra R, Ait Benhassou H. Rapid molecular assays for detection of tuberculosis. Pneumonia. 2018;10. doi:10.1186/s41479-018-0049-2

6. Shete PB, Farr K, Strnad L, Gray CM, Cattamanchi A. Diagnostic accuracy of TB-LAMP for pulmonary tuberculosis: a systematic review and meta-analysis. BMC Infect Dis. 2019;19: 268. doi:10.1186/s12879-019-3881-y

7. Notomi T, Okayama H, Masubuchi H, Yonekawa T, Watanabe K, Amino N, et al. Loop-mediated isothermal amplification of DNA. Nucleic Acids Res. 2000;28: e63.

8. Yan L, Xiao H, Zhang Q. Systematic review: Comparison of Xpert MTB/RIF, LAMP and SAT methods for the diagnosis of pulmonary tuberculosis. Tuberculosis Edinb Scotl. 2016;96: 75–86. doi:10.1016/j.tube.2015.11.005

9. The Use of Loop-Mediated Isothermal Amplification (TB-LAMP) for the Diagnosis of Pulmonary Tuberculosis: Policy Guidance. Geneva: World Health Organization; 2016. Available: http://www.ncbi.nlm.nih.gov/books/NBK384520/

10. Sohn H, Puri L, Nguyen NAT, Van't Hoog AH, Nguyen VAT, Nliwasa M, et al. Cost and affordability analysis of TB-LAMP and Xpert MTB/RIF assays as routine diagnostic tests in peripheral laboratories in Malawi and Vietnam. J Glob Health Sci. 2019;1: e22. doi:10.35500/jghs.2019.1.e22

11. Pandey BD, Poudel A, Yoda T, Tamaru A, Oda N, Fukushima Y, et al. Development of an in-house loop-mediated isothermal amplification (LAMP) assay for detection of Mycobacterium tuberculosis and evaluation in sputum samples of Nepalese patients. J Med Microbiol. 2008;57: 439–443. doi:10.1099/jmm.0.47499-0

12. Habeenzu C, Nakajima C, Solo E, Bwalya P, Kajino K, Miller M, et al. Evaluation of in-house loop-mediated isothermal amplification for tuberculosis diagnosis compared with Xpert MTB/RIF. J Infect Dev Ctries. 2017;11: 440–444. doi:10.3855/jidc.7730

Formatted: Font: (Default) Times New Roman, 12 pt

Formatted: Bibliography, Widow/Orphan control, Adjust space between Latin and Asian text, Adjust space between Asian text and numbers

Formatted: Font: (Default) Times New Roman, 12 pt

13. Rudeeaneksin J. Rapid Detection of Mycobacterium Tuberculosis Complex in Sputum and in Culture Growth by LAMP Test. J Health Sci. 2017;19: 300–310.

14. Rudeeaneksin J, Bunchoo S, Srisungngam S, Sawanpanyalert P, Chamnangrom S, Kamolwat A, et al. Rapid identification of Mycobacterium tuberculosis in BACTEC MGIT960 cultures by in-house loop-medicated isothermal amplification. Jpn J Infect Dis. 2012;65: 306–311. doi:10.7883/yoken.65.306

15. Phetsuksiri B, Rudeeaneksin J, Srisungngam S, Bunchoo S, Klayut W, Nakajima C, et al. Comparison of Loop-Mediated Isothermal Amplification, Microscopy, Culture, and PCR for Diagnosis of Pulmonary Tuberculosis. Jpn J Infect Dis. 2020. doi:10.7883/yoken.jjid.2019.335~~Phetsuksiri B, Rudeeaneksin J, Srisungngam S, Bunchoo S, Klayut W, Nakajima C, et al. Comparison of Loop-Mediated Isothermal Amplification, Microscopy, Culture, and PCR for Diagnosis of Pulmonary Tuberculosis. Jpn J Infect Dis. 2020;advpub. doi:10.7883/yoken.JJID.2019.335~~

16. George G, Mony P, Kenneth J. Comparison of the Efficacies of Loop-Mediated Isothermal Amplification, Fluorescence Smear Microscopy and Culture for the Diagnosis of Tuberculosis. PLoS ONE. 2011;6. doi:10.1371/journal.pone.0021007

17. Nagai K, Horita N, Yamamoto M, Tsukahara T, Nagakura H, Tashiro K, et al. Diagnostic test accuracy of loop-mediated isothermal amplification assay for Mycobacterium tuberculosis: systematic review and meta-analysis. Sci Rep. 2016;6: 39090. doi:10.1038/srep39090

18. Kent PT. Public Health Mycobacteriology: A Guide for the Level III Laboratory. U.S. Department of Health and Human Services, Public Health Service, Centers for Disease Control; 1985.

19. Phetsuksiri B, Rudeeaneksin J, Srisungngam S, Bunchoo S, Roienthong D, Mukai T, et al. Applicability of in-house loop-mediated isothermal amplification for rapid identification of Mycobacterium tuberculosis complex grown on solid media. Jpn J Infect Dis. 2013;66: 249–251. doi:10.7883/yoken.66.249

20. Sethi SK, Singh S, Dhatwalia SK, Yadav R, Mewara A, Singh M, et al. Evaluation of In- House Loop- Mediated Isothermal Amplification (LAMP) Assay for Rapid Diagnosis of M. tuberculosis in Pulmonary Specimens. J Clin Lab Anal. 2013;27: 272–276. doi:10.1002/jcla.21596

21. Rafati A, Gill P. Microfluidic method for rapid turbidimetric detection of the DNA of Mycobacterium tuberculosis using loop-mediated isothermal amplification in capillary tubes. Microchim Acta. 2015;182: 523–530. doi:10.1007/s00604-014-1354-y

22. Poudel A, Pandey BD, Lekhak B, Rijal B, Sapkota BR, Suzuki Y. Clinical profiling and use of loop-mediated isothermal amplification assay for rapid detection of Mycobacterium tuberculosis from sputum. Kathmandu Univ Med J KUMJ. 2009;7: 109–114. doi:10.3126/kumj.v7i2.2701

23. Lee M-F, Chen Y-H, Peng C-F. Evaluation of reverse transcription loop-mediated isothermal amplification in conjunction with ELISA-hybridization assay for molecular

Formatted: Font: (Default) Times New Roman, 12 pt

detection of Mycobacterium tuberculosis. J Microbiol Methods. 2009;76: 174–180. doi:10.1016/j.mimet.2008.10.005

24. Li Y, Shi L, Pan A, Cao W, Chen X, Meng H, et al. Evaluation of real-time loop-mediated isothermal amplification (RealAmp) for rapid detection of Mycobacterium tuberculosis from sputum samples. J Microbiol Methods. 2014;104: 55–58. doi:10.1016/j.mimet.2014.06.011

25. Kohan L, Shahhosseiny MH, Razavi MR, Parivar K, Moslemi E, Werngren J. Evaluation of loop mediated isothermal amplification for diagnosis of *Mycobacterium tuberculosis* complex in clinical samples. Afr J Biotechnol. 2011;10: 5096–5101. doi:10.4314/ajb.v10i26.

26. Pandey BD, Poudel A, Yoda T, Tamaru A, Oda N, Fukushima Y, et al. Development of an in-house loop-mediated isothermal amplification (LAMP) assay for detection of Mycobacterium tuberculosis and evaluation in sputum samples of Nepalese patients. J Med Microbiol. 2008;57: 439–443. doi:10.1099/jmm.0.47499-0

27. Pathak D, Chakravorty S, Hanif M, Tyagi JS. Lysis of tubercle bacilli in fresh and stored sputum specimens: implications for diagnosing tuberculosis in stored and paucibacillary specimens by PCR. BMC Microbiol. 2007;7: 83. doi:10.1186/1471-2180-7-83

28. Shea YR, Davis JL, Huang L, Kovacs JA, Masur H, Mulindwa F, et al. High Sensitivity and Specificity of Acid-Fast Microscopy for Diagnosis of Pulmonary Tuberculosis in an African Population with a High Prevalence of Human Immunodeficiency Virus. J Clin Microbiol. 2009;47: 1553–1555. doi:10.1128/JCM.00348-09

29. Pham TH, Peter J, Mello FCQ, Parraga T, Lan NTN, Nabeta P, et al. Performance of the TB-LAMP diagnostic assay in reference laboratories: Results from a multicentre study. Int J Infect Dis IJID Off Publ Int Soc Infect Dis. 2018;68: 44–49. doi:10.1016/j.ijid.2018.01.005

30. Gray CM, Katamba A, Narang P, Giraldo J, Zamudio C, Joloba M, et al. Feasibility and Operational Performance of Tuberculosis Detection by Loop-Mediated Isothermal Amplification Platform in Decentralized Settings: Results from a Multicenter Study. J Clin Microbiol. 2016;54: 1984–1991. doi:10.1128/JCM.03036-15

31. Pai M, Flores LL, Pai N, Hubbard A, Riley LW, Colford JM. Diagnostic accuracy of nucleic acid amplification tests for tuberculous meningitis: a systematic review and meta-analysis. Lancet Infect Dis. 2003;3: 633–643. doi:10.1016/s1473-3099(03)00772-2

32. Geojith G, Dhanasekaran S, Chandran SP, Kenneth J. Efficacy of loop mediated isothermal amplification (LAMP) assay for the laboratory identification of Mycobacterium tuberculosis isolates in a resource limited setting. J Microbiol Methods. 2011;84: 71–73. doi:10.1016/j.mimet.2010.10.015

33. Shi J, Dong W, Ma Y, Liang Q, Shang Y, Wang F, et al. GeneXpert MTB/RIF Outperforms Mycobacterial Culture in Detecting Mycobacterium tuberculosis from

Salivary Sputum. In: BioMed Research International [Internet]. 2018 [cited 12 Dec 2019]. Available: https://www.hindawi.com/journals/bmri/2018/1514381/

34. Ho J, Marks GB, Fox GJ. The impact of sputum quality on tuberculosis diagnosis: a systematic review. Int J Tuberc Lung Dis Off J Int Union Tuberc Lung Dis. 2015;19: 537–544. doi:10.5588/ijtld.14.0798

35. Yoon SH, Lee NK, Yim JJ. Impact of sputum gross appearance and volume on smear positivity of pulmonary tuberculosis: a prospective cohort study. BMC Infect Dis. 2012;12: 172. doi:10.1186/1471-2334-12-172

36. Hirooka T, Higuchi T, Tanaka N, Ogura T. [The value of proper sputum collection instruction in detection of acid-fast bacillus]. Kekkaku. 2004;79: 33–37.

37. Sicsú AN, Salem JI, Fujimoto LBM, Gonzales RIC, Cardoso M do S de L, Palha PF. Educational intervention for collecting sputum for tuberculosis: a quasi-experimental study 1. Rev Lat Am Enfermagem. 2016;24. doi:10.1590/1518-8345.0363.2703

38. Cudahy P, Shenoi S. Diagnostics for pulmonary tuberculosis. Postgrad Med J. 2016;92: 187–193. doi:10.1136/postgradmedj-2015-133278

39. Kadioglu EE, Ucar EY, Araz O, Aktas E, Saglam L. A Comparison of Two Different Culture Methods for Use in the Diagnosis of Pulmonary Tuberculosis. Eurasian J Med. 2014;46: 74–77. doi:10.5152/eajm.2014.19

40. Kaku T, Minamoto F, D'Meza R, Morose W, Boncy J, Bijou J, et al. Accuracy of LAMP-TB Method for Diagnosing Tuberculosis in Haiti. Jpn J Infect Dis. 2016;69: 488–492. doi:10.7883/yoken.JJID.2015.519

1. World Health Organization. Global Tuberculosis Report 2018. World Health Organization; 2018.

2. Uplekar M, Weil D, Lonnroth K, Jaramillo E, Lienhardt C, Dias HM, et al. WHO's new end TB strategy. Lancet Lond Engl. 2015;385: 1799–1801. doi:10.1016/S0140-6736(15)60570-0

3. Parsons LM, Somoskövi Á, Gutierrez C, Lee E, Paramasivan CN, Abimiku A, et al. Laboratory Diagnosis of Tuberculosis in Resource-Poor Countries: Challenges and Opportunities. Clin Microbiol Rev. 2011;24: 314. doi:10.1128/CMR.00059-10

Formatted: Font: 12 pt

4. Kim C-K, Cho EA, Shin DM, Choi SW, Shin SY. Comparative Evaluation of the Loop-Mediated Isothermal Amplification Assay for Detecting Pulmonary Tuberculosis. Ann Lab Med. 2018;38: 119–124. doi:10.3343/alm.2018.38.2.119

5. Eddabra R, Ait Benhassou H. Rapid molecular assays for detection of tuberculosis. Pneumonia. 2018;10. doi:10.1186/s41479-018-0049-2

6. Shete PB, Farr K, Strnad L, Gray CM, Cattamanchi A. Diagnostic accuracy of TB-LAMP for pulmonary tuberculosis: a systematic review and meta-analysis. BMC Infect Dis. 2019;19: 268. doi:10.1186/s12879-019-3881-y

7. Notomi T, Okayama H, Masubuchi H, Yonekawa T, Watanabe K, Amino N, et al. Loop-mediated isothermal amplification of DNA. Nucleic Acids Res. 2000;28: e63.

8. Yan L, Xiao H, Zhang Q. Systematic review: Comparison of Xpert MTB/RIF, LAMP and SAT methods for the diagnosis of pulmonary tuberculosis. Tuberc Edinb Scotl. 2016;96: 75–86. doi:10.1016/j.tube.2015.11.005

9. The Use of Loop-Mediated Isothermal Amplification (TB-LAMP) for the Diagnosis of Pulmonary Tuberculosis: Policy Guidance. Geneva: World Health Organization; 2016. Available: http://www.ncbi.nlm.nih.gov/books/NBK384520/

10. Sohn H, Puri L, Nguyen NAT, Van't Hoog AH, Nguyen VAT, Nliwasa M, et al. Cost and affordability analysis of TB-LAMP and Xpert MTB/RIF assays as routine diagnostic tests in peripheral laboratories in Malawi and Vietnam. J Glob Health Sci. 2019;1: e22. doi:10.35500/jghs.2019.1.e22

11. Pandey BD, Poudel A, Yoda T, Tamaru A, Oda N, Fukushima Y, et al. Development of an in-house loop-mediated isothermal amplification (LAMP) assay for detection of

Mycobacterium tuberculosis and evaluation in sputum samples of Nepalese patients. J Med Microbiol. 2008;57: 439–443. doi:10.1099/jmm.0.47499-0

12. George G, Mony P, Kenneth J. Comparison of the Efficacies of Loop-Mediated Isothermal Amplification, Fluorescence Smear Microscopy and Culture for the Diagnosis of Tuberculosis. PLoS ONE. 2011;6. doi:10.1371/journal.pone.0021007

13. Rudeeaneksin J, Bunchoo S, Srisungngam S, Sawanpanyalert P, Chamnangrom S, Kamolwat A, et al. Rapid identification of Mycobacterium tuberculosis in BACTEC MGIT960 cultures by in-house loop-medicated isothermal amplification. Jpn J Infect Dis. 2012;65: 306–311. doi:10.7883/yoken.65.306

14. Nagai K, Horita N, Yamamoto M, Tsukahara T, Nagakura H, Tashiro K, et al. Diagnostic test accuracy of loop-mediated isothermal amplification assay for Mycobacterium tuberculosis: systematic review and meta-analysis. Sci Rep. 2016;6: 39090. doi:10.1038/srep39090

15. Phetsuksiri B, Rudeeaneksin J, Srisungngam S, Bunchoo S, Klayut W, Nakajima C, et al. Comparison of Loop-Mediated Isothermal Amplification, Microscopy, Culture, and PCR for Diagnosis of Pulmonary Tuberculosis. Jpn J Infect Dis. 2020;advpub. doi:10.7883/yoken.JJID.2019.335

16. Kent PT. Public Health Mycobacteriology: A Guide for the Level III Laboratory. U.S. Department of Health and Human Services, Public Health Service, Centers for Disease Control; 1985.

17. Phetsuksiri B, Rudeeaneksin J, Srisungngam S, Bunchoo S, Roienthong D, Mukai T, et al. Applicability of in-house loop-mediated isothermal amplification for rapid

identification of Mycobacterium tuberculosis complex grown on solid media. Jpn J Infect Dis. 2013;66: 249–251. doi:10.7883/yoken.66.249

18. Rudeeaneksin J. Rapid Detection of Mycobacterium Tuberculosis Complex in Sputum and in Culture Growth by LAMP Test. J Health Sci – วารสารวิชาการสาธารณสุข. 2017;19: 300–310.

19. Rafati A, Gill P. Microfluidic method for rapid turbidimetric detection of the DNA of Mycobacterium tuberculosis using loop-mediated isothermal amplification in capillary tubes. Microchim Acta. 2015;182: 523–530. doi:10.1007/s00604-014-1354-y

20. Poudel A, Pandey BD, Lekhak B, Rijal B, Sapkota BR, Suzuki Y. Clinical profiling and use of loop-mediated isothermal amplification assay for rapid detection of Mycobacterium tuberculosis from sputum. Kathmandu Univ Med J KUMJ. 2009;7: 109–114. doi:10.3126/kumj.v7i2.2701

21. Sethi SK, Singh S, Dhatwalia SK, Yadav R, Mewara A, Singh M, et al. Evaluation of In- House Loop- Mediated Isothermal Amplification (LAMP) Assay for Rapid Diagnosis of M. tuberculosis in Pulmonary Specimens. J Clin Lab Anal. 2013;27: 272–276. doi:10.1002/jcla.21596

22. Li Y, Shi L, Pan A, Cao W, Chen X, Meng H, et al. Evaluation of real-time loop-mediated isothermal amplification (RealAmp) for rapid detection of Mycobacterium tuberculosis from sputum samples. J Microbiol Methods. 2014;104: 55–58. doi:10.1016/j.mimet.2014.06.011

23. Lee M-F, Chen Y-H, Peng C-F. Evaluation of reverse transcription loop-mediated isothermal amplification in conjunction with ELISA-hybridization assay for molecular

Formatted: Font: 12 pt

Formatted: Font: 12 pt

detection of Mycobacterium tuberculosis. J Microbiol Methods. 2009;76: 174–180. doi:10.1016/j.mimet.2008.10.005

24. Kohan L, Shahhosseiny MH, Razavi MR, Parivar K, Moslemi E, Werngren J. Evaluation of loop mediated isothermal amplification for diagnosis of *Mycobacterium tuberculosis* complex in clinical samples. Afr J Biotechnol. 2011;10: 5096–5101. doi:10.4314/ajb.v10i26.

25. Habeenzu C, Nakajima C, Solo E, Bwalya P, Kajino K, Miller M, et al. Evaluation of in-house loop-mediated isothermal amplification for tuberculosis diagnosis compared with Xpert MTB/RIF. J Infect Dev Ctries. 2017;11: 440–444. doi:10.3855/jidc.7730

26. Pandey BD, Poudel A, Yoda T, Tamaru A, Oda N, Fukushima Y, et al. Development of an in-house loop-mediated isothermal amplification (LAMP) assay for detection of Mycobacterium tuberculosis and evaluation in sputum samples of Nepalese patients. J Med Microbiol. 2008;57: 439–443. doi:10.1099/jmm.0.47499-0

27. Pathak D, Chakravorty S, Hanif M, Tyagi JS. Lysis of tubercle bacilli in fresh and stored sputum specimens: implications for diagnosing tuberculosis in stored and paucibacillary specimens by PCR. BMC Microbiol. 2007;7: 83. doi:10.1186/1471-2180-7-83

28. Shea YR, Davis JL, Huang L, Kovacs JA, Masur H, Mulindwa F, et al. High Sensitivity and Specificity of Acid-Fast Microscopy for Diagnosis of Pulmonary Tuberculosis in an African Population with a High Prevalence of Human Immunodeficiency Virus. J Clin Microbiol. 2009;47: 1553–1555. doi:10.1128/JCM.00348-09

29. Pham TH, Peter J, Mello FCQ, Parraga T, Lan NTN, Nabeta P, et al. Performance of the TB-LAMP diagnostic assay in reference laboratories: Results from a multicentre study.

Int J Infect Dis IJID Off Publ Int Soc Infect Dis. 2018;68: 44–49. doi:10.1016/j.ijid.2018.01.005

30. Gray CM, Katamba A, Narang P, Giraldo J, Zamudio C, Joloba M, et al. Feasibility and Operational Performance of Tuberculosis Detection by Loop-Mediated Isothermal Amplification Platform in Decentralized Settings: Results from a Multicenter Study. J Clin Microbiol. 2016;54: 1984–1991. doi:10.1128/JCM.03036-15

31. Pai M, Flores LL, Pai N, Hubbard A, Riley LW, Colford JM. Diagnostic accuracy of nucleic acid amplification tests for tuberculous meningitis: a systematic review and meta-analysis. Lancet Infect Dis. 2003;3: 633–643. doi:10.1016/s1473-3099(03)00772-2

32. Shi J, Dong W, Ma Y, Liang Q, Shang Y, Wang F, et al. GeneXpert MTB/RIF Outperforms Mycobacterial Culture in Detecting Mycobacterium tuberculosis from Salivary Sputum. In: BioMed Research International [Internet]. 2018 [cited 12 Dec 2019]. Available: https://www.hindawi.com/journals/bmri/2018/1514381/

33. Ho J, Marks GB, Fox GJ. The impact of sputum quality on tuberculosis diagnosis: a systematic review. Int J Tuberc Lung Dis Off J Int Union Tuberc Lung Dis. 2015;19: 537–544. doi:10.5588/ijtld.14.0798

34. Yoon SH, Lee NK, Yim JJ. Impact of sputum gross appearance and volume on smear positivity of pulmonary tuberculosis: a prospective cohort study. BMC Infect Dis. 2012;12: 172. doi:10.1186/1471-2334-12-172

35. Hirooka T, Higuchi T, Tanaka N, Ogura T. [The value of proper sputum collection instruction in detection of acid-fast bacillus]. Kekkaku. 2004;79: 33–37.

36. Sicsú AN, Salem JI, Fujimoto LBM, Gonzales RIC, Cardoso M do S de L, Palha PF. Educational intervention for collecting sputum for tuberculosis: a quasi-experimental study 1. Rev Lat Am Enfermagem. 2016;24. doi:10.1590/1518-8345.0363.2703

37. Cudahy P, Shenoi S. Diagnostics for pulmonary tuberculosis. Postgrad Med J. 2016;92: 187–193. doi:10.1136/postgradmedj-2015-133278

38. Kadioglu EE, Ucar EY, Araz O, Aktas E, Saglam L. A Comparison of Two Different Culture Methods for Use in the Diagnosis of Pulmonary Tuberculosis. Eurasian J Med. 2014;46: 74–77. doi:10.5152/eajm.2014.19

39. Kaku T, Minamoto F, D'Meza R, Morose W, Boney J, Bijou J, et al. Accuracy of LAMP-TB Method for Diagnosing Tuberculosis in Haiti. Jpn J Infect Dis. 2016;69: 488–492. doi:10.7883/yoken.JJID.2015.519

Supporting information

S1 Table. Review on diagnostic accuracy of in-house LAMP assays for diagnosis of pulmonary tuberculosis (DOCX)

S2 Table. LAMP minimal dataset (CSV)

Response to Reviewers

We want to thank both the editor and the reviewers for granting the opportunity to revise our manuscript for publication in the PLOS one journal. We hope that our responses and revisions would substantially improve the quality of our manuscript and would be qualified for publication in the journal. If there were any further questions or minor points to be addressed or elaborated, please let us know. We would be more than eager to make any further revision.

Editor's comments

Specific comments:

1. Abstract and Tables: Change the format 95%CI to be consistent with the rest of the manuscript, for example "95%CI 78.3,97.5" should be "95%CI 78.3-97.5".
    - Changed as suggested.

2. Line 80, implicated has negative meaning, suggest changing to implemented.
    - We modified the first two sentences as "As financial resources are usually limited in countries with high TB prevalence, a commercial TB-LAMP could still be unattainable. More affordable in-house LAMP assays were later developed and applied in several centers".

3. Line 162 change M. *tuberculosis to Mycobacterium tuberculosis* since this is the first time you mentioned the bacteria. Also, all the "M." need to be italicized in M. *tuberculosis* throughout the manuscript.
    - Corrected as suggested.

4. Line 192 – 193, should the "smear-positive and culture-positive results" be "smear-positive and culture-negative results"?
    - Corrected as suggested.

5. Line 243 – 246: These sentences need reference(s)
    - We inserted some references to the two sentences as suggested.

Reviewer's comments

Thank you to the authors for the revisions made. This is a much better paper to present what is important work. However, I still have a few concerns. These focus on clarification of the 'in-house assay' and the discussion. Additionally, I think a review of the paper by a medical writer or any strong English editor would boost the communication of the results enormously.

1. The paper needs to be reviewed in detail for grammar and English. Other than general tidiness, in a number of places, the intent of what the authors are saying is lost due to odd grammar choices. For the best readability and better reach for the research contained, a review of the writing is recommended. I have made a few notes and suggestions in specific places.
    a. We corrected all of your English suggestions.
    b. We also modified and re-written some of the sentences in the manuscript to improve the readability.
2. The difference in assays still needs to be clearer. An 'in-house assay' is one that is not performed from a kit. You refer to 'the in-house' LAMP assay a lot as if there is only one,

which is not the case. There are many papers out there with different ‘in-house’ LAMP assays. From the introduction, it sounds like you are presenting the findings from an in-house assay you developed following the protocol presented in Pandey et al. If so, this needs to be stated very clearly. However, from the methods section, it does not necessarily sound like you are not following that protocol and that this is a unique in-house assay. Please clarify in the paper.

    a. We made the modification and improved the clarity of our in-house LAMP method as suggested.

3. When discussing previous results and meta-analyses, it needs to be clear that these refer to ‘in-house LAMP assays’ and not ‘the in-house LAMP assay’ as they are not uniform.
    a. Corrected as suggested.
4. Inclusion of ‘Accuracy’ in Table 2 is a bit odd, but it can be kept if it is defined in the statistical methods section.
    a. It was pre-specified in the methods section.
5. The discussion has a lengthy discourse on the costs of Xpert vs LAMP. But there is no referencing of the studies that have costed these two in order to make a proper comparison. It feels quite unsupported.
    a. We removed unsupported statements from the paragraph and make the paragraph more concise.
6. In the discussion, the authors state ‘No previous study had officially addressed the effect of sputum quality on the LAMP test’. I’m not sure this is true and would caution the authors not to make such a sweeping statement.
    a. We removed the sentence out of the discussion section as suggested.
7. In the discussion, “Interestingly, it was revealed from our data that the proportion of smear-positive, LAMP-positive results was also significantly lower in salivary sputum than in mucous sputum (31.3% vs. 57.5%, p=0.009 and 29.9% vs. 60.0%, p=0.003, respectively). Therefore, the sensitivity and accuracy of all tests, including LAMP, might be underestimated.” 1) Do not present new results in the discussion – these need to be included in the Results section first. 3) are these sensivity? Specificity? Accuracy? 3) This is not an interpretation that makes sense. The sensitivity/specificity is reported based on the best sputum sample available from the patients – quality samples are difficult to obtain. You can instead interpret it as ‘Sensitivity and specificity would be improved if higher quality sputum is obtained’.
    a. We modified the content as suggested.
    b. We moved the findings to the results section.
8. In general, the discussion needs to be revised to make only statements supported by the literature, the study, or a comparison of the two. Much of the discussion feels like the authors musings.
    a. We modified the whole discussion sections to be as objective as possible.
9. In the discussion, I would suggest focusing on sensitivity and specificity and not accuracy as accuracy is not a common way of discussing or assessing diagnostic tests due to its difficulty of interpretation.
    a. Corrected as suggested.
10. The references as displayed in the reference section aren’t quite right. In reference #1, instead of Lancet, the Journal is listed as Lancet Lond Engl which is not correct. This inclusion of a city occurs in reference #7 as well.
    a. Corrected as suggested.
