# Supplementary material for: Pragmatic accuracy of an in-house loop-mediated isothermal amplification (LAMP) for diagnosis of pulmonary tuberculosis in a Thai community hospital
Source: PLoS One. 2020 Jul 23;15(7):e0236496. doi: 10.1371/journal.pone.0236496 (PMC7377475; doi:10.1371/journal.pone.0236496)
Supplement: Attachment — Submitted filename: Response to Reviwers.docx [file pone.0236496.s003.docx]

**Responses to Reviewers’ comments**

**Pragmatic accuracy of loop-mediated isothermal amplification (LAMP)**

**for diagnosis of pulmonary tuberculosis in a Thai community hospital**

**Reviewer #1:**

1. The study aims to evaluate usefulness of a LAMP method in a practical setting in Thailand. The LAMP method is now available as an only commercial kit TB-LAMP assay (Loopamp™MTBC Detection Kit, Eiken Chemical Company Ltd., Japan) as endorsed by WHO in 2016. It seems that the method used in this study is a unique system at least partially. So, it is important to state explicitly that the target to be evaluated was an in-house LAMP and not one commercially available LAMP recommended by WHO.
   - The LAMP test in our study was a non-commercial, in-house LAMP.
   - We re-wrote the manuscript and emphasized that the test used was in-house LAMP.
2. In evaluating the sensitivity of the method, the authors used culture negative (clinically defined) cases, as well as bacteriologically confirmed cases, as a gold standard of the cases of TB. It may be difficult to admit the clinical diagnosis as a diagnostic basis for such a study as this, apart from clinical practice. Vice versa, the definition of the gold (conventional) standard for specificity (non-cases) should be reconsidered. The following paper may be of use in revising the paper; Kaku et al: Accuracy of LAMP-TB Method for Diagnosing Tuberculosis in Haiti. Jpn. J. Infect. Dis., 69, 488–492, 2016.
   - We modified the inclusion criteria for analysis as suggested by both reviewers.
   - As the analysis was done in a per-patient fashion, patients with smear-positive and culture-negative results would be excluded, as these patients were considered as probable TB cases. Therefore, the evaluation of sensitivity would include patients with both smear positive and smear negative with positive culture results. In contrast, the evaluation of specificity would include only patients with smear-negative and culture-negative results.

**Reviewer #2:**

1. Abstract/Background: “proven diagnostic performance” – this is both vague and too specific at the same time, “most of the results were validated” – the results aren’t validated, the assay is validated
   - We rewrote the abstract and introduction part as suggested.
2. The language surrounding people with possible TB needs to be updated throughout the paper - avoid the use of terms like "TB suspects" that increase the stigma surrounding this disease. <http://www.stoptb.org/assets/documents/resources/publications/acsm/LanguageGuide_ForWeb20131110.pdf>
   - We rewrote the abstract and introduction part as suggested.
3. The paper states repeatedly that there is little work published from resource-challenged settings, but this claim is not supported. Even the references given cite studies in such decentralized settings. Maybe it just hasn’t been done in Thailand? A better summary of the literature needs to be included. How does this compare to other studies? How is the TB LAMP test performed in this study compare to the TB LAMP tests in other published literature? A better focus on properly relating the current study to the body of work in the literature rather than trying to claim it is quite novel would actually strengthen the paper. There is merit in replication or demonstrating an important diagnostic in a new geographical area.
   - We rewrote the abstract and introduction part as suggested.
4. In-house vs commercialized kit is mentioned but not explained. And the position of this paper (what LAMP testing approach is used) is not properly placed in the context of what other papers are using and the potential impact on sensitivity/specificity.
   - We rewrote the abstract and introduction part as suggested.
5. The sensitivity/specificity of LAMP in other papers, settings, etc needs to be stated with numbers and not just alluded to. A proper, specific summary of the literature is lacking.
   - We rewrote the abstract and introduction part as suggested.
6. “In 2016, WHO suggested the use of LAMP assay for the diagnosis of pulmonary tuberculosis” – this is not quite right, WHO recommendations are very specific and it is important to get that right. From the abstract of the citation provided: “WHO recommends that TB-LAMP can be used as a replacement for microscopy for the diagnosis of pulmonary TB in adults with signs and symptoms of TB”. This needs to be stated correctly. Also, given the paper has mentioned in-house vs commercialized kits, it needs to be clarified that the WHO guidance refers only to the Eiken LAMP kit.
   - We rewrote the abstract and introduction part as suggested.
7. “LAMP assay has a low cost per test, does not required advanced technological facilities, and can be routinely practiced in general hospital laboratories [3].” Reference 3 doesn’t support this statement – it doesn’t say anywhere that the LAMP assay has a low cost per test. It says “Costs can be kept to a minimum if testing is limited to specimens from the most high-risk patients based on proper clinical assessments and national testing algorithms based on public health policies.” There are other publications on the cost of the LAMP assay for TB diagnosis. The authors might explain better the infrastructure/training needed for LAMP based on this reference and others.
   - We rewrote the abstract and introduction part as suggested.
   - We changed the references to the statement as follow: Sohn H. Cost, affordability, and cost-effectiveness of TB-LAMP assay. In: Report to WHO Guideline Development Group Meeting on TB-LAMP Assay. Edn. Geneva: World Health Organization; 2016 and Shete PB, Farr K, Strnad L, Gray CM, Cattamanchi A. Diagnostic accuracy of TB-LAMP for pulmonary tuberculosis: a systematic review and meta-analysis. BMC Infect Dis. 2019;19(1):268. Published 2019 Mar 19. doi:10.1186/s12879-019-3881-y
8. Reference 5 doesn’t appear to really relate to the sentences it comes after. Reference 3 would make a lot more sense as it is a detailed overview of TB diagnostics including many molecular diagnostics.
   - We rewrote the abstract and introduction part as suggested.

**Setting**

1. The paper needs to do more to state what sets this setting apart from (or ties it to) other studies. See the methods section describing setting in reference 22 for how attributes of the specific site can be expressed in the context of the needs of LAMP.
   - We elaborated the character of our setting as suggested:
   - Level of health system: rural
   - Distance to reference laboratory: 0 km
   - Median LAMP test workload: 6 (4-10)
   - Electricity and backup power: infrequent power outages, power generator (350 Kw) and UPS (2.7 Kw)
   - Biosafety cabinet infrastructure: BSC class II
   - Laboratory staff: 4 lab technicians, 1 lab assistant
2. Study Design: This is not a cross-sectional design; it is a prospective design. The plan was to prospectively enroll 120 patients.
   - We changed the type of design to prospective diagnostic accuracy study as suggested.
   - We would like to make a constructive argument on this point, as the diagnostic accuracy research is actually cross-sectional study in design. The cross-sectional design is only the type of membership condition, single component of study base, and cross-sectional design can therefore be collected prospectively or retrospectively. We would like to ask you to kindly refer to this reference: Assessment of the accuracy of diagnostic tests: the cross-sectional study by Knottnerus JA, 2003.
     Link: https://www.ncbi.nlm.nih.gov/pubmed/14615003
3. “New patients who were clinically suspected of 109 pulmonary TB (coughing for more than two weeks with or without hemoptysis), aged more than 18 years old were consecutively invited into the study regardless of nation status.” Suggest re-writing to something more like: ‘Adults more than 18yrs of age with symptoms indicative of pulmonary TB (coughing…) and no history of TB were consecutively enrolled regardless of national status.’ If patients were ‘invited’ but not enrolled, we need numbers on how many declined.
   - We re-wrote the sentence as suggested: Adult patients aged more than 15 years old with symptoms indicative of pulmonary TB (coughing for more than two weeks with or without hemoptysis) and no history of TB were consecutively enrolled regardless of national status.
4. “Samples with contaminated culture results or samples from patients who were previously documented as TB cases were excluded.” Were the patients excluded or the samples?
   - Patients with previously documented TB cases were excluded.
   - Patients with two contaminated or missing culture results were excluded.

**Methods**

1. A map of which samples were used for what tests would be quite helpful. Highlight if any of the reference tests (smear, LJ culture, MGIT culture) were performed on the same sputum as LAMP.
   - Conventional macroscopy, LAMP test, and culture were conducted as routinely done.
   - All patients were given three sealed containers for the collection of morning sputum specimens. Of all containers sent to the laboratory, only the one with seemingly adequate sputum, containing both mucoid or mucopurulent characters with a sample volume more than 3 ml, was used for the whole investigation procedures as routinely done. Specimens were sent for smear microscopy with conventional acid-fast bacilli (AFB) staining with Ziehl-Neelsen technique and fluorescence acid-fast staining with Auramine O solution.
2. Make it clear somewhere that smear-negative refers to AFB smear-negative.
   - We added detail on the smear-negative status as suggested.
   - According to WHO definitions, any patient with at least two AFB smears of scanty grade or one or more smears of 1+ or more was defined as smear-positive case. Smear-negative case was conversely defined.
3. Study size estimation
   This has no purpose here – the study is done. Sample size estimation is for study planning purposes, for securing funding and making sure the plan has statistical validity.
   - The study size estimation part was removed as suggested.
4. Statistical analysis. The first four sentences are unnecessary.
   - The first four sentences were removed as suggested.
5. The authors need to state what method was used to obtain the 95% CI for the sens/spec/PPV/NPV/LR+. It is clear from my testing that the Clopper Pearson binomial exact test was used, the authors should include the reference (usually found in the software documentation).
   - The 95% confidence intervals were calculated using the Clopper Pearson binomial exact method.
   - We added this statement in the statistical section and added the citation as suggested.
6. Kappa statistics are for inter-reader reliability, not for comparison of correlations between tests. It includes the concept that agreement may happen by chance when two people are guessing. However, it is not appropriate for comparison of diagnostic results because there isn’t guessing – the samples should not agree by chance but because they are or are not TB and the sensitivities of tests objectively vary. Spearman’s correlation can be used, but I think what you actually want is McNemar’s test. The desire is to compare the diagnostic performance (i.e. accuracy) between tests – McNemar’s test will do that. Alternatively, Spearman’s correlation can look at the [objective] agreement between tests.
   - Spearman’s rank correlation was inserted into the manuscript to represent the objective agreement between tests as suggested.
   - The agreement of LAMP test with smear microscopy methods was analyzed with Kappa’s statistics and Spearman’s rank correlation.
   - We still presented the value of Kappa’s statistics as many of the previous studies on LAMP assay and other diagnostic tests had done [1–3].

**Results**

1. Table 1 is dedicated to showing the patient clinical characteristics by culture status. The p-values shown test whether these characteristics differ significantly dependent on culture status. It is expected that gender, nationality, and age should not differ. Whereas it is also expected that chest x-rays and sputum quality would differ. The baseline demographic data between culture188 positive and negative patients were comparable except for the presence of cavitary lesions on 189 chest radiographs and the character of collected sputum (Table 1). Age, nationality, and gender are demographic data. Chest x-ray and sputum quality are clinical characteristics.
   - We reanalyzed all the data after exclusion of patients with probable TB (LAMP test positive and AFB smear positive patients with negative culture).
   - All the baseline demographic and clinical characteristics data were reanalyzed and presented in Table 1.
   - The statements in the results section were re-written as suggested.
2. Table 2 – re-check the NPV for parallel testing
   - We reanalyzed all the data after exclusion of patients with probable TB (LAMP test positive and AFB smear positive patients with negative culture).
   - All the data on Table 2 were checked for any error as suggested.
3. There are a lot of LAMP-positive and AFB smear-positive patients with negative culture. Especially given that the tests are done on different sputum samples, these should be considered patients with probable TB and not used in assessing sensitivity and specificity.
   - We reanalyzed all the data after exclusion of patients with probable TB (LAMP test positive and AFB smear positive patients with negative culture).
   - The final study size for analysis of LAMP test diagnostic accuracy was therefore 107 patients. (8 patients were excluded, 6 patients with both LAMP test and AFB smear-positive and culture negative, 1 patient with AFB positive and culture negative, and 1 patient with fluorescence stain positive and culture negative)
4. There are too few smear-negative, culture-positive patients to assess sensitivity. Specificity should not be stratified by smear status, only sensitivity. For the reason above (that smear-positive, culture-negative patients shouldn’t be included in estimations of sensitivity/specificity of LAMP), what the paper is calling ‘smear-negative specificity’ should in fact be reported as the actual specificity of LAMP.
   - We exclude smear-positive, culture negative patients from the analysis as suggested.
   - We reported the actual specificity of LAMP test without stratification.
   - We acknowledged that our there are too few smear negative, culture positive patients to assess sensitivity in the discussion part.
5. Table 2 – the p-values shown have no real meaning! If you want to compare accuracy of tests, you cannot do a p-value over the final accuracy measures among a bunch of tests. You need to compare tests 1 against another by using 2x2 grids and McNemar’s test. So, if you want to compare the accuracy of LAMP to the accuracy of AFB stain, you use the grid in Table 3 and McNemar’s test:
   - The comparison of diagnostic indices between LAMP test and AFB, fluorescence stain was re-analyzed using McNemar’s exact probability test as suggested. We presented the result of the pairwise tests separately and reformatted Table 2.
   - Pairwise testing was not performed to compare the specificity between the LAMP test and the smear microscopy methods as the specificity of the latter was affected by incorporation bias and would not be comparable to the in-house LAMP.
   - Table 3 was also reformatted.
   - Spearman’s rank correlation was used as suggested.

**Discussion**

1. “This study had demonstrated the pragmatic performance of the LAMP test, which was comparable to that of the conventional smear microscopy and the fluorescence microscopy.” Not true, the performance of LAMP as evaluated in this study was below that of smear microscopy.
   - We rewrote the discussion part as suggested.
   - “This study had demonstrated the pragmatic diagnostic performance of the in-house LAMP assay in a remote hospital of a high TB burden country. It was revealed that the overall sensitivity of the in-house LAMP in our study was lower than the numbers reported in the majority of the previous in-house LAMP studies. Nonetheless, the specificity was comparable to other figures reported in literature. In comparison to microscopy methods, the AFB and fluorescence stain, the in-house LAMP was found to be inferior in terms of overall sensitivity (82.0% vs. 88.0%, p=0.375) and accuracy (88.8% vs. 94.4%, p=1.000); however, the comparative statistical test revealed non-significant results. Based on the result of our study, we suggest that the in-house LAMP should not be a substitute to conventional smear methods, but should be done in parallel, which would result in a higher sensitivity with fewer false-negative TB cases.”
2. “Although the sensitivity and specificity of the LAMP test were lower than that of the acid-fast stain and the fluorescence stain, the comparative statistical test revealed non-significant results” This is still true when McNemar’s test is performed, but the right statistical tests need to be used in the paper. Furthermore, a non-significant result doesn't mean no difference, it means the difference is likely smaller than the power of the study to detect.
   - We rewrote the discussion part as suggested.
   - We reanalyzed our data using McNemar’s exact probability test as suggested.
3. Put PPV/NPV in the context of the local prevalence of disease! State from the literature or reliable source what the prevalence of TB is in the hospital’s area of Thailand. I would suggest giving the readers an example: Given that prevalence and a group of 1000 patients, state how many would be true positives, false positive, true negatives, and false negatives. You can therefore assess what burden the different accuracies will place on the hospital. I.e. if the specificity is quite low and the sensitivity is higher, is that better? If the sensitivity is high and the specificity is lower, is that better? Relate this to the LR+.
   - We would like to make a constructive argument to this question as follow: The prevalence of culture-positive TB in this study was 46.7%. As this was a “consecutive recruitment of patients with sign and symptoms suggestive of pulmonary TB” or “patients with higher pre-test probability that the general prevalence” or the “person that the in-house LAMP test was intended to be used”, the calculation of positive predictive values could be directly calculated and reported from the study data as in the other study [1]. Moreover, both the in-house LAMP assay and acid-fast stain were not intended to be used as screening tests in the general population. For this reason, we did not include this part in our manuscript; however, we provide the answer to the question in this response paper.
   - The latest Maesot’s population figures from the Health Data Center (HDC), the ministry of public health, Thailand, was 115,108 in 2019. The prevalence of pulmonary tuberculosis was 351 per 100,000 or 35 per 10,000.

|  | TB case | Non-TB case | Total |  |
| --- | --- | --- | --- | --- |
| LAMP positive | 29 | 528 | 557 | PPV 29/557=5.2% |
| LAMP negative | 6 | 9,437 | 9,443 | NPV 9437/9443=94.9% |
| Total | 35 | 9,965 | 10,000 | Prevalence=0.0035 |

4. “In the clinical context of TB diagnosis, both the LAMP test and the smear microscopy are considered as a diagnostic test which would normally be done in TB suspects with high pre-test probability [14]” – this is not what the reference says.
   - The reference states “The TB LAMP assay is usually applied for TB-suspected patients and is rarely used for screening purpose. To rule-in the TB diagnosis, specificity is more important than sensitivity.”
   - What we’re trying to imply from this statement was that the LAMP test was developed to be applied for patients who were suspicious of having TB with “higher pre-test probability than average person”. As the LAMP test was not for screening purpose, specificity is more important and should be more focused than sensitivity.
   - After we re-analyzed the data with the exclusion of probable TB cases, our specificity increased to comparable level with previous studies. The parallel and serial testing was omitted from our analysis as the test accuracy of combination of the in-house LAMP with other smear microscopy methods would be seriously affected by incorporation bias (smear-positive, culture-negative patients were all excluded.
5. “Therefore, a serial test relying on both the result from the LAMP test and the acid-fast stain would be more appropriate for use as a rule-in test as it carried higher specificity and positive likelihood ratio than other methods.” Authors should define ‘rule-in’ test and what is generally expected of such a test. Should note the increased cost of such an approach.
   - After we re-analyzed the data with the exclusion of probable TB cases, our specificity increased to comparable level with previous studies. The parallel and serial testing was omitted from our analysis as the test accuracy of combination of the in-house LAMP with other smear microscopy methods would be seriously affected by incorporation bias (smear-positive, culture-negative patients were all excluded.
6. The effect of a gold standard which is not itself perfect should be discussed. Also the variability between sputum samples should be discussed.
   - The use of routine TB culture as a reference standard might be inadequate, as some TB patients could be classified as not having TB [6]. Different culture media and techniques could be used in composite to achieve different performance characteristics[4]. With a higher quality reference standard, the sensitivity of the in-house LAMP should be increased when a portion of three remaining false-positive cases was re-classified as true-positive cases.
   - This study had a higher proportion of salivary sputum than mucous sputum. This could affect the diagnostic performance of both the index and the reference test[5]. The percentage of culture-positive TB cases was lower in salivary samples than in mucous samples (35.8% vs. 65.0%, p=0.005). Both the quality and quantity of sputum specimens were associated with positivity of smear, molecular testing methods (Xpert MTB/RIF and PCR), and TB culture [6,7]. Thus, it was possible that some patients with pulmonary TB might be classified as smear-negative, LAMP-negative, or even culture-negative cases. Interestingly, it was revealed from our data that the proportion of smear-positive, LAMP-positive results was also significantly lower in salivary sputum than in mucous sputum (31.3% vs 57.5%, p=0.009 and 29.9% vs. 60.0%, p=0.003, respectively). Therefore, the sensitivity and accuracy of all tests, including LAMP, might be underestimated. Previous studies reported that by improving the sputum quality, TB diagnostic yield increased[8,9]. Therefore, high-quality sputum collection must be encouraged both in practice and studies.
7. A better look at the differences between this study and others with better test performance needs to be done.
   - In this study, the sensitivity of the in-house LAMP test was 82.0% (95%CI 68.6-91.4) in culture-positive TB patients, respectively. In the past, several studies had reported a higher sensitivity of the in-house LAMP test, which ranges from 90.0 to 100.0%. Most of these studies were either University hospital, TB-specialized centers or hospitals, or national TB-specialized laboratory, which were generally equipped with highly-trained personnel and adequate infrastructural supports. The overall sensitivity of our in-house LAMP was consistent with two previous studies from India and Zambia, which was 79.5% (95%CI 64.0-89.0) and 81.4% (95%CI 71.6-89.0), respectively. Although both studies were performed in University hospitals, the LAMP procedures were modified to suit local conditions, and sputum processing and DNA extraction was done with commercial kits. The higher sensitivity of the acid-fast stain and the fluorescence stain in our study could be explained by the high prevalence of TB, the absence of HIV patient or a smaller number of patients with paucibacillary sputum, and the availability of skilled technicians
8. “Currently, the WHO only supported the use of two rapid molecular tests for the diagnosis of 294 pulmonary tuberculosis, which were Xpert MTB/RIF and the LAMP test” – as the concept of LAMP test from a kit and other LAMP tests has been raised, and the variability of accuracy depending, it needs to be clear that the WHO recommendation is only for the Eiken LAMP test kit!
   - We edited the statement as follow: “Currently, the WHO only supported the use of two rapid molecular tests for the diagnosis of pulmonary tuberculosis, which were Xpert MTB/RIF and the commercialized TB-LAMP assay”.

**References**

1.	George G, Mony P, Kenneth J. Comparison of the Efficacies of Loop-Mediated Isothermal Amplification, Fluorescence Smear Microscopy and Culture for the Diagnosis of Tuberculosis. PLoS ONE. 2011;6. doi:10.1371/journal.pone.0021007

2.	Phetsuksiri B, Rudeeaneksin J, Srisungngam S, Bunchoo S, Klayut W, Nakajima C, et al. Comparison of Loop-Mediated Isothermal Amplification, Microscopy, Culture, and PCR for Diagnosis of Pulmonary Tuberculosis. Jpn J Infect Dis. 2020;advpub. doi:10.7883/yoken.JJID.2019.335

3.	Wang Z, Sun H, Ren Z, Xue B, Lu J, Zhang H. Feasibility and Performance of Loop-Mediated Isothermal Amplification Assay in the Diagnosis of Pulmonary Tuberculosis in Decentralized Settings in Eastern China. BioMed Res Int. 2019;2019. doi:10.1155/2019/6845756

4.	Cudahy P, Shenoi S. Diagnostics for pulmonary tuberculosis. Postgrad Med J. 2016;92: 187–193. doi:10.1136/postgradmedj-2015-133278

5.	Shi J, Dong W, Ma Y, Liang Q, Shang Y, Wang F, et al. GeneXpert MTB/RIF Outperforms Mycobacterial Culture in Detecting Mycobacterium tuberculosis from Salivary Sputum. In: BioMed Research International [Internet]. 2018 [cited 12 Dec 2019]. Available: https://www.hindawi.com/journals/bmri/2018/1514381/

6.	Ho J, Marks GB, Fox GJ. The impact of sputum quality on tuberculosis diagnosis: a systematic review. Int J Tuberc Lung Dis Off J Int Union Tuberc Lung Dis. 2015;19: 537–544. doi:10.5588/ijtld.14.0798

7.	Yoon SH, Lee NK, Yim JJ. Impact of sputum gross appearance and volume on smear positivity of pulmonary tuberculosis: a prospective cohort study. BMC Infect Dis. 2012;12: 172. doi:10.1186/1471-2334-12-172

8.	Hirooka T, Higuchi T, Tanaka N, Ogura T. [The value of proper sputum collection instruction in detection of acid-fast bacillus]. Kekkaku. 2004;79: 33–37.

9.	Sicsú AN, Salem JI, Fujimoto LBM, Gonzales RIC, Cardoso M do S de L, Palha PF. Educational intervention for collecting sputum for tuberculosis: a quasi-experimental study 1. Rev Lat Am Enfermagem. 2016;24. doi:10.1590/1518-8345.0363.2703
