# Supplementary material for: Pragmatic accuracy of an in-house loop-mediated isothermal amplification (LAMP) for diagnosis of pulmonary tuberculosis in a Thai community hospital
Source: PLoS One. 2020 Jul 23;15(7):e0236496. doi: 10.1371/journal.pone.0236496 (PMC7377475; doi:10.1371/journal.pone.0236496)
Supplement: Attachment — Submitted filename: PONE-D-20-00432_second review.docx [file pone.0236496.s005.docx]

Thank you to the authors for the revisions made. This is a much better paper to present what is important work. However, I still have a few concerns. These focus on clarification of the ‘in-house assay’ and the discussion. Additionally, I think a review of the paper by a medical writer or any strong English editor would boost the communication of the results enormously.

1. The paper needs to be reviewed in detail for grammar and English. Other than general tidiness, in a number of places, the intent of what the authors are saying is lost due to odd grammar choices. For the best readability and better reach for the research contained, a review of the writing is recommended. I have made a few notes and suggestions in specific places.
2. The difference in assays still needs to be clearer. An ‘in-house assay’ is one that is not performed from a kit. You refer to ‘the in-house’ LAMP assay a lot as if there is only one, which is not the case. There are many papers out there with different ‘in-house’ LAMP assays. From the introduction, it sounds like you are presenting the findings from an in-house assay you developed following the protocol presented in Pandey et al. If so, this needs to be stated very clearly. However, from the methods section, it does not necessarily sound like you are not following that protocol and that this is a unique in-house assay. Please clarify in the paper.
3. When discussing previous results and meta-analyses, it needs to be clear that these refer to ‘in-house LAMP assays’ and not ‘the in-house LAMP assay’ as they are not uniform.
4. Inclusion of ‘Accuracy’ in Table 2 is a bit odd, but it can be kept if it is defined in the statistical methods section.
5. The discussion has a lengthy discourse on the costs of Xpert vs LAMP. But there is no referencing of the studies that have costed these two in order to make a proper comparison. It feels quite unsupported.
6. In the discussion, the authors state ‘No previous study had officially addressed the effect of sputum quality on the LAMP test’. I’m not sure this is true and would caution the authors not to make such a sweeping statement.
7. In the discussion, “Interestingly, it was revealed from our data that the proportion of smear-positive, LAMP-positive results was also significantly lower in salivary sputum than in mucous sputum (31.3% vs. 57.5%, p=0.009 and 29.9% vs. 60.0%, p=0.003, respectively). Therefore, the sensitivity and accuracy of all tests, including LAMP, might be underestimated.” 1) Do not present new results in the discussion – these need to be included in the Results section first. 3) are these sensivity? Specificity? Accuracy? 3) This is not an interpretation that makes sense. The sensitivity/specificity is reported based on the best sputum sample available from the patients – quality samples are difficult to obtain. You can instead interpret it as ‘Sensitivity and specificity would be improved if higher quality sputum is obtained’.
8. In general, the discussion needs to be revised to make only statements supported by the literature, the study, or a comparison of the two. Much of the discussion feels like the authors musings.
9. In the discussion, I would suggest focusing on sensitivity and specificity and not accuracy as accuracy is not a common way of discussing or assessing diagnostic tests due to its difficulty of interpretation.
10. The references as displayed in the reference section aren’t quite right. In reference #1, instead of Lancet, the Journal is listed as Lancet Lond Engl which is not correct. This inclusion of a city occurs in reference #7 as well.
