# Supplementary material for: Pragmatic accuracy of an in-house loop-mediated isothermal amplification (LAMP) for diagnosis of pulmonary tuberculosis in a Thai community hospital
Source: PLoS One. 2020 Jul 23;15(7):e0236496. doi: 10.1371/journal.pone.0236496 (PMC7377475; doi:10.1371/journal.pone.0236496)
Supplement: Attachment — Submitted filename: Response to Reviewers.docx [file pone.0236496.s006.docx]

**Response to Reviewers**

We want to thank both the editor and the reviewers for granting the opportunity to revise our manuscript for publication in the PLOS one journal. We hope that our responses and revisions would substantially improve the quality of our manuscript and would be qualified for publication in the journal. If there were any further questions or minor points to be addressed or elaborated, please let us know. We would be more than eager to make any further revision.

**Editor’s comments**

Specific comments:

1. Abstract and Tables:  Change the format 95%CI to be consistent with the rest of the manuscript, for example “95%CI 78.3,97.5” should be “95%CI 78.3-97.5”.
   - Changed as suggested.
2. Line 80, implicated has negative meaning, suggest changing to implemented.
   - We modified the first two sentences as “As financial resources are usually limited in countries with high TB prevalence, a commercial TB-LAMP could still be unattainable. More affordable in-house LAMP assays were later developed and applied in several centers”.
3. Line 162 change M. *tuberculosis to Mycobacterium* *tuberculosis* since this is the first time you mentioned the bacteria*.* Also, all the *“*M.” need to be italicized in M. *tuberculosis* throughout the manuscript*.*
   - Corrected as suggested.
4. Line 192 – 193, should the “smear-positive and culture-positive results” be “smear-positive and culture-negative results”?
   - Corrected as suggested.
5. Line 243 – 246:  These sentences need reference(s)
   - We inserted some references to the two sentences as suggested.

**Reviewer’s comments**

Thank you to the authors for the revisions made. This is a much better paper to present what is important work. However, I still have a few concerns. These focus on clarification of the ‘in-house assay’ and the discussion. Additionally, I think a review of the paper by a medical writer or any strong English editor would boost the communication of the results enormously.

1. The paper needs to be reviewed in detail for grammar and English. Other than general tidiness, in a number of places, the intent of what the authors are saying is lost due to odd grammar choices. For the best readability and better reach for the research contained, a review of the writing is recommended. I have made a few notes and suggestions in specific places.
   a. We corrected all of your English suggestions.
   b. We also modified and re-written some of the sentences in the manuscript to improve the readability.
2. The difference in assays still needs to be clearer. An ‘in-house assay’ is one that is not performed from a kit. You refer to ‘the in-house’ LAMP assay a lot as if there is only one, which is not the case. There are many papers out there with different ‘in-house’ LAMP assays. From the introduction, it sounds like you are presenting the findings from an in-house assay you developed following the protocol presented in Pandey et al. If so, this needs to be stated very clearly. However, from the methods section, it does not necessarily sound like you are not following that protocol and that this is a unique in-house assay. Please clarify in the paper.
   a. We made the modification and improved the clarity of our in-house LAMP method as suggested.
3. When discussing previous results and meta-analyses, it needs to be clear that these refer to ‘in-house LAMP assays’ and not ‘the in-house LAMP assay’ as they are not uniform.
   a. Corrected as suggested.
4. Inclusion of ‘Accuracy’ in Table 2 is a bit odd, but it can be kept if it is defined in the statistical methods section.
   a. It was pre-specified in the methods section.
5. The discussion has a lengthy discourse on the costs of Xpert vs LAMP. But there is no referencing of the studies that have costed these two in order to make a proper comparison. It feels quite unsupported.
   a. We removed unsupported statements from the paragraph and make the paragraph more concise.
6. In the discussion, the authors state ‘No previous study had officially addressed the effect of sputum quality on the LAMP test’. I’m not sure this is true and would caution the authors not to make such a sweeping statement.
   a. We removed the sentence out of the discussion section as suggested.
7. In the discussion, “Interestingly, it was revealed from our data that the proportion of smear-positive, LAMP-positive results was also significantly lower in salivary sputum than in mucous sputum (31.3% vs. 57.5%, p=0.009 and 29.9% vs. 60.0%, p=0.003, respectively). Therefore, the sensitivity and accuracy of all tests, including LAMP, might be underestimated.” 1) Do not present new results in the discussion – these need to be included in the Results section first. 3) are these sensivity? Specificity? Accuracy? 3) This is not an interpretation that makes sense. The sensitivity/specificity is reported based on the best sputum sample available from the patients – quality samples are difficult to obtain. You can instead interpret it as ‘Sensitivity and specificity would be improved if higher quality sputum is obtained’.
   a. We modified the content as suggested.
   b. We moved the findings to the results section.
8. In general, the discussion needs to be revised to make only statements supported by the literature, the study, or a comparison of the two. Much of the discussion feels like the authors musings.
   a. We modified the whole discussion sections to be as objective as possible.
9. In the discussion, I would suggest focusing on sensitivity and specificity and not accuracy as accuracy is not a common way of discussing or assessing diagnostic tests due to its difficulty of interpretation.
   a. Corrected as suggested.
10. The references as displayed in the reference section aren’t quite right. In reference #1, instead of Lancet, the Journal is listed as Lancet Lond Engl which is not correct. This inclusion of a city occurs in reference #7 as well.
    a. Corrected as suggested.
