# Supplementary material for: Pragmatic accuracy of an in-house loop-mediated isothermal amplification (LAMP) for diagnosis of pulmonary tuberculosis in a Thai community hospital
Source: PLoS One. 2020 Jul 23;15(7):e0236496. doi: 10.1371/journal.pone.0236496 (PMC7377475; doi:10.1371/journal.pone.0236496)
Supplement: Attachment — Submitted filename: Response to Reviwers.docx [file pone.0236496.s008.docx]

**Responses to Reviewers’ comments: minor revision II**

**Pragmatic accuracy of an in-house loop-mediated isothermal amplification (LAMP) for diagnosis of pulmonary tuberculosis in a Thai community hospital**

**Reviewer #2:** The manuscript is much improved after the second revision. Some minor problems remain, but these are specific rather than general and should be able to be easily addressed.

- The use of ‘This’ at the beginning of sentences in the Background is not very clear.

Corrected as suggested

- These nucleic amplification techniques were known for yielding rapid and accurate TB diagnosis, which would overcome the limitations of classical methods (ie, insensitivity for smear microscopy and lengthy incubation period for TB culture).

- However, several obstacles remain for the application of these molecular tests as point-of-care testing in community settings due to their complexity to execute and substantial requirements for financial and personnel resources.

- Some of the other corrections have also reduced clarity rather than enhanced, I have made a note for each instance for ease of author correction in the annoted pdf that I have attached to this review.

- Corrected as suggested in the reviewer’s attached pdf file.

- Line 84, ‘More affordable in-house LAMP assays were later developed’ – this is still a problematic assertion as the first reference (the original reference for the sentence) is dated 2008 which is published prior to the availability and endorsement of the commercial TB-LAMP assay being compared. Also, none of the now 5 references compare affordability of the commercialized assay to an in-house assay to the commercial assay. Suggest the authors change to ‘An in-house LAMP assay may be more affordable…”

- Corrected as suggested.
- An in-house LAMP assay may be more affordable to the commercial one [11–15].

- Line 331, the authors state that no patients with HIV infection were included – was HIV testing performed on all patients? Were they confirmed to be HIV negative? Or should this say more simply that HIV testing was not performed?

- All patients with symptoms suggestive of TB were offered routine HIV counselling and HIV rapid antibody tests. (We added this within the methods section)
- All included patients had negative HIV results. (we added this within the results section)

- Line 341, authors should remove ‘Sensitivity and specificity would be improved if higher quality sputum is obtained’ – the authors have no way to confirm that. And, no support has been given as to why specificity would, even hypothetically, change.

- We removed the sentence as suggested.

In the previous review, I noted that if the Kappa statistic 95% CI is displayed, then the Spearman Rho in the same table should also have the 95% CI. However, in retrospect, I realize that Spearman’s Rho should not have a 95% CI as one cannot be calculated for this method. Suggest removing and making a note.

- We removed the 95%CI as suggested and added a note under the table that the 95% confidence interval of the spearman’s rank correlation is not estimable.
